# Design and implementation of multi-signal and time-varying neural reconstructions

**DOI:** 10.1038/sdata.2017.207

**Published:** 2018-01-23

**Authors:** Sumit Nanda, Hanbo Chen, Ravi Das, Shatabdi Bhattacharjee, Hermann Cuntz, Benjamin Torben-Nielsen, Hanchuan Peng, Daniel N. Cox, Erik De Schutter, Giorgio A. Ascoli

**Affiliations:** 1 Center for Neural Informatics, Structures, & Plasticity, Krasnow Institute for Advanced Study, George Mason University, Fairfax, VA 22030, USA; 2 Allen Institute for Brain Science, Seattle, WA 98109, USA; 3 Neuroscience Institute, Georgia State University, Atlanta, GA 30303, USA; 4 Ernst Strüngmann Institute (ESI), Frankfurt/Main D-60528, Germany; 5 Frankfurt Institute for Advanced Studies (FIAS), Frankfurt/Main D-60438, Germany; 6 University of Hertfordshire, Hatfield AL10 9AB, UK; 7 Okinawa Institute of Technology, Okinawa 904-0495, Japan

**Keywords:** Neuronal development, Cellular neuroscience, Biophysical models

## Abstract

Several efficient procedures exist to digitally trace neuronal structure from light microscopy, and mature community resources have emerged to store, share, and analyze these datasets. In contrast, the quantification of intracellular distributions and morphological dynamics is not yet standardized. Current widespread descriptions of neuron morphology are static and inadequate for subcellular characterizations. We introduce a new file format to represent multichannel information as well as an open-source Vaa3D plugin to acquire this type of data. Next we define a novel data structure to capture morphological dynamics, and demonstrate its application to different time-lapse experiments. Importantly, we designed both innovations as judicious extensions of the classic *SWC* format, thus ensuring full back-compatibility with popular visualization and modeling tools. We then deploy the combined multichannel/time-varying reconstruction system on developing neurons in live Drosophila larvae by digitally tracing fluorescently labeled cytoskeletal components along with overall dendritic morphology as they changed over time. This same design is also suitable for quantifying dendritic calcium dynamics and tracking arbor-wide movement of any subcellular substrate of interest.

## Introduction

Neuroscientists have long recognized the importance of neuronal structure in defining circuit function. Ramón y Cajal began by drawing the complex shape of Golgi-stained neural arbors^1,2^, and neuromorphological investigations have thrived thereafter on numerous animal species, developmental phases, and brain regions. In parallel to continuous improvements in labeling and imaging techniques, methods to trace axonal and dendritic branching evolved from pencil-on-paper to digital encoding of tree origins, bifurcations, and terminations^3^. Even though two-dimensional (2D) analyses of neuron morphology have remained in practice^4^, the more complete and realistic three-dimensional (3D) reconstructions became the standard in the field^5^. As the number of scientific publications describing 3D digital tracings continued to increase, NeuroMorpho.Org emerged as a popular electronic repository to store, annotate, publicly share, and freely reuse these labor-intensive datasets^6^. Although individual neuroscience labs upload 3D reconstructions to NeuroMorpho.Org in more than 20 different formats depending on the specific reconstruction system they use, all data are converted to, and can be downloaded in, a common lingua franca, the *SWC* file descriptor^7^. The open availability of neuronal morphology digitally reconstructed in this form from a vast array of model systems, experimental preparations, anatomical regions, and cell types enabled a diverse array of secondary studies. Among the most flourishing applications are comparative morphometric analyses^8^, electrophysiological simulations^9^, large-scale biophysically-detailed modeling^10^, and algorithmic generation of virtual neurons^11,12^.

While the advent of high-throughput automated tracing has dramatically expanded the sheer volume of experimental data^13^, the existing representation of neural structure has two main limitations. First, 3D reconstructions describe the overall morphology of neurons, but no information on intracellular substrates. Rapid technological progress in serial immuno-staining^14^, genetic fluorophore engineering^15^, and spectral unmixing^16^ now allow simultaneous labeling of multiple subcellular components, requiring a co-evolution of the digital representation system. Second, current reconstructions are static, and do not incorporate temporal dynamics of neural structure. In the past, neuroinformatic tools have been developed to deal with subcellular^17–19^ and dynamic^20,21^ neural data. Nevertheless, major advancements in live imaging techniques^22–24^ necessitate an extension of the neuroanatomical file descriptors to annotate morphological changes over time. In both cases, the ideal data structure should capture these additional dimensions flexibly and precisely while preserving the intuitive simplicity of the original *SWC* format.

Here we present an expanded multichannel file format (*ESWC*) and the corresponding Vaa3D^25^ plugin to acquire multi-signal reconstructions that incorporate subcellular information simultaneously with the overall morphology of the neuron. This application repurposes the *ESWC* extension previously introduced in Vaa3D to explicitly incorporate specific morphological features for faster computation^26^. We then introduce a novel data structure (*SWCX)* to represent temporal branching dynamics. These next-generation neural tracing systems are well suited for studying the cytoskeletal effectors of neural growth and the genetic programs that govern cytoskeletal dynamics^27^. Specifically, fluorescently labeled multi-signal live images of developing neurons from *Drosophila* larvae can aid in elucidating the biochemical mechanisms underlying the known morphological diversity of neuron types^28^. We thus demonstrate how multi-signal and time-lapse reconstructions may be combined to describe subcellular structural dynamics in this genetically tractable experimental system.

## Results

**Figure 1:**
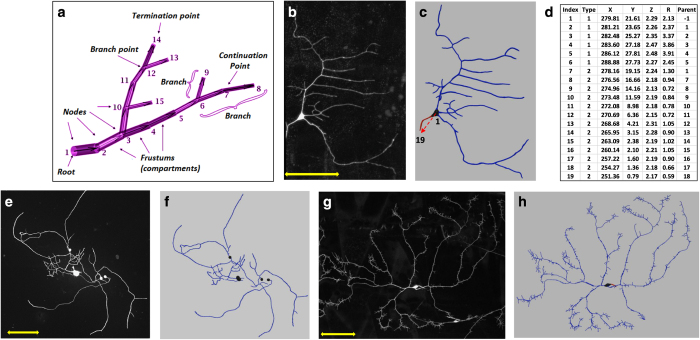
Examples of basic *SWC* reconstructions. (**a**) Representation of neural structure as a tree, along with graphical definitions of node, compartment, branch, branch point, continuation point, terminal point, and root node. (**b**) Image stack, (**c**) reconstruction, and (**d**) excerpt of swc file of Class I da neuron from the *Drosophila* larva. The *SWC* nodes marked with the red arrow in **c** are shown in **d**. The first six compartments described are soma (Type 1), followed by an axon (Type 2) coming off from the soma and going up to node 19. Dendritic nodes (Type 3) are not shown in **d**. (**e**) Maximum intensity projection of the image stack and (**f**) digital tracing of mouse sensory receptor neurons from the dorsal root ganglion (Data Citation 1). (**g**) Image stack and (**h**) reconstruction of Class III dendritic arborization (da) neuron from the *Drosophila* larva. Dendrites are depicted in blue, axons in red, and somas in black. Scale bars in **b**,**e**,**g**: 100 μm.

The classic *SWC* file format^7^ describes the three-dimensional reconstruction of (typically binary) neuronal trees (Fig. 1) as a series of interconnected nodes. Specifically, *SWC* files (see Supplementary Information for EBNF syntax) are simple text lists with each node represented as a line of seven space-separated values: (1) the node number; (2) an integer indicating the neurite type (1=soma, 2=axon, 3=dendrite etc.); (3–5) the X, Y, and Z coordinates; (6) the local radius; and (7) the number of the parent node in the path to the origin. Each node and its parent constitute respectively the ending and beginning of the connected frustums making up the neuronal tree (Fig. 1a). All digital reconstructions in NeuroMorpho.Org follow this file format, as illustrated by Class I dendritic arborization (da) neurons (Fig. 1b,c), along with an excerpt of the corresponding *SWC* file (Fig. 1d), and further exemplified by sensory neurons from the mouse dorsal root ganglion^29^ (Fig. 1e,f) and Class III da neurons from the fruit fly larva (Fig. 1g,h). These basic compartmental models allow systematic morphometric quantifications and computational simulations of current flow in dendrites, but lack the means to describe subcellular densities or structural changes.

### Multichannel neuronal reconstruction

Since the morphology of neuronal trees corresponds to the continuous bounds of their cytosolic membrane, the 3D location and thickness of each branch are typically traced from the image stack of the membrane label. The spatial distribution of intracellular constituents, in contrast, is often non-uniform and discontinuous. Thus, when an additional imaging channel reports a distinct subcellular element, its staining intensity can vary within a given branch based on the local concentration. To capture such multi-signal information in digital reconstructions, we designed an extension of the *SWC* format. In this new *ESWC* descriptor (see Supplementary Information for EBNF syntax), the first seven fields still represent the overall arbor morphology as in regular *SWC* files, but up to three additional values describe the quantity of each subcellular substrate at every node. Specifically, for each compartment delimited by the tracing point location and its parent, we report (i) the volumetric fraction in which the signal is present, measured as the proportion of voxels that are above threshold; (ii) the mean signal intensity of those voxels; and (iii) their standard deviation (s.d.). While the mean signal intensity represents the overall local concentration of the labeled substrate, the ratio above threshold and s.d. help distinguish between diffused and punctate signals independently for each channel. A compartment with ratio near 1 and low s.d. signifies homogenous substrate distribution, whereas a ratio close to 0 and high s.d. indicates strong punctate signal expression. Thus, if two subcellular components are simultaneously imaged, the *ESWC* description will consist of 13 values for each tracing point: 7 to reconstruct the arbor morphology and 3+3 to quantify each of the two parallel channels.

**Figure 2:**
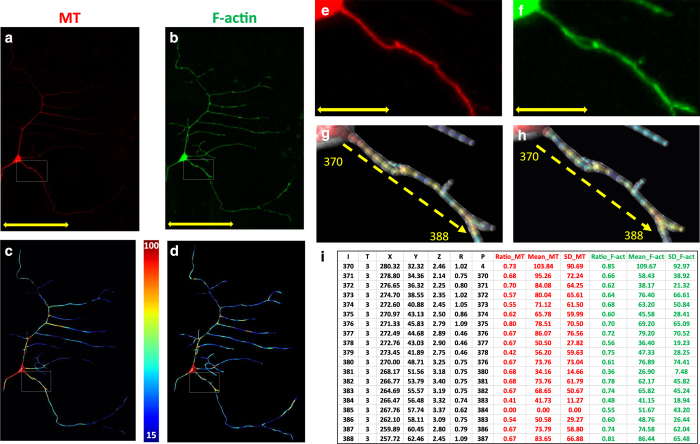
Example of an *ESWC* multi-signal reconstruction of a Class I da neuron. (**a**,**b**) Maximum-intensity projection of mCherry-tagged microtubules (**a**) GFP-tagged F-actin (**b**). (**c**,**d**) Color-coded rendering of reconstructed distributions of microtubules (**c**) and F-actin (**d**). The color represents the average signal intensity (Mean) in each compartment. Low signal intensity (15) is shown in indigo blue and high signal intensity (100+) in dark red. (**e**–**h**) are zoomed insets from (**a**–**d**), respectively with the overlaid external structure (translucent white) representing overall arbor morphology. The proportion of voxels in which the signal is present (Ratio) is represented by the internal volume of the compartment relative to the external volume of the morphology. (**i**) contains a portion of the *ESWC* file, from node 370 to 385, highlighted by the dotted yellow arrows in (**g**,**h**). Scale bars: 100 μm in (**a**,**b**), 20 μm in (**e**,**f**). The complete *ESWC* file corresponding to this neuron is available in Supplementary Information.

We implemented this design in a newly developed *multichannel_compute* plugin for the Vaa3D software suite (Vaa3D.org). Specifically, this plugin automatically generates the *ESWC* file from the multichannel image stacks and the corresponding standard *SWC* file (see Methods). In addition to outputting the *ESWC* file, the Vaa3D *multichannel_compute* plugin also saves a backward-compatible version of the *SWC* file that can be opened using any existing *SWC* viewer and other legacy tools. The beginning of this file is identical to the original *SWC* input, but the signal information from all additional channels is appended as a pseudo-comment at the end. In principle, this system can quantify any number of signals by adding three values for each imaged channel to every node. As an illustrative demonstration, we apply this multi-channel reconstruction to simultaneously quantify the distributions of microtubule (MT) and F-actin in a Class I da neuron from the fruit fly larva (Fig. 2). The polymerized forms of these cytoskeletal proteins are genetically labeled in the red and green channels, respectively (Fig. 2a,b). After reconstructing the overall morphology of this neuron (Fig. 1c), Vaa3D extracts the quantities of the two signals into an *ESWC* file for their independent visualization (Fig. 2c,d) using the custom-developed *multichannel_render* plugin (see Methods). Specifically, the subcellular components are displayed as frustums internal to the overall external structure, representing the volumetric fraction occupied, while signal intensity is coded by color (Fig. 2e–h). The underlying *ESWC* file (see Supplementary Information of digital data) stores the corresponding value for each channel (MT and F-actin) in every compartment along with the arbor morphology (Fig. 2i). The combination of this file format and software tools thus allows both quantitative acquisition and qualitative visualization of arbor-wide subcellular distributions.

### Time-lapse neuronal reconstruction

Next, we introduce a data structure to describe time-varying neural reconstructions. This description of dynamic changes (*SWCX)* also constitutes a (different) extension of the *SWC* format. An *SWCX* file (see Supplementary Information for EBNF syntax) represents the initial neuronal morphology in the first 7 fields as in the regular files and encodes every subsequent time point with additional values for each node. Thus if the initial reconstruction corresponds to the ‘zeroth’ time step, the representation of the first time step begins in the 8th field, followed by the second time step, and so on. This system requires the explicit annotation not only of the type of morphological alteration at each dynamic location, but also of the structural associations between corresponding static (unchanged) nodes across time points. We distinguish five categories of dynamic events and numerically annotate them as the following. (a) New branch extensions, including both terminal and interstitial branching: -1; (b) local scaling in branch length (stretching/contracting) or radius (thickening/thinning): -2; (c) branch rotation or deformation: -3; (d) terminal branch retraction: -4; and (e) branch re-emergence (a special case of branch extension following a retraction at the same location): -5. For the stable (unchanged) nodes, the event index simply points to the temporal parent, that is, the corresponding node in the previous time step. Thus, if node *k* in the first time point corresponds to node *j* in the zeroth time point, the 8th entry of row *k* will be *j.*

To annotate nodes over time, the morphology corresponding to each time point is mapped node-by-node onto the neuron reconstructed at the previous time point, starting from the first time point. This is achieved by associating the identity of stable nodes and tagging the changed nodes with the corresponding event label in the appropriate additional columns. Note that the *SWCX* file must include a line for every node present at any time point. Since nodes can appear and disappear dynamically, absent nodes at a given time point are annotated with ‘0’ in the corresponding field. This process is then repeated for every subsequent time point to produce the final *SWCX* file, which contains arbor-wide structural information across all time points represented in corresponding time columns (Supplementary Fig. 1).

**Figure 3:**
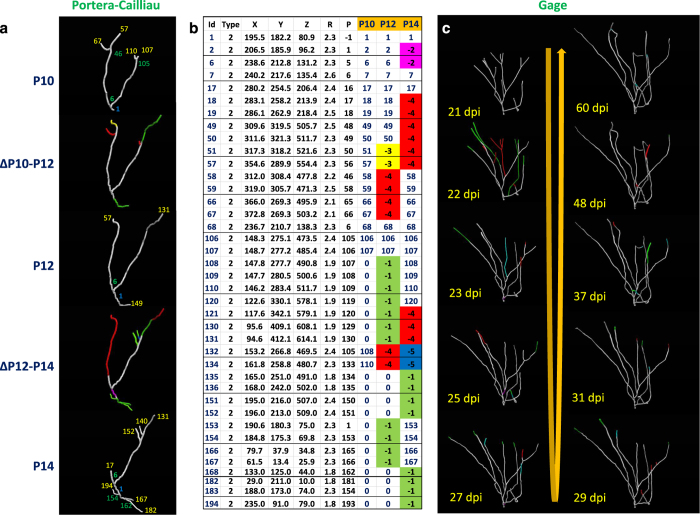
Examples of dynamic time-lapse reconstructions. (**a**) Growing axon from the neonatal mouse cortex (Data Citation 2); three static time points are displayed in gray corresponding to postnatal days 10, 12, and 14 (P10, P12, and P14, respectively); the intermediate colored images represent the dynamics of the two transitions: from P10 to P12 and from P12 to P14. The node indices of the root (in blue), branch points (in green) and terminations points (in yellow) are displayed from all three time points. In the intermediate transition images, green, red, blue, yellow, and pink branches signify elongation, retraction, re-emergence, rotation, and local scaling, respectively. (**b**) Time-varying *SWCX* file (abridged for ease of illustration: darker row borders indicate discontinuities) corresponding to the dynamic morphology of **a**. (**c**) Time-varying dendritic structure in a developing dentate gyrus granule cell (Data Citation 3). Morphological dynamics through the ten time points are color-coded: green, elongation; red, retraction; blue, re-emergence; and pink, local scaling. The complete *SWCX* file corresponding to this panel is available in Supplementary Information.

We demonstrate the general applicability of this new design by annotating the time-lapse reconstruction series from two independent experiments (Fig. 3). In both cases the original authors had separately reconstructed the morphologies at each time point and deposited the corresponding static tracing data into NeuroMorpho.Org, where they were converted into (classic) *SWC* files. We transform these static reconstructions from consecutive time points into the aforementioned *SWCX* file format. The first dataset consists of growing axons from neonatal mouse somatosensory cortex^23^. The reconstruction of one subtree over three time points is displayed along with the transition dynamics (Fig. 3a) and an excerpt of the corresponding *SWCX* file that represents the dynamical structural information using three time columns (Fig. 3b). Supplementary Fig. 2 illustrates the explicit representation of the updated 3D coordinates, neurite thickness, and structural connectivity for each time point. The second dataset involves the developing dendritic trees of adult-born granule cells in the mouse dentate gyrus^24^. When time-varying data are collected over long time spans, the majority of arbor nodes move relative to their previous positions. Even in these cases, the final *SWCX* file provides a time-based indexing of all nodes, so one can annotate any substantial movement of a node as a disappearance and re-appearance at a different location. The dynamic reconstructions captured in the *SWCX* design illustrate in this example the arbor-wide structural plasticity across ten time points by highlighting distinct categories of morphological alterations, including elongation, local scaling, retraction, and re-emergences (Fig. 3c). The complete *SWCX* file for this time series (see Supplementary Information) comprehensively encodes the 3D location, thickness, and connectivity of all nodes for each time point in addition to the type and location of any structural changes as well as the temporal correspondence between unchanged nodes.

### Combining multi-signal and time-lapse digital reconstructions

**Figure 4:**
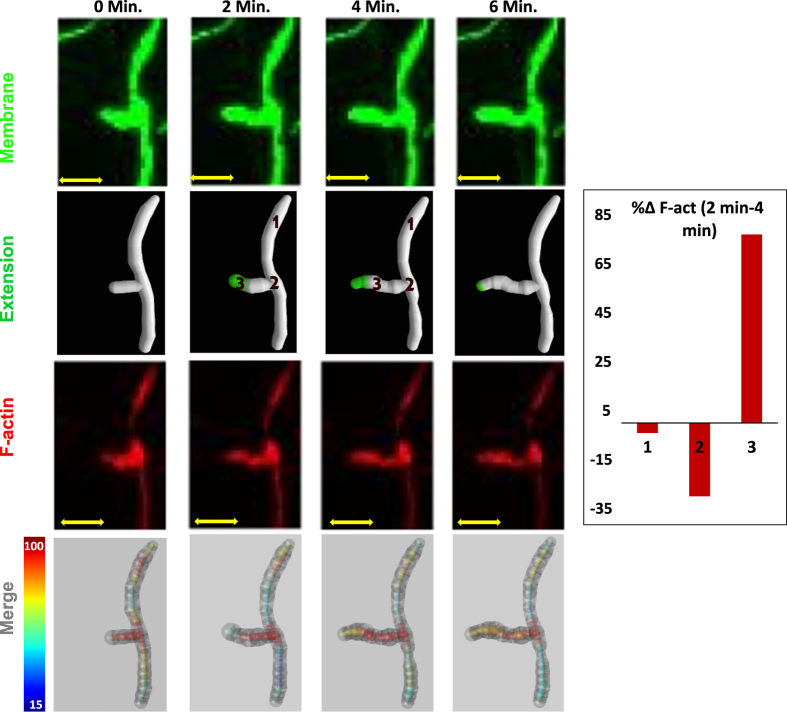
Time-lapse cytoskeletal (F-actin) dynamics of a growing branch in a fruit fly Class IV da neuron (third instar larva) at two minute time intervals. The first and third rows correspond to cytosolic membrane (labeled in green) and polymerized F-actin (labeled in red), respectively. The second row highlights growing region of dynamic branch in green. The fourth row renders the overall morphology and subcellular dynamics simultaneously. The overall morphology (translucent black external structure) changes simultaneously with the internal F-actin quantity (indigo blue: low intensity; bright red: high intensity) across four time points (min 0 to min 6). The bar graph on the right represents the relative percentage change in F-actin in three regions of the arbor: (1) a stable branch, (2) a bifurcation point, and (3) a dynamic terminal. Scale bars: 3 μm. The complete file representing the data illustrated in this figure using the *ESWC* and *swcx* formats is available in Supplementary Information.

The *ESWC* and *SWCX* formats are independent and compatible extensions of the *SWC* file system, and can thus be combined to track simultaneously temporal changes and intracellular quantities from multi-channel time-lapse experiments. In addition to the *SWC*-like representation of overall arbor morphology with 7 values per node, subcellular components from each time point are represented at each location with three columns per imaging channel and seven columns per time point. We apply this quantitative representation of multi-signal, time-varying data to describe intracellular cytoskeletal (F-actin) dynamics within a growing dendritic branch from a mature Class IV da neuron from the fruit fly larva sensory system (Fig. 4). Here, green fluorescent protein (GFP) and red fluorescent protein (RFP) genetically label the neurite membrane and F-actin, respectively. The interplay between these two signals, reconstructed across four time points, suggests that F-actin polymerization promotes branch extension. The combination of the *ESWC* and *SWCX* data structures (see Supplementary Information for digital data) quantifies both the subcellular and morphological dynamics in one and the same digital representation. Note that in both the *ESWC* and *SWCX* files the number of columns is not fixed, but instead depends on the number of imaging channels acquired (*ESWC*) or the number of time points captured (*SWCX*). Such information can be provided in the header of the files along with other useful metadata.

## Discussion

The two-dimensional neural drawings by Cajal and his pupils not only demonstrated the remarkable diversity in neuronal structures across and within brain regions and animal species, but also revealed fundamental functional principles such as directional information flow and specificity of neural connectivity. Several types of mathematical descriptors have since been developed to represent axonal and dendritic morphology. Early neuron tracing systems first listed the tracing points in ASCII files as separate text lines, recording local branch position and thickness as well as the topological type (root, bifurcation, continuation, or termination)^30^. Alternatively, the volume occupied by a neuron structure can be defined by a collection of vertices connected to polygons. Such mesh-like description is often employed in electron microscopy as well as in detailed numerical simulations of molecular diffusion^31^. This representation, however, is overly data-intensive for the majority of morphological studies and computational models. A more efficient method of capturing overall neural morphology represents branching arbors as sequences of interconnected frustums. The resulting digital reconstructions are especially suitable to describe relatively lower resolution image stacks from light microscopy. This basic data structure has remained almost unaltered in the last forty years^30^ as further efforts towards technical improvements have primarily focused on automating the tracing process^3^. Based on estimations from NeuroMorpho.Org’s literature collection, more than 180,000 neurons have been digitally reconstructed to date from at least forty species and over two hundred anatomical regions.

The *SWC* format, introduced almost two decades ago^7^, remains highly popular due to its unsurpassed simplicity. Over 70,000 neuronal reconstructions are freely available in this format from the centralized repository NeuroMorpho.Org^32,33^, and dedicated open-source analysis tools have been also developed for the *SWC* system^34^. The main rationale for expanding the *SWC* format instead of any other file system to annotate temporal and subcellular information is precisely this existing (and growing) wealth of available resources^3^. Instead of coming up with a completely new design, extending the *SWC* system enables the research community to continue leveraging a wide variety of data acquisition, analysis, and modelling tools. The *ESWC* and *SWCX* file structures also largely preserve the simplicity and readability of *SWC*. Although here for the sake of clarity we have used separate file formats (*ESWC* and *SWCX*) to describe these two conceptually distinct extensions of the existing neural description system, in the future it may become convenient to merge the two formats into a single extension along with additional expansions.

The combination of multi-signal and time-lapse digital reconstructions in principle allows the subcellular quantification of any neuronal property that can be captured over time through light-microscopic imaging, including biochemical concentrations, ion channel locations, and organelle movement, among others. Progress in multi-signal/time-lapse imaging techniques have already started to yield new findings on the subcellular and molecular organization of neurons. For instance, super resolution imaging has revealed the arbor-wide distribution of proteins associated with post-synaptic receptors^35^. Combining paired recording with array tomography enabled researchers to study the interrelation between functional plasticity and molecular composition of synapses^36^. The positions of synapses have been detected with high spatial accuracy using mGRASP^37^. Integrated systems for two-photon imaging and photo-stimulation are well suited for systematic interrogations of structural plasticity^38^. Low-intensity live-imaging of fluorophore-tagged subcellular protein complexes allow long-term tracking of mitochondrial trafficking in neurites^39^. Intensity of light emitted from calcium sensitive dyes can be measured from multiple distinct locations of the neural arbor across several time points^40^. Calcium imaging combined with whole-cell patch clamp recording has revealed that spike back-propagation triggers a path distance-dependent calcium rise in dendritic trees^41^. Advanced genetic toolkits also allow for optogenetic activation of single neurons followed by measurement of activity in functionally connected circuits^42^. Combination of live-imaging and electron microscopy has revealed the arbor-wide locations of synapses and their ultrastructural architecture^43^. Bimodal dendritic plasticity dependent on visual stimulation has also been observed through live-imaging^44^. All the above examples and many more are in principle suitable for digital reconstruction leveraging the novel data structures introduced here.

Significant progress has been made in the annotation systems for multi-signal^17–19^ and time-lapse^20,21^ neural images. However, the community needed a standard descriptor of neuro-structural dynamics^45^ capable of adding multiple dimensions of information. We demonstrated the newly introduced multichannel (*ESWC*) and time-lapse (*SWCX*) data structures in da neurons from the *Drosophila* larva. Measuring arbor-wide quantities of subcellular substrates in this model system may reveal the influence of individual molecules on mature arbor morphology. Time-varying reconstructions enable the identification and temporal linking of dynamic changes. Combining the multi-signal and time-varying systems allow one to measure the exact changes in cytoskeletal quantity within branches as they elongate or retract across the whole arbor, at the limit of light-microscopic resolution. Subcellular concentrations of growth mediating cytoskeletal proteins can be used as fundamental determinants of dendritic growth in computational simulations, and time-varying reconstructions can be used to improve and validate data-driven models. This type of dynamic data can then be analyzed, visualized and reproduced via simulation^46^. Notably, time-lapse imaging can capture information across a broad range of temporal scales depending on the dynamics of the biological phenomena under consideration. Dendritic spines and axonal varicosities can turn over in minutes while arbor structures change over weeks. The *SWCX* system is suitable to represents any and all time scales as illustrated in this report.

A complementary augmentation in neuron description not addressed in the present work is the annotation of circuit connectivity^47^. Connectivity columns can be added by annotating pre-synaptic and post-synaptic neuron pairs. Useful anatomical information also includes the location and orientation of reconstructed neurons relative to each other or to tissue layer boundaries. This additional knowledge, if available, can also be encoded in the header of the new augmented files. While the data trends in NeuroMorpho.Org suggests that most of the reconstructions in the near future will still be in the basic 3D static format, we predict that the number of new time-lapse and multichannel datasets will soon start to increase. Availability of simple file formats for these data may also facilitate the development of new tools and resources for the analysis of live and multi-signal neural images^48^.

## Methods

### Multichannel reconstruction

Multi-signal *ESWC* reconstructions were generated starting from the multi-channel image data and the standard *SWC* reconstructions. First the basic *SWC* file was created by tracing the overall neuronal structure in neuTube^49^. This file was then used as input in the *multichannel_compute* plugin of Vaa3D^25^ along with the image stacks for each channel. The plugin interface asks for a primary channel, a secondary channel, and their intensity thresholds. Within each compartment (frustums defined by the basic *SWC* file), the plugin then identifies the voxels with intensities above the input threshold for the primary channel (in this case the overall morphology signal) and then checks the intensity of the same voxels for the secondary signal. We ran the plugin twice, once with microtubule and then with F-actin as the secondary signal, using 15 (on a 0–255 scale) as the voxel intensity threshold (any voxel below 15 is not considered). The plugin outputs two multichannel files. The first file details the multichannel data in the *ESWC* format with three additional columns for each channel, annotating (1) fraction of voxels above threshold, (2) mean intensity, and (3) standard deviation of intensity. The second file, in a back-compatible *SWC* format with the regular 7 columns, appends the fraction and mean of subcellular channels at the end of the basic neuron tree after a #CHANNELSWC tag. The Vaa3D *multichannel_render* plugin uses the simple multichannel file as input and generates a multichannel render file as output, representing the secondary signal distribution as a collection of internal frustums, and the overall morphology as the connected external frustums. The intensity values for each frustum can be color-coded using the *Color_render_eswc_feature* plugin, where the input parameters are the lower and upper limits of the intensity values.

### Time-lapse reconstruction

We created the *SWCX* files for both examples of time-varying reconstructions illustrated in Fig. 3 by manually annotating each dynamic event along with the temporal correspondence of the static structure. First we ‘reverse-generated’ the static tracings into image stacks for every time point. Next we traced the first time point into a basic *SWC* file using neuTube. We then opened the image stack from the 2nd time point along with the 1st time point *SWC* in neuTube and started annotating the structural changes. Since neuTube only handles the standard *SWC* format, we temporarily repurposed the neurite type column (2nd field) of the *SWC* file to tag elongation, local scaling, retraction, re-emergence, and movement events in a ‘time-coded’ manner. The header of the *SWC* file is used to mark global changes across time points such as overall skeleton scaling. Next we compared the original *SWC* file (representing the first time point) and the edited *SWC* file (representing the second time point with all structural annotations). The ordering of nodes remains unchanged across all time points, and new branches are simply inserted after their corresponding parent nodes in case of terminal extension, and after the entire subtree in case of interstitial extension. This allows for a straightforward assignment of temporal correspondence for all nodes present in both time points. After this step we repeated the process by mapping the (edited) *SWC* file from the 2nd time point onto the image stack from the 3rd time point and continue until the last time point. At the end, we consolidated the series of annotated *SWC* files (one for each consecutive pair of time points) into a final *SWCX* file with a time column for each time point and with numeric codes for each kind of structural change. Additional columns for each time point are allocated to explicitly update the 3D coordinates, radius, and structural connectivity of each node (as well as any subcellular information in case of multi-channel neural images). The intermediate dynamic *SWCX* files from each time point can be used as input for the *multichannel_compute_plugin* along with the multi-signal image stacks to combine temporal and sub-structural information, as shown in Fig. 4.

### *Drosophila* strains and confocal imaging

*Drosophila* stocks were reared at 25 °C on standard cornmeal-molasses-agar media. The following fly strains were used in the study *GAL4*^*221*^*,UAS-mCD8::GFP* (Class I); *GAL4*^*19-12*^*,UAS-mCD8::GFP* (Class III)*; GAL4*^*477*^*, UAS-mCD8::GFP/CyO,tubP-GAL80; GAL4*^*ppk1.9*^*, UAS-mCD8::GFP* (Class IV); *UAS-GMA::GFP; GAL4*^*221*^*,UAS-mCherry::Jupiter; UAS-LifeAct-Ruby.* Fluorescently labeled da neurons from age-matched third instar larvae were imaged via *in vivo* confocal imaging using previously established protocols^21^. Briefly, larvae were placed on a microscope slide, immersed in 1:5 (v/v) diethyl ether:halocarbon oil 700 and covered with a 22×50 mm coverslip. Neurons were visualized on a Zeiss LSM 780 confocal microscope. Three-dimensional z-stacks were collected using a 20X/0.8 N.A. air objective at step-size of 1.0–2.0 μm and 1024×1024 resolution. For time-lapse imaging, images were acquired in time series mode with a time interval of 2 min between frames at a step-size of 2 μm and 1024×1024 resolution.

## Additional Information

**How to cite this article:** Nanda, S. *et al.* Design and implementation of multi-signal and time-varying neural reconstructions. *Sci. Data* 5:170207 doi:10.1038/sdata.2017.207 (2018).

**Publisher’s note:** Springer Nature remains neutral with regard to jurisdictional claims in published maps and institutional affiliations.

## Supplementary Material

Supplementary Information

Supplementary Figures
