# Supplementary material for: Design and implementation of multi-signal and time-varying neural reconstructions
Source: Sci Data. 2018 Jan 23;5:170207. doi: 10.1038/sdata.2017.207 (PMC5779069; doi:10.1038/sdata.2017.207)
Supplement: Supplementary Information [file sdata2017207-s1.pdf]

Design and implementation of multi-signal and time-varying neural reconstructions

Supplementary information

Table of Contents

1. *Digital data corresponding to Figure 2:* **Page 2-25**

2. *Digital data corresponding to Figure 3c:* **Page 26-57**

3. *Digital data corresponding to Figure 4:* **Page 58-60**

4. *EBNF representation of SWC:* **Page 61**

5*.* *EBNF representation of ESWC:* **Page 62-63**

6. *EBNF representation of SWCX:* **Page 64-65**

#Multi-signal ESWC: Id Type X Y Z R P MT_Ratio MT_Mean MT_SD F-act_Ratio F-act_Mean F-act_SD

1 1 279.812387 21.6104618 2.29493965 2.23689681 -1 0 0 0 0 0 0

2 1 281.20653 23.6501446 2.26054565 2.4906 1 0.739837 103.062 90.1345 0.806233 104.753 89.6943

3 1 282.479108 25.265951 2.35221752 3.5357032 2 0.765842 104.856 91.0807 0.865533 111.816 93.8318

4 1 283.603436 27.1751738 2.47170109 4.04851181 3 0.755028 105.26 91.6592 0.873131 112.774 94.0661

5 1 286.123686 27.805177 2.47792166 4.1062878 4 0.741599 105.252 91.6159 0.875114 112.45 93.9337

6 1 288.882322 27.7349658 2.26876463 2.5752804 5 0.747622 108.506 92.2125 0.892557 116.293 95.1637

7 3 291.766674 27.7685889 2.22058338 1.58551596 6 0.71086 107.567 90.2729 0.857546 116.878 94.0342

8 3 294.394257 28.1134184 2.16091572 0.7571424 7 0.655844 97 72.4263 0.811688 98.448 79.8689

9 3 297.339095 28.8646308 2.19841704 1.01617666 8 0.553846 101.194 76.9624 0.630769 61.4634 45.4426

10 3 300.276817 29.2602211 2.14144753 0.74718593 9 0.610169 92.6389 71.2344 0.610169 64.8889 36.0307

11 3 303.232922 29.4878145 2.1799866 0.74729267 10 0.608696 50.6429 13.0964 0.565217 48.8462 22.5178

12 3 306.285093 29.8675124 2.18395377 0.74963502 11 0.527778 51.8421 28.2401 0.416667 40 19.8964

13 3 307.641877 30.9948054 2.37356552 0.7742208 12 0.5 100.682 78.4755 0.568182 64.32 43.507

14 3 309.64681 32.3906681 2.30703092 0.92765955 13 0.4 115.636 88.9885 0.818182 65.3111 46.3905

15 3 311.890722 33.4567042 2.31421808 1.04928978 14 0.238095 27.8 6.33719 0.714286 41.5333 23.6639

16 3 315.063272 33.4254531 2.28309151 0.933975 15 0.304348 28.2857 4.62028 0.782609 47.4444 30.4325

17 3 309.094134 30.3438693 2.18465944 0.80057372 12 0.561404 99.6563 80.5523 0.596491 69 45.8296

18 3 312.044902 30.6372857 2.18699586 0.99741414 17 0.527778 89.0263 74.1618 0.666667 69.2708 50.8227

19 3 314.647579 30.7289042 2.19529193 1.21869209 18 0.589286 75.3636 62.3265 0.625 72.2571 47.0497

20 3 314.07652 28.663841 2.20247316 0.81692866 19 0.575758 66.9474 58.6941 0.606061 62.8 40.6271

21 3 313.030468 27.1286826 2.14297154 0.73795885 20 0.444444 22.75 3.89711 0.444444 22 3.53553

22 3 311.64522 25.1250542 2.18543034 0.62265 21 0 0 0 0 0 0

23 3 316.554667 31.615795 2.37565288 0.97776805 19 0.576923 74.9 59.3713 0.653846 63.3529 41.1474

24 3 317.837326 31.8560786 2.1916687 0.99870688 23 0.577778 76.8846 60.6962 0.711111 75.5938 60.9794

25 3 318.462941 33.3005673 2.24584518 0.85559226 24 0.576923 71.5667 55.0343 0.826923 82.8605 69.3188

26 3 319.175727 35.3921376 2.25482913 1.16745096 25 0.5 58.7143 39.0923 0.857143 74.1875 67.6697

27 3 320.319624 38.1461482 2.15742295 1.05894975 26 0.354839 33.8182 10.1786 0.806452 33.2 16.127

28 3 321.470044 40.8725843 2.34344705 1.06825985 27 0.5 30.8333 9.57282 0.75 31.7778 15.4652

29 3 322.568873 43.6182336 2.51938422 1.00951134 28 0.357143 35.6 9.18912 0.857143 45.7917 33.6959

30 3 323.26802 46.5308124 2.57807936 1.058505 29 0.431373 31.0909 8.15146 0.823529 46.0714 28.7844

31 3 323.559776 49.4731598 2.39654427 1.058505 30 0.5 28.6364 8.56815 0.727273 36.2813 20.2426

32 3 323.575194 52.4369145 2.33379301 1.058505 31 0.533333 29.75 4.46514 0.733333 37.2727 11.0049

33 3 323.402038 55.3966368 2.33824051 1.058505 32 0.571429 25.625 7.5488 0.642857 35 10.3495

34 3 323.158908 58.3453886 2.42256511 1.058505 33 0.555556 24.2 7.44043 0.722222 29.8462 8.94295

35 3 322.398089 61.20353 2.25309164 0.74718 34 0.357143 29.8 6.67533 0.714286 29.4 10.5565

36 3 322.019755 64.144217 2.21724479 0.74718 35 0.375 20.6667 2.86744 0.25 34 14

37 3 321.576784 67.072451 2.19197706 0.74718 36 0 0 0 0.777778 29 8.60233

38 3 320.707446 69.903433 2.2333566 0.74720965 37 0.333333 32.4286 8.926 0.952381 41.7 23.8476

39 3 320.290567 72.837597 2.20722902 0.74787974 38 0.333333 33.125 7.88095 0.625 41.3333 24.1182

40 3 319.548724 75.707124 2.27505636 0.76244382 39 0.409091 35.8889 22.3032 0.681818 30.2667 13.2387

41 3 318.859065 78.577837 2.44311849 1.12327839 40 0.218182 31 20.8527 0.709091 48.0769 32.6012

42 3 318.914214 81.538093 2.57121242 0.7467649 41 0.371429 25.8462 8.04646 0.857143 53.9333 38.4854

43 3 318.665747 84.491826 2.51655561 0.73795885 42 0.111111 17 0 0.666667 34.6667 16.0174

44 3 318.2631 87.428955 2.45311647 0.62265 43 0.111111 16 0 0.888889 53 30.6716

45 3 320.770304 32.2891465 2.19401698 1.24754747 24 0.572917 77 54.0569 0.760417 82.1233 62.6875

46 3 323.686678 32.8108679 2.3182149 1.08639379 45 0.56962 77.8444 56.4659 0.721519 69.2105 47.0195

47 3 326.468441 33.833022 2.39588604 0.76709887 46 0.565217 78.5769 70.0797 0.652174 55.7333 38.8595

48 3 329.532472 34.9416355 2.52652394 1.22598006 47 0.666667 89 65.2294 0.706667 67.8868 51.0152

49 3 330.72796 33.8596477 2.70696198 0.78936602 48 0.675325 97.9038 70.4331 0.792208 87.5738 68.2203

50 3 332.531273 33.6263022 2.66130098 0.74718 49 0.545455 68.5833 59.2417 0.727273 62.3125 55.4016

51 3 335.467216 33.2123882 2.53577474 0.74716221 50 0.333333 19.8 3.70945 0.733333 39.0909 26.9155

52 3 338.40138 32.8340542 2.56732234 0.7467649 51 0.0625 16 0 0.75 42.6667 20.0139

53 3 341.36638 32.8340542 2.595561 0.73797071 52 0.125 19.5 3.5 0.5 60.75 27.653

54 3 344.26022 33.4270542 2.60053034 0.62265 53 0.166667 24.5 4.5 0.75 61.1111 38.6651

55 3 330.835886 31.9755681 2.78115221 0.81980471 49 0.722222 72.3846 63.321 0.722222 73.0769 59.6599

56 3 330.62122 29.8690542 2.77843034 0.8129437 55 0.588235 33.6 13.6982 0.794118 40.7407 23.0098

57 3 331.246242 28.4102742 2.48066725 1.01490171 56 0.432432 36.125 12.8105 0.837838 36.6774 20.8596

58 3 332.331432 26.0697032 2.24655085 0.74718 57 0.318182 42 12.9063 0.681818 37.1333 22.494

59 3 333.27786 23.2100793 2.27678792 0.74718 58 0.409091 32.3333 7.65942 0.681818 37.2 29.3989

60 3 333.58622 20.3810542 2.18543034 0.74718 59 0.4375 33.1429 8.27092 0.625 43.9 32.6173

61 3 334.877774 18.5559188 2.29866369 0.74720965 60 0.277778 27.2 8.44748 0.611111 30.5455 15.1739

62 3 335.589374 16.0676315 2.20171412 0.74787974 61 0.333333 20.6 3.13688 0.6 25.1111 8.99931

63 3 335.816493 13.1167449 2.20171412 0.76234894 62 0 0 0 0 0 0

64 3 335.95822 10.1715511 2.18543034 1.11948319 63 0.275862 21.625 5.52127 0.724138 43.0952 31.5533

65 3 335.89299 7.4571529 2.22020386 0.73795885 64 0.225806 21.7143 5.84843 0.903226 49.8571 31.9394

66 3 335.546678 4.48873722 2.21448734 0.62265 65 0 0 0 1 29.4 10.1311

67 3 332.99322 19.7880542 2.18543034 0.73795885 60 0 0 0 0.5 35 0

68 3 330.62122 18.0090542 2.18543034 0.62265 67 0 0 0 0 0 0

69 3 329.142278 28.6013388 2.35585261 0.74718 56 0.0967742 22.3333 2.49444 0.870968 34.4444 19.8108

70 3 326.574588 27.1959881 2.18543034 0.74718 69 0.0714286 22 5 0.964286 69.1852 56.6006

71 3 324.049001 25.6422688 2.18543034 0.74716221 70 0 0 0 0.727273 38.25 17.2028

72 3 321.937921 23.5998582 2.18543034 0.7467649 71 0 0 0 0.571429 21 1.58114

73 3 320.630356 21.0560068 2.19769951 0.73796478 72 0 0 0 0.454545 30.6 12.2735

74 3 319.551096 18.6324158 2.21131479 0.62265 73 0 0 0 0.666667 29.75 16.3153

75 3 331.699294 36.6033401 2.5640727 1.0412487 48 0.616667 92.4054 70.3702 0.75 77.6111 62.7054

76 3 333.614684 38.8655165 2.63492434 0.9777977 75 0.626506 86.4231 66.4551 0.686747 68.8421 50.9722

77 3 334.732489 40.2539667 2.80910623 0.74731046 76 0.5625 87.7778 65.5282 0.604167 68.1379 43.5143

78 3 336.152724 41.6653067 2.61953599 0.74742313 77 0.571429 52.5625 23.9243 0.464286 49.6154 23.1833

79 3 337.787625 43.5384158 2.54732638 0.75256444 78 0.481481 51.6154 23.0335 0.444444 45 23.6326

80 3 339.966307 46.2514501 2.65618932 0.85482136 79 0.512195 50.2857 20.4524 0.487805 50.45 29.8068

81 3 341.500398 47.5754412 2.71328929 0.74723337 80 0.463415 43.3158 19.4342 0.487805 52.4 28.0792

82 3 343.771588 49.4812246 2.72775256 0.74840751 81 0.555556 40.7333 15.1634 0.444444 50.5 21.2936

83 3 346.266932 51.0821467 2.77666913 1.08552801 82 0.756757 61.6786 48.3167 0.756757 54.8571 35.0863

84 3 348.395802 53.1346383 2.99125211 1.08491129 83 0.659091 56.1724 41.6298 0.727273 53.6563 36.5142

85 3 350.352702 55.3614126 3.05106802 1.12190263 84 0.692308 48.0222 30.7011 0.830769 42.9815 27.1862

86 3 352.309009 57.588602 3.11090172 0.95617692 85 0.6 49.2778 26.5608 0.7 41.8571 21.1813

87 3 354.617558 60.738025 3.12719143 1.24209187 86 0.576471 63.0612 40.2104 0.8 55.1618 38.2436

88 3 353.90833 63.49251 2.98551187 0.95271973 87 0.726027 65.3396 43.4659 0.849315 66.0484 49.9627

89 3 353.496195 65.356309 2.82578732 0.95405398 88 0.761905 66.3125 51.6015 0.619048 46.3077 26.4295

90 3 352.922764 68.264974 2.82073496 0.98288564 89 0.777778 71.5238 45.8015 0.62963 42.9412 21.4845

91 3 352.364158 71.176604 2.82463097 1.01650874 90 0.742857 68.8846 45.4519 0.685714 51.125 33.5364

92 3 351.805552 74.088827 2.82853884 0.9526545 91 0.787879 70.6538 47.3814 0.69697 47.9565 27.7465

93 3 351.380964 77.022991 2.81239738 0.9526545 92 0.678571 66.1053 32.5008 0.571429 40.875 16.4692

94 3 350.963492 79.958341 2.79517666 0.9526545 93 0.591837 78.931 35.5197 0.571429 73.25 32.3857

95 3 350.546613 82.893691 2.77797373 0.9526545 94 0.641026 79.4 37.9726 0.512821 57.35 25.2928

96 3 349.868221 85.772113 2.56687759 0.9526545 95 0.722222 71.7692 42.9466 0.555556 37.65 14.2101

97 3 349.34223 88.688487 2.66665577 0.9526545 96 0.777778 71.7857 34.4999 0.75 37.8148 20.155

98 3 348.934839 91.62443 2.7340858 0.9526545 97 0.88 54.6364 20.6145 0.6 27 7.35754

99 3 348.477043 94.539025 2.44557351 0.9526545 98 0.864865 48.25 22.525 0.513514 27.3158 7.05656

100 3 347.723933 97.402029 2.60197726 0.95266043 99 0.8 40.1667 16.7075 0.6 23 4.2947

101 3 347.159397 100.308915 2.74803909 0.95271973 100 0.833333 32.9333 12.353 0.277778 24.2 5.1923

102 3 346.494051 103.198011 2.78183416 0.95403619 101 0.742857 34.9615 13.4264 0.6 32.6667 10.3984

103 3 345.859541 106.092444 2.67255612 0.98308133 102 0.772727 27.8824 7.93267 0.681818 34.6667 11.948

104 3 345.580238 109.039654 2.50937438 1.15602385 103 0.466667 28.2857 6.77631 0.833333 85.6 69.8398

105 3 345.133116 111.97026 2.50396029 0.9526545 104 0.365385 28.3158 9.27392 0.903846 90.766 64.6757

106 3 344.624322 114.890192 2.52360045 0.9526545 105 0.529412 34.2222 9.34259 0.764706 42 33.5055

107 3 344.065123 117.802415 2.52159018 0.9526545 106 0.923077 38.25 10.4413 0.692308 26.5556 6.05734

108 3 343.663069 120.736579 2.54882667 0.9526545 107 0.9 46.1111 15.6875 0.6 29.8333 2.67187

109 3 343.233144 123.62271 2.53860928 0.9526545 108 0.857143 36.4167 14.5571 0.642857 26 4.94413

110 3 343.452554 126.660056 2.51920632 0.9526545 109 0.9 33.1111 6.80595 0.8 24 4.79583

111 3 343.871805 129.630986 2.47605964 0.9526545 110 0.526316 29.3 7.77239 0.736842 35.7143 22.695

112 3 344.113749 132.538465 2.39924242 0.9526545 111 0.4375 22.8571 2.58725 0.6875 40.2727 24.2753

113 3 344.573917 135.465513 2.443753 0.9526545 112 0.52381 24.4545 5.54918 0.761905 45.875 35.9459

114 3 344.980715 138.413909 2.42429074 0.74718 113 0.275862 20.875 5.34877 0.862069 52.28 37.6123

115 3 344.980715 141.376537 2.33110672 0.74716221 114 0.333333 22.25 3.63146 0.666667 28.25 11.2667

116 3 344.980715 144.336793 2.40704037 0.7467649 115 0.75 25.3333 5.02217 0.625 22.8 5.15364

117 3 345.079153 147.204541 2.61808907 0.73795885 116 0.857143 23 3.82971 0.428571 16.3333 0.471404

118 3 345.176405 150.133368 2.75375561 0.62265 117 0.75 22.8333 3.80424 0.625 31 12.506

119 3 356.499147 61.886073 2.97918456 1.04891619 87 0.671233 68.4694 43.6601 0.808219 71.322 50.4221

120 3 358.767965 62.60301 2.93227233 0.74732825 119 0.684211 48.0385 34.3752 0.605263 57.7826 45.6689

121 3 360.700552 62.930346 2.80062633 0.75041778 120 0.645161 53.35 40.6931 0.451613 59.7143 44.3564

122 3 363.184629 63.451593 2.67011296 0.81790711 121 0.7 25.2143 6.72074 0.45 35.5556 17.9574

123 3 365.996042 64.920454 2.56667004 1.10889814 122 0.654762 80.3455 63.5525 0.77381 80.6923 71.8085

124 3 365.569082 66.714279 1.59243034 0.97508176 123 0.524752 75.8679 62.4355 0.762376 72.7662 68.9846

125 3 364.42222 69.412429 1.59243034 0.9526545 124 0.459459 42.1176 30.3468 0.702703 42.4231 48.0314

126 3 364.105558 72.32821 1.59243034 0.9526545 125 0.678571 37.9474 17.3037 0.642857 27.1667 9.65085

127 3 363.23622 75.122426 1.59243034 0.9526545 126 0.513514 39.0526 16.8319 0.621622 36.6957 12.973

128 3 362.366289 77.862679 1.59243034 0.9526545 127 0.625 34.6 14.005 0.625 31.15 11.7187

129 3 362.05022 80.754147 1.59243034 0.9526545 128 0.791667 30.8421 9.00446 0.375 20.6667 2.21108

130 3 361.415117 83.580978 1.59243034 0.95266636 129 0.574468 35.7407 17.1045 0.617021 59.3448 49.4112

131 3 361.026702 86.414925 1.91116784 0.95285612 130 0.452381 39.6316 18.5052 0.809524 56.7353 46.6566

132 3 361.026702 89.379925 1.91116784 0.95719095 131 0.473684 35.7778 10.5702 0.789474 36.4667 22.1717

133 3 361.026702 92.334844 2.11478625 1.04432637 132 0.472222 36.5882 18.1726 0.583333 35.4286 25.0838

134 3 360.86422 95.266636 2.17374824 0.9532475 133 0.512821 38.2 17.0693 0.74359 51.2414 38.0532

135 3 360.86422 98.231043 2.18543034 0.68541312 134 0.454545 35.1 12.2674 0.818182 70.5556 40.9081

136 3 361.45722 101.076257 2.18543034 0.99186959 135 0.324324 26.9167 8.55822 0.891892 61.2727 36.9523

137 3 362.05022 103.966539 2.18543034 0.74718 136 0.275862 25.5 9 0.896552 62.9615 40.7766

138 3 362.624244 106.709164 1.79645792 0.74718 137 0.272727 26.3333 5.31246 0.727273 40.125 12.6436

139 3 363.208349 109.647479 1.62138653 0.74716221 138 0.357143 27.4 6.65132 0.5 23.1429 6.89572

140 3 363.23622 112.483798 1.59243034 0.7467649 139 0 0 0 0 0 0

141 3 363.954343 115.336128 1.75206594 0.73795885 140 0.3 23 3.55903 0.8 33.875 12.5244

142 3 364.42222 118.225817 1.98776565 0.62265 141 0.363636 22.5 4.82183 0.818182 35.1111 19.5587

143 3 367.852132 64.602606 2.36646138 0.80607083 123 0.670732 77.6182 63.3075 0.756098 92.1613 77.7679

144 3 369.887308 64.387347 2.06685406 0.82190986 143 0.675 86.1111 68.0851 0.7 81.3929 75.5274

145 3 371.976447 64.253329 1.9507921 0.82216485 144 0.9 44.3333 14.3604 0.6 28.1667 10.8077

146 3 374.909425 64.062976 1.811615 0.82782207 145 0.652174 54.2667 12.2337 0.521739 50.75 33.7321

147 3 377.576739 64.363627 1.76969583 0.85479764 146 0.709677 60.5455 42.7431 0.483871 66.4667 48.0248

148 3 378.647104 66.945549 1.78468687 0.9526545 147 0.604167 58.7931 45.7168 0.666667 52.5313 44.122

149 3 379.868684 69.640734 1.76361165 0.9526545 148 0.580645 51.9444 24.1419 0.516129 35.25 18.4746

150 3 379.910787 72.590909 1.91709191 0.9526545 149 0.64 61.1875 27.4094 0.56 38.1429 17.48

151 3 380.150359 75.520329 2.0232567 0.9526545 150 0.62069 56.8889 19.8239 0.482759 28.2143 4.53838

152 3 380.541739 78.476434 2.0698665 0.9526545 151 0.777778 45.3571 24.1176 0.611111 33.9545 17.6106

153 3 380.905248 81.428981 2.2079762 0.9526545 152 0.833333 51 34.5138 0.666667 39.125 24.7585

154 3 381.189295 84.29495 2.18543034 0.9526545 153 0.72 50.8889 26.1977 0.56 35.0714 17.7259

155 3 381.794748 87.137199 2.36134379 0.9526545 154 0.6 56.5333 27.8205 0.56 38.6429 15.5086

156 3 382.747699 89.856697 2.77843034 0.95266043 155 0.74359 43.2759 19.6748 0.717949 30.1071 11.5243

157 3 383.620595 92.560184 2.77843034 0.95276717 156 0.806452 43.76 18.4874 0.709677 31.8182 15.1736

158 3 384.218932 95.296286 2.77845999 0.95515696 157 0.851852 43.7391 18.4632 0.518519 30.5714 17.9353

159 3 384.035695 98.481882 2.79531305 1.00968924 158 0.740741 46 19.3881 0.666667 49.8333 35.3557

160 3 383.283178 100.974261 2.79383055 0.95716723 159 0.510638 41.7083 21.9858 0.829787 87.7949 71.8885

161 3 382.80522 104.137916 2.77843034 0.9526545 160 0.580645 43.8889 13.1736 0.580645 39.8333 10.6836

162 3 383.260051 106.943399 2.76632128 0.9526545 161 0.666667 51.2143 9.9152 0.571429 31.1667 12.4689

163 3 383.560109 109.886458 2.76632128 0.9526545 162 0.933333 41.5 18.0743 0.633333 32.1053 7.14434

164 3 384.10982 112.799867 2.76632128 0.9526545 163 0.757576 40.2 17.8415 0.575758 38.7895 14.5984

165 3 384.887243 115.654569 2.61246743 0.9526545 164 0.518519 29.7143 8.84331 0.666667 34.0556 8.1341

166 3 385.640353 118.646254 2.69384482 0.9526545 165 0.3125 46.6 42.2071 0.90625 36.5862 19.4449

167 3 386.359662 121.519339 2.78242123 0.9526545 166 0.37931 51.0909 38.2443 0.827586 35.75 22.1345

168 3 387.567603 124.21571 2.78242123 0.9526545 167 0.0454545 24 0 0.954545 29.9048 10.614

169 3 388.893551 126.868199 2.78242123 0.9526545 168 0.344828 33.2 6.63023 0.758621 30.5 10.3078

170 3 390.263381 129.464946 2.5156246 0.9526545 169 0.62963 33.4118 11.1306 0.481481 29.6923 10.3432

171 3 391.2614 132.173177 2.18543034 0.9526545 170 0.478261 35.4545 14.0153 0.695652 45.125 26.2175

172 3 392.327021 135.115643 2.21105387 0.9526545 171 0.526316 26.8 6.86731 0.789474 41.5333 19.7581

173 3 393.097921 137.220793 2.1932105 1.058505 172 0.588235 34.1 18.582 0.588235 49.5 20.2793

174 3 392.88622 139.630152 2.411731 0.9526545 173 0.333333 39.5 22.1416 0.555556 42.8 20.6582

175 3 392.711285 142.579141 2.54594469 0.9526545 174 0.125 17.5 0.5 0.625 28.2 8.78408

176 3 392.291441 145.513898 2.58117482 0.9526545 175 0.4 23 4.52769 0.7 33.5714 14.391

177 3 391.709708 148.419598 2.67795242 0.95263671 176 0.555556 21.35 3.38046 0.833333 50.4 26.9179

178 3 390.430014 151.092842 2.73251435 0.95228091 177 0.571429 25.75 8.39568 0.8 40.8571 20.028

179 3 389.492481 153.905441 2.77158119 0.9443525 178 0.451613 27.5714 8.18286 0.774194 42.7917 22.2953

180 3 388.73522 156.770817 2.77843034 0.62265 179 0.117647 21.5 3.5 0.588235 23.1 5.62939

181 3 394.899455 137.331091 2.16878483 0.9526545 173 0.428571 37.2222 18.9196 0.52381 69.0909 42.5023

182 3 397.669951 138.282856 2.16878483 0.9526545 181 0.2 28.75 9.62743 0.75 56.5333 40.9062

183 3 400.367508 140.096843 2.18585137 0.9526545 182 0.0769231 19 0 0.692308 36.5556 16.9057

184 3 402.722311 141.858646 2.18585137 0.9526545 183 0.333333 20.5 1.5 0.333333 35 9

185 3 405.022558 144.002934 2.19760463 0.9526545 184 0.333333 25 6.48074 0.444444 20.25 1.63936

186 3 406.053785 146.703456 2.18543034 0.9526545 185 0.333333 29.8 3.31059 0.666667 23.4 5.78273

187 3 406.52522 149.623981 2.18543034 0.9526545 186 0.3 24.5 6.44851 0.85 28.5294 13.8484

188 3 405.926883 152.463265 2.18543034 0.95263671 187 0.103448 17 1.41421 0.896552 42.0385 25.4792

189 3 404.754522 155.086104 2.25231481 0.95232242 188 0.15 22.3333 5.4365 0.85 46.7647 30.3868

190 3 403.795641 157.783661 2.25231481 0.94444738 189 0.555556 23.2 6.5238 0.222222 21.5 3.5

191 3 402.3505 160.245797 2.25231481 0.62265 190 0.166667 16 0 0 0 0

192 3 378.831527 63.490731 1.79378349 0.70476864 147 0.666667 61.5 49.9692 0.555556 60.4 49.9691

193 3 380.057851 62.270337 1.55835063 0.74718 192 0.4375 63.7143 42.0296 0.5625 44.8333 32.2357

194 3 382.344459 60.407131 1.63552365 0.74718 193 0.487179 55 40.5099 0.589744 56.3913 45.5104

195 3 384.922823 58.9611005 1.75313334 0.74718 194 0.681818 46.7333 28.4639 0.454545 27.7 7.75951

196 3 387.648251 57.8116293 1.92051945 0.74718 195 0.692308 41.7778 19.5037 0.538462 32.1429 8.64256

197 3 389.60693 55.3108297 1.94285183 0.74718 196 0.733333 34.7273 12.9831 0.533333 39.5 16.7257

198 3 391.497414 53.3931863 1.94506372 0.74718 197 0.8 28.25 10.1704 0.5 27.2 10.1469

199 3 393.883053 51.6616263 1.70770954 0.74718 198 0.5625 35.3333 13.5974 0.5625 30.3333 17.0229

200 3 396.185079 49.8041131 1.56068705 0.74718 199 0.47619 44.4 19.4227 0.47619 28.4 10.5186

201 3 398.637727 48.0831085 1.47822447 0.74718 200 0.2 17 0 1 27.4 10.0319

202 3 400.904766 46.1753682 1.36647362 0.74718 201 0 0 0 0.363636 28.25 9.39082

203 3 402.762635 46.3806648 1.60640142 0.74718 202 0 0 0 0.416667 22.8 4.44522

204 3 405.216469 46.790665 1.5658165 0.74718 203 0.2 21.5 3.5 0.4 22 4.41588

205 3 406.543603 49.3553307 1.7376679 0.74718 204 0.434783 38.1 12.9186 0.695652 29.125 12.9994

206 3 407.554075 51.9773988 1.68933247 0.74718 205 0.16 29.75 7.46241 0.8 58.25 41.8544

207 3 408.951183 54.5757469 1.69483551 0.74718 206 0.416667 24 4.24264 0.666667 25.25 8.65664

208 3 410.689859 56.9771004 1.70864055 0.74718 207 0.5 23.1429 4.38923 0.642857 36.5556 22.677

209 3 412.484277 59.342696 1.66563026 0.74718593 208 0.4 22.6667 4.57044 0.733333 38.2727 21.9259

210 3 414.163653 61.78467 1.71449939 0.74733418 209 0.333333 22.3333 5.79272 0.555556 22.8 8.2801

211 3 415.732731 63.765883 1.69051254 0.7510345 210 0.6 24.5 5.18813 0.5 30 9.40213

212 3 417.10434 66.23217 1.59243034 0.83959312 211 0.333333 25.75 5.6734 0.5 38.8333 9.90651

213 3 417.906076 68.948703 1.58393858 0.74683013 212 0.2 22.6667 4.98888 0.8 47.25 29.1837

214 3 418.328292 71.641516 1.60335933 0.73824942 213 0 0 0 1 26.5 7.5

215 3 417.79222 74.343817 1.59243034 0.62265 214 0 0 0 0 0 0

216 3 406.660424 45.0070989 1.58886641 0.74719779 204 0.2 18.5 1.5 0.7 22.1429 5.79232

217 3 408.948811 43.0926577 1.58636988 0.7476544 216 0.25 20.5 3.5 0.875 23.2857 6.88091

218 3 411.152399 41.2454034 1.58636988 0.75766424 217 0 0 0 0.4 18.5 0.5

219 3 413.275932 39.2324649 1.58636988 0.98602854 218 0.304348 47.2857 13.1227 0.652174 63.0667 36.9585

220 3 415.450463 37.2247448 1.58636988 0.74718 219 0.391304 47.6667 10.0333 0.695652 44.3125 29.8951

221 3 417.064016 35.0191406 1.59243034 0.74718593 220 0 0 0 0.4 23.5 4.15331

222 3 418.414277 32.3833742 1.86199035 0.74726895 221 0.538462 43.1429 17.6265 0.384615 35.4 13.4253

223 3 419.198816 29.509459 1.8541331 0.74921992 222 0.428571 46.1111 18.9763 0.809524 47.2353 22.2698

224 3 418.895793 26.515995 1.86199035 0.79241404 223 0.5625 55.5556 18.2032 0.9375 35.7333 14.848

225 3 419.258116 23.8643955 1.6074451 0.79154826 224 0.535714 63.5333 51.7492 0.821429 74.4348 69.8183

226 3 418.838865 20.8159604 1.59243034 0.74796276 225 0.5 52.6429 54.7209 0.642857 72 69.1504

227 3 418.558376 17.9835552 1.59243034 0.76581206 226 0 0 0 1 18.75 1.63936

228 3 420.58525 15.8402753 2.0339307 1.04220936 227 0.245283 35 15.2467 0.886792 50.4681 38.4148

229 3 423.731115 14.9641178 2.09004036 0.74787974 228 0.22807 34 13.4964 0.859649 54.551 44.7086

230 3 426.682476 15.7037074 1.98098766 0.76250312 229 0.347826 41.5 10.0871 0.869565 51.95 40.4283

231 3 429.162402 17.4050244 1.59243034 0.94808247 230 0.428571 37.8333 17.4968 0.785714 59.4545 49.0795

232 3 431.655374 18.8721657 1.60321108 0.896616 231 0.387097 46.25 19.3482 0.741935 64.4348 46.1122

233 3 434.27762 19.9607951 1.61460854 0.896616 232 0.407407 46.8182 18.2449 0.555556 42 26.5656

234 3 437.022024 20.5730676 1.75201257 0.896616 233 0.5 50.9231 20.3223 0.423077 36.4545 23.5

235 3 439.634782 19.7927389 1.75201257 0.896616 234 0.37037 58.8 22.324 0.481481 58.6923 37.0039

236 3 442.220262 18.5343929 1.73553903 0.896616 235 0.5 44.4545 18.7876 0.409091 38.1111 15.5166

237 3 445.122997 18.0151028 1.73553903 0.896616 236 0.6 42.8889 15.1176 0.2 54.3333 11.7284

238 3 448.062498 18.2651709 1.73466139 0.89665751 237 0.65 47.8462 24.0731 0.4 54.875 48.5758

239 3 451.010894 18.4599121 1.73886576 0.89759445 238 0.55 50.4091 37.0524 0.6 71 59.9131

240 3 453.875677 18.5705066 1.75551127 0.91816562 239 0.366667 52 28.3901 0.7 47.1905 42.5817

241 3 456.715554 19.0875433 1.59243034 0.91297687 240 0.416667 45 24.0056 0.694444 39 28.8305

242 3 459.660392 19.3686253 1.59243034 0.74718 241 0.52 46.6154 23.2264 0.64 39.0625 29.2286

243 3 462.44512 20.3868656 1.59243034 0.74716221 242 0 0 0 0.6 18.3333 2.62467

244 3 465.309903 20.9740542 1.59243034 0.7467649 243 0 0 0 0.333333 18 0

245 3 468.274903 20.9740542 1.59243034 0.73799443 244 0 0 0 0.5 18 0

246 3 471.16222 21.5670542 1.59243034 0.62265 245 0 0 0 0 0 0

247 3 400.149877 44.5968022 1.53831316 0.71646853 202 0 0 0 0.222222 27.5 2.5

248 3 399.40922 42.9350383 1.59243034 0.74718 247 0 0 0 0 0 0

249 3 398.618751 40.1479383 1.59243034 0.74718 248 0 0 0 0.428571 20.6667 0.471404

250 3 398.150874 37.3487411 1.60028166 0.74716221 249 0 0 0 0.636364 39 22.0648

251 3 397.996694 34.4777909 1.58721194 0.74677083 250 0.285714 16 0 0.142857 18 0

252 3 397.845479 31.555902 1.59243034 0.73810117 251 0.125 16 0 0.375 16.6667 0.942809

253 3 398.22322 28.6830542 1.59243034 0.62265 252 0 0 0 0.5 17 1

254 3 339.419561 47.5533816 2.7518165 1.04448648 80 0.542857 67 47.9704 0.685714 70.2083 48.0095

255 3 338.530654 50.4044663 2.83278472 0.89972925 254 0.684211 39.8462 25.7483 0.684211 48.8462 32.641

256 3 337.750859 53.2560254 2.74251233 0.89972925 255 0.71875 36.8696 17.9354 0.71875 33.6087 14.3125

257 3 337.341689 56.1603615 2.65225773 0.89972925 256 0.53125 37.7059 17.4651 0.84375 34 13.9709

258 3 337.040445 59.1015229 2.82426924 0.89972925 257 0.578947 39.0909 12.5006 0.684211 33.7692 12.217

259 3 336.868475 62.060415 2.74216839 0.8997589 258 0.615385 42.375 14.4476 0.769231 34 10.8812

260 3 336.65203 65.015334 2.67694432 0.90042306 259 0.444444 48.875 5.41843 0.888889 40.125 15.0826

261 3 336.298009 67.959579 2.6632816 0.91498121 260 0.428571 33.4167 14.5686 0.75 39.1429 18.6988

262 3 335.914931 70.898487 2.67922737 1.04386383 261 0.444444 31 11.7189 0.851852 57.1739 41.1959

263 3 335.684847 73.876533 2.7411425 0.89972925 262 0.377778 26.7647 8.84194 0.822222 53.9189 35.0346

264 3 335.272712 76.807732 2.59812276 0.89972925 263 0.34375 24.1818 6.20584 0.75 41.9583 18.2105

265 3 334.963759 79.747233 2.44756599 0.89972925 264 0.333333 21 2.44949 0.888889 39.375 13.2093

266 3 334.44607 82.562204 2.50805199 0.89972925 265 0.285714 21.8333 3.28718 0.904762 42.1053 22.7709

267 3 333.365624 85.456044 2.42641961 0.89972925 266 0.375 21.3333 2.68742 0.8125 37.6923 23.3449

268 3 332.432835 88.265085 2.45355529 0.89972925 267 0.333333 32.2 10.6471 0.733333 31.8182 13.7432

269 3 331.829161 91.16782 2.45006845 0.89972925 268 0.625 32.8 11.4 0.875 30.1429 12.5405

270 3 331.224301 94.070555 2.44657568 0.89972925 269 0.526316 48.4 22.8569 0.684211 35 20.1265

271 3 330.697717 96.985743 2.43194637 0.89972925 270 0.526316 44 23.533 0.631579 30.3333 19.855

272 3 330.409519 99.936511 2.38367617 0.89972925 271 0.5 41.4545 14.7547 0.681818 34.2667 15.3209

273 3 330.080997 102.87957 2.42008637 0.89972925 272 0.533333 47.625 12.9512 0.733333 41.3636 23.8605

274 3 329.671234 105.803653 2.57459845 0.89972925 273 0.545455 48.3333 20.1632 0.772727 45.1765 29.5659

275 3 329.313655 108.736631 2.39974054 0.89972925 274 0.733333 36.6364 10.3772 0.666667 28.5 13.1472

276 3 328.709388 111.63818 2.47692542 0.89972925 275 0.6 43.5556 20.282 0.466667 32.7143 16.4726

277 3 328.038705 114.525497 2.55413402 0.89972925 276 0.75 43.5 14.3265 0.625 26.6 4.75815

278 3 327.368022 117.412814 2.63130704 0.89972925 277 0.7 26 5.37188 0.8 29 9.94987

279 3 326.563321 120.257435 2.66100448 0.89972925 278 0.615385 31.0625 12.407 0.653846 45.1176 32.7843

280 3 325.421203 122.987607 2.51777719 0.89972925 279 0.62069 29.9444 11.0276 0.724138 45.0952 32.0415

281 3 324.490786 125.795462 2.36710182 0.89972925 280 0.411765 25 5.18239 0.823529 39.5 22.0996

282 3 323.59417 128.617549 2.41531865 0.89974111 281 0.333333 30 11.1867 0.761905 63.3125 39.1052

283 3 322.670869 131.418288 2.33957476 0.89989529 282 0.37037 35.9 15.3457 0.814815 52.8636 36.1654

284 3 322.338789 134.363719 2.35952921 0.90350073 283 0.636364 30.1429 11.3191 0.681818 32.8667 15.7813

285 3 322.23027 137.32101 2.35398466 0.98400048 284 0.538462 37.4286 20.1058 0.653846 43.8824 24.9632

286 3 322.399275 140.278894 2.38690802 0.98741616 285 0.413793 42 19.105 0.827586 48 28.801

287 3 322.457982 143.236185 2.43410489 1.26412775 286 0.47619 28.7 9.11098 0.904762 51.9474 34.0634

288 3 322.663753 146.540381 2.49074232 0.89972925 287 0.514286 26.3333 6.22718 0.942857 59.7273 37.08

289 3 323.947598 149.006075 2.60005594 0.89972925 288 0.368421 26.4286 4.5937 0.842105 35.5625 15.8389

290 3 323.565706 152.012585 2.35638631 0.89972925 289 0.0769231 19 0 0.692308 32.4444 13.8813

291 3 323.247265 154.957423 2.21356226 0.89972925 290 0.157895 22 1.41421 0.842105 34.8125 15.733

292 3 322.964404 157.905819 2.11650595 0.89972925 291 0.545455 25.6667 5.55778 0.727273 26.125 5.81888

293 3 322.837502 160.866668 2.14768589 0.89972925 292 0.583333 22.1429 5.19419 0.75 22.2222 5.92129

294 3 322.884349 163.825145 2.05367167 0.89972925 293 0.285714 24.25 5.11737 0.928571 29.5385 15.6629

295 3 323.056912 166.759902 1.70820766 0.89971146 294 0 0 0 0.777778 38.8571 11.5193

296 3 323.430502 169.685171 1.89411316 0.89937345 295 0 0 0 0.8 33.5 12.7769

297 3 324.134393 172.469306 2.19284284 0.89191944 296 0 0 0 0.333333 18.5 0.5

298 3 325.28422 175.153817 2.18543034 0.933975 297 0 0 0 0.444444 21.5 4.92443

299 3 286.166382 32.9819484 2.30179473 1.13878534 5 0.727957 106.691 91.9051 0.869892 113.95 94.6728

300 3 286.220938 35.4029895 2.36256537 1.24682401 299 0.701149 78.2951 69.3668 0.724138 75.6508 67.1232

301 3 286.315225 38.3597468 2.16331737 0.74719779 300 0.76087 62.0286 51.4701 0.695652 45.75 31.3319

302 3 286.315225 41.3200621 2.3298377 0.74763068 301 0.777778 78.0714 41.7175 0.777778 35.1429 12.2699

303 3 286.315225 44.2850621 2.34399261 0.75703566 302 0.590909 71.6154 43.7819 0.590909 35.3077 14.6885

304 3 286.794369 47.2110427 2.329897 0.95444536 303 0.511628 53.4545 36.6144 0.604651 36.1154 13.4831

305 3 287.132972 50.1552877 2.41856829 0.81636531 304 0.677419 46.5714 32.4016 0.741935 38.6957 16.0066

306 3 287.471575 53.099592 2.50724551 0.74719186 305 0.566667 48.7647 29.4829 0.633333 35.2105 18.9919

307 3 287.810771 56.0437777 2.59592273 0.74742906 306 0.473684 45.6667 18.5293 0.684211 50.4615 26.0048

308 3 289.216774 58.6363144 2.8305669 0.75258816 307 0.548387 44.1176 20.4102 0.870968 66.037 35.8985

309 3 290.164981 60.131979 3.28353588 0.75718391 308 0.53125 57.2941 32.4939 0.875 62.4286 47.9162

310 3 291.488557 60.637808 2.67534915 1.01707209 309 0.644444 62.1379 36.0275 0.822222 93.8919 70.7297

311 3 292.617036 62.007638 2.55486341 0.75256444 310 0.517857 58.8276 37.3899 0.732143 85.9756 70.227

312 3 293.982715 64.785843 2.35537228 0.85479764 311 0.392857 38.6364 15.4406 0.75 48 29.8552

313 3 295.454541 67.353533 2.18085238 0.74718 312 0.4 37 15.7109 0.733333 47.5909 29.4814

314 3 296.750839 70.010173 2.21191965 0.74718593 313 0.0714286 18 0 0.642857 27.4444 12.0103

315 3 297.983093 72.6425 2.24932609 0.74725116 314 0.347826 38.375 7.24461 0.73913 47.5294 30.0844

316 3 299.505917 75.178168 2.23227734 0.74875145 315 0.307692 35.625 12.2774 0.730769 40.6842 22.4899

317 3 300.724532 77.877504 2.21245335 0.78106402 316 0.423077 33 12.5336 0.730769 45 24.6555

318 3 301.752201 80.646221 2.22319851 0.81633566 317 0.518519 28.5714 11.5184 0.777778 40.619 24.0327

319 3 302.583587 83.492028 2.26754898 0.74718 318 0.458333 31.1818 9.71239 0.75 32.8333 14.1941

320 3 303.11195 86.398914 2.29809441 0.74718 319 0.375 37.5556 10.5632 0.916667 35.8182 16.7702

321 3 303.439286 89.345531 2.31943055 0.74718 320 0.266667 31 7.81025 0.8 32 13.0192

322 3 303.512818 92.421422 2.3597249 0.74718 321 0.32 37.375 10.3795 0.84 43.0476 20.6685

323 3 303.772552 95.373969 2.31444342 0.74718 322 0.444444 32.375 7.59831 0.888889 41.6875 19.4188

324 3 304.042367 98.323551 2.19062502 0.74718 323 0.363636 23.5 1.80278 0.727273 32.375 16.5298

325 3 304.806744 101.196043 2.11223635 0.74718 324 0 0 0 0.6 20.8333 5.52016

326 3 305.242006 104.129021 2.14231924 0.74718 325 0 0 0 0.5 28 6.84105

327 3 305.326805 107.07801 2.11868226 0.74718 326 0 0 0 0.75 28 6.65833

328 3 305.177962 110.038859 2.05928145 0.74718 327 0.111111 21 0 0.555556 30.6 7.47262

329 3 304.303287 113.012754 2.03559703 0.74718 328 0.25 18.5 1.5 0.5 35.25 6.86932

330 3 303.579234 115.887618 1.97267973 0.74718 329 0 0 0 0.75 21.6667 4.26875

331 3 302.813671 118.74825 2.05698061 0.74718 330 0.285714 17.5 0.5 0.428571 25.3333 0.471404

332 3 302.019644 121.598801 2.23944671 0.74718 331 0.533333 30.75 8.45207 0.666667 34.4 13.47

333 3 301.25586 124.461212 2.28492981 0.74716221 332 0.3125 27.4 6.18385 0.4375 25 6.74007

334 3 300.684801 127.132084 2.26367669 0.7467649 333 0.285714 27.5 5.22015 0.5 28.4286 9.48468

335 3 299.765651 130.088189 2.29490407 0.73795885 334 0.181818 22.5 4.5 0.363636 19.5 3.90512

336 3 298.469353 132.888335 2.34871882 0.62265 335 0.222222 32.75 25.5869 0.666667 40.3333 23.8619

337 3 292.008025 59.1068599 2.97162381 0.74800427 310 0.605263 64.9565 39.386 0.789474 101.467 75.0239

338 3 293.480444 56.5560704 3.10759278 0.74718593 337 0.266667 33 9.87421 0.8 41.3333 26.8618

339 3 294.589947 53.8197312 2.8396991 0.74730453 338 0.285714 32.5 16.1941 0.857143 33.1667 9.62491

340 3 295.761715 51.8977589 2.68901187 0.7499671 339 0.571429 35.5833 14.4017 0.761905 47.125 26.7205

341 3 297.02184 51.1104921 2.58955391 0.62286348 340 0.454545 32.8 10.6846 0.727273 44.25 26.5695

342 3 298.770597 50.2347497 2.4285722 0.74718 341 0.45 34 11.3725 0.75 37 22.9724

343 3 301.588533 49.5800184 2.40995793 0.74718 342 0.461538 36.3333 12.9443 0.615385 32 12.9518

344 3 303.29578 49.9271013 2.58832047 0.74718593 343 0.388889 39.7143 12.7695 0.722222 43.4615 25.1629

345 3 304.86723 50.8713352 2.40210068 0.74729267 344 0.454545 29.8 12.6238 0.909091 39.3 24.9361

346 3 306.961113 52.9630834 2.22791879 0.74965874 345 0.352941 22.6667 5.55778 0.764706 56.2308 38.3589

347 3 308.649977 55.6773037 2.16558856 0.80371069 346 0.263158 28 13.9714 0.947368 49.7778 35.0659

348 3 309.86622 58.3330542 2.18543034 0.62265 347 0.25 29 11.9024 0.833333 43 30.9095

349 3 302.515985 47.8347601 2.30071547 0.74716221 343 0.470588 37.25 13.5808 0.882353 40.5333 24.1574

350 3 303.181924 44.9513569 2.19205415 0.7467649 349 0.3125 19.8 3.76298 0.9375 29.1333 11.5635

351 3 303.005803 42.1952115 2.17512993 0.73795885 350 0 0 0 1 24 0

352 3 302.878901 39.0380795 2.17579409 0.62265 351 0 0 0 1 31 3

353 3 295.962742 50.0463536 2.5882671 0.75600384 340 0.444444 34.75 6.2998 0.777778 34.5714 20.9411

354 3 294.706768 47.7934873 2.29484477 0.94004732 353 0.227273 31.4 7.68375 0.863636 42.6842 33.9892

355 3 292.413637 45.8808251 2.17147705 1.17938805 354 0.27027 35 13.4907 0.891892 46.0909 34.7836

356 3 289.410685 46.8009239 2.18242976 0.75704752 355 0.21875 36.2857 14.983 0.78125 39.16 30.1777

357 3 287.02979 48.5992557 2.18543034 1.01412488 356 0.590909 47.2692 34.1564 0.727273 36.75 15.8666

358 3 285.01359 50.7500667 2.33522807 0.75006791 357 0.518519 48.0714 33.678 0.666667 35.8333 17.8396

359 3 283.383433 53.1851619 2.69356018 0.8129437 358 0.352941 27.6667 5.76387 0.588235 38.6 22.5663

360 3 282.057485 55.8367614 2.74091123 0.74716814 359 0.368421 23.7143 2.6573 0.789474 44.6 25.3003

361 3 280.662749 58.4527809 2.76810621 0.7467649 360 0.454545 20.4 2.05913 0.818182 46.8889 23.6194

362 3 279.24963 61.059431 2.77843034 0.73795885 361 0.454545 29.2 12.2458 0.863636 47.3684 39.6262

363 3 277.84422 63.669817 2.77843034 0.62265 362 0.444444 24.625 10.0987 0.722222 47.5385 41.4926

364 3 289.15866 60.701259 2.42066751 0.74718 308 0.4 51.5 26.9861 0.733333 64.9091 39.4679

365 3 287.797132 63.111804 2.28556432 0.74718593 364 0.285714 36.25 11.099 0.714286 38.8 17.1102

366 3 286.190695 65.579277 2.16801393 0.74728081 365 0.35 26.8571 9.71639 1 39.7 22.0479

367 3 285.443515 68.333169 2.18543034 0.74953421 366 0 0 0 0.833333 20.2 2.92575

368 3 284.354174 70.843931 2.18543034 0.80288642 367 0.222222 18.5 1.80278 0.777778 32.5 18.0347

369 3 283.77422 73.750817 2.18543034 0.91915 368 0 0 0 1 47.0714 34.6275

370 3 280.318809 32.3222359 2.45971656 1.01804461 4 0.725195 103.844 90.693 0.851654 109.667 92.965

371 3 278.804287 34.3592502 2.14444811 0.74971804 370 0.678571 95.2632 72.2385 0.660714 58.4324 38.9168

372 3 276.651104 36.3172176 2.24959887 0.8024476 371 0.702703 84.0769 64.2459 0.621622 38.1739 21.3229

373 3 274.700134 38.5476092 2.35203962 1.01553622 372 0.574468 80.037 65.6074 0.638298 76.4 66.6071

374 3 272.601507 40.8753714 2.44785656 1.04805634 373 0.55 71.1212 61.5023 0.683333 63.1951 50.8432

375 3 270.968978 43.1286528 2.49555748 0.85679012 374 0.615385 65.7813 59.9931 0.596154 45.5806 28.4092

376 3 271.332487 45.8308945 2.79183807 1.08540941 375 0.802817 78.5088 70.4985 0.704225 69.2 65.0876

377 3 272.490023 44.6813047 2.88563288 0.462760003 376 0.672131 86.0732 76.5552 0.721311 79.2045 70.5207

378 3 272.76221 43.0277835 2.89803251 0.462760003 377 0.666667 50.5 27.8194 0.555556 36.4 19.2312

379 3 273.45009 41.8939675 2.74532908 0.462760003 378 0.416667 56.2 59.6302 0.75 47.3333 28.245

380 3 270.000609 48.7142384 3.25422389 0.74718 376 0.666667 73.7632 73.0376 0.614035 76.8857 74.4058

381 3 268.174169 51.5630697 3.17776247 0.74718 380 0.678571 34.1579 14.6584 0.357143 26.9 7.47596

382 3 266.774096 53.7851593 3.40478066 0.74718593 381 0.675676 73.76 61.7927 0.783784 62.1724 45.8183

383 3 264.692666 55.5696149 3.19414706 0.74727488 382 0.673913 68.6452 50.6701 0.73913 65.8235 45.2447

384 3 266.472259 56.4804629 3.31771047 0.73795885 383 0.407407 41.7273 11.2742 0.481481 41.1538 18.9446

385 3 267.76322 57.7400542 3.37143034 0.62265 384 0 0 0 0.545455 51.6667 43.1959

386 3 262.101256 58.1139407 3.0886998 0.74920213 383 0.542857 50.5789 29.267 0.6 48.7619 26.4393

387 3 259.885808 60.448048 2.80121933 0.79104421 386 0.672414 73.7949 58.8 0.741379 74.5814 62.0368

388 3 257.721358 62.462469 2.44878164 1.08540941 387 0.671233 83.6531 66.8756 0.808219 86.4407 65.4762

389 3 255.856373 65.991412 2.48005646 0.99632302 388 0.4 58.5769 54.0628 0.753846 66.6735 57.108

390 3 255.248548 68.359854 2.36849537 0.62265 389 0.0789474 25.3333 2.0548 0.921053 73.2286 62.9544

391 3 255.348765 63.336551 2.462729 1.1013196 388 0.672131 84.2439 64.1792 0.786885 77.2292 59.6522

392 3 253.806965 63.72378 2.45124852 0.859257 391 0.818182 73 55.4637 0.454545 60.7 44.6767

393 3 251.969258 64.489936 2.45243452 0.859257 392 0.722222 48.9231 24.5277 0.388889 34.5714 8.90998

394 3 249.181565 65.475502 2.25765774 0.859257 393 0.631579 58.1667 31.0237 0.421053 29.875 10.1911

395 3 246.45673 66.618213 2.00995571 0.859257 394 0.631579 75.3333 45.363 0.710526 58 42.2462

396 3 243.68149 67.617418 1.80955136 0.859257 395 0.545455 62.4444 46.1546 0.636364 45.6667 40.3996

397 3 240.532067 68.087074 1.73675468 0.859257 396 0.533333 59.75 35.5677 0.333333 34.5 12.1099

398 3 238.122708 69.568981 1.76784567 0.859257 397 0.666667 51.9444 34.8863 0.444444 38.1667 14.6221

399 3 235.708012 71.123827 1.76129302 0.859257 398 0.785714 48.9545 37.8375 0.5 28.8571 13.2442

400 3 233.006897 72.5239 1.79884771 0.859257 399 0.666667 55.875 41.5344 0.583333 30.9286 17.0313

401 3 230.815169 73.978529 1.95579702 0.85926886 400 0.53125 50.6471 27.7466 0.71875 41.5652 24.5832

402 3 227.652107 75.029918 1.83437434 0.85950606 401 0.521739 51.6667 23.3892 0.565217 36.6923 25.4902

403 3 224.890506 76.083086 1.77377567 0.86487864 402 0.652174 51.4 24.7246 0.608696 36.4286 25.2974

404 3 222.385674 77.587527 1.80861442 0.97343322 403 0.535714 53.0667 29.4765 0.607143 35.5882 23.6147

405 3 219.969199 79.29596 1.62311216 0.859257 404 0.612903 50.7368 34.5175 0.548387 32.1765 11.3357

406 3 217.545608 80.994905 1.45378101 0.859257 405 0.65 49.0769 34.1489 0.625 37.76 21.4108

407 3 214.95064 82.743069 1.3943209 0.859257 406 0.518519 37.1429 17.0246 0.444444 31.0833 11.8354

408 3 211.96192 82.045701 1.73750779 0.859257 407 0.605263 53.3478 35.6305 0.447368 34.2353 12.4169

409 3 208.956596 81.055391 2.22158555 0.859257 408 0.604651 54.6538 42.1298 0.465116 30.75 12.5534

410 3 206.780286 82.559239 2.86860192 0.859257 409 0.466667 51.7857 33.4326 0.6 39.8889 16.5961

411 3 205.239672 85.061699 3.26133989 0.859257 410 0.428571 37.5556 9.96785 0.666667 29.9286 15.7365

412 3 204.059009 87.780011 3.36222105 0.859257 411 0.653846 49.3529 30.7494 0.5 29.5385 8.97531

413 3 202.919263 90.517299 3.34696909 0.85926886 412 0.78125 47 36.2657 0.65625 31.619 14.5323

414 3 202.578288 93.462137 3.33169934 0.85953571 413 0.652174 41.3333 29.556 0.826087 36.0526 18.8022

415 3 202.237906 96.407568 3.31641773 0.86545385 414 0.575758 43.7895 26.7573 0.727273 41.0833 23.9547

416 3 202.379633 99.365452 3.16857097 0.98304575 415 0.52381 31.4545 12.8867 0.904762 44.7895 36.0315

417 3 202.827941 102.297837 3.01976355 0.85928665 416 0.5 49.7727 20.2169 0.840909 49.4865 39.3959

418 3 203.117325 105.220141 2.938908 0.85982628 417 0.428571 40.8333 10.2212 0.642857 24.2222 6.76228

419 3 203.61841 108.126434 2.91229416 0.87169814 418 0.5 39.0909 11.2448 0.5 28.8182 10.7688

420 3 205.119886 110.958602 2.96886636 1.19000868 419 0.538462 40 13.6403 0.707692 34.0217 18.8524

421 3 206.004049 113.788398 2.98280779 1.04487786 420 0.594203 40.1707 18.9774 0.724638 39.72 24.1761

422 3 206.372895 116.729678 2.91406723 0.859257 421 0.666667 43 29.4816 0.611111 36.5455 25.6102

423 3 206.284538 119.730258 2.75075503 0.859257 422 0.575758 56.3158 37.0235 0.757576 48.92 32.1446

424 3 206.104859 122.689921 2.77627775 0.859257 423 0.580645 49.7222 32.0985 0.870968 44.5926 29.254

425 3 206.193809 125.636538 3.09268476 0.859257 424 0.538462 44.4286 26.3485 0.692308 32 14.1028

426 3 206.587561 128.460404 3.10808497 0.85926886 425 0.529412 27.8889 8.59515 0.529412 23.6667 6.35959

427 3 206.738183 131.476995 3.2793493 0.85962466 426 0.5 21.3333 3.55903 0.444444 25.5 7.79423

428 3 207.580836 134.316872 3.1569541 0.86725657 427 0.285714 19 4.12311 0.285714 26.25 6.2998

429 3 208.830287 136.180078 3.21674629 0.79040377 428 0.545455 21.5 4.5 0.272727 29 9.62635

430 3 209.495633 138.135199 3.17735923 1.08326868 429 0.409836 30.84 11.5505 0.803279 48.0408 26.7299

431 3 211.158405 140.707633 3.13256994 0.821898 430 0.4 27.95 9.08557 0.82 47.1463 28.2675

432 3 212.354486 143.419422 3.18320028 0.821898 431 0.2 19.5 2.5 0.3 25.3333 8.34

433 3 213.389864 146.195848 3.25983367 0.821898 432 0 0 0 0.555556 20.8 3.48712

434 3 214.59484 148.898149 3.12924321 0.821898 433 0 0 0 0.416667 23.4 3.55528

435 3 215.809897 151.592741 3.31051145 0.821898 434 0.266667 26 9.61769 0.6 23 2.98142

436 3 217.151856 154.076818 3.25793607 0.821898 435 0.625 21.7 7.2118 0.25 21.75 1.92029

437 3 218.500931 156.715668 3.29377699 0.821898 436 0.444444 18.5 2.06155 0.111111 22 0

438 3 219.876691 159.340879 3.36933705 0.821898 437 0.470588 22 4.94975 0.529412 29.7778 9.63533

439 3 221.436281 161.861129 3.39353145 0.82194544 438 0.333333 19.6667 3.09121 0.555556 23.4 1.85472

440 3 223.179701 164.258035 3.38212806 0.82295947 439 0.5 27.6 4.27083 0.6 22.6667 4.85341

441 3 225.133043 166.340651 3.35838434 0.76859323 440 0.238095 29.6 5.42586 0.666667 59.3571 49.3842

442 3 227.012853 168.606504 3.00814668 0.97505804 441 0.0333333 17 0 0.833333 41.84 27.4935

443 3 229.694399 170.129921 2.60596815 1.05930555 442 0.340426 35.1875 19.9255 0.87234 59.1951 42.4851

444 3 231.888499 172.21906 2.2624729 0.7509159 443 0.431373 34.2273 17.6788 0.862745 51.6591 39.2798

445 3 233.392347 174.665185 2.27115442 0.83119031 444 0.318182 30.1429 9.73108 0.818182 37 23.2092

446 3 235.251995 176.973141 2.35096036 0.91018977 445 0.25 32.375 10.0863 0.9375 61.6667 57.0943

447 3 237.512511 178.882601 2.53932681 1.04395278 446 0.243243 26.6111 8.48619 0.878378 70.0308 50.8467

448 3 240.106293 180.306394 2.72795418 1.0496693 447 0.259259 26.4286 5.75344 0.814815 56.5909 36.7382

449 3 243.092641 180.972333 2.3976769 1.19152083 448 0.324324 23.8333 5.2731 0.837838 48.9355 35.327

450 3 245.233371 182.533109 2.40654818 1.04374523 449 0.415385 40.1111 28.1653 0.892308 85.8966 65.9375

451 3 247.961171 184.116419 2.14295968 1.04366221 450 0.261905 35.0909 16.3343 0.880952 61.2162 49.6039

452 3 250.569185 185.832561 2.18755921 1.0432649 451 0.0909091 20 0 0.727273 28.625 14.9494

453 3 252.925767 187.182822 2.71709042 0.73795292 452 0 0 0 0.428571 22 2.82843

454 3 255.022615 189.27967 2.71709042 0.62265 453 0.166667 19 0 0.166667 21 0

455 3 207.488921 138.342156 2.51554751 0.74717407 429 0.32 26.125 4.34274 0.72 40.3889 19.3481

456 3 206.948698 140.077274 2.64594821 0.74718 455 0 0 0 0.833333 24 4.60435

457 3 206.200925 141.999187 3.02131128 0.74718 456 0.0769231 17 0 0.923077 37.4167 23.4288

458 3 205.428839 143.782338 3.22447901 0.74718 457 0 0 0 0 0 0

459 3 204.763493 146.659574 3.1615202 0.74719779 458 0 0 0 0.4 22 2

460 3 204.465214 149.740209 2.97704383 0.74763661 459 0.125 16 0 0.875 27.2857 11.6339

461 3 203.84968 152.609143 2.89586213 0.7571424 460 0.357143 22.2 4.57821 0.5 24.5714 12.105

462 3 202.736026 155.283573 2.81566481 1.01619445 461 0.28 27.2857 8.13659 0.8 48.1 28.6442

463 3 202.024426 158.149542 2.8608692 0.74718 462 0.142857 24 4.32049 0.761905 51.375 31.2627

464 3 202.080168 160.658525 2.95003861 0.74718 463 0.333333 31.1667 11.3198 0.611111 43.3636 26.6774

465 3 201.085707 164.009568 2.85661146 0.74718 464 0.230769 30.6667 9.74109 1 37.7692 26.3239

466 3 199.846337 166.730845 2.777612 0.74718 465 0.125 22 0 0.375 30.3333 5.55778

467 3 198.824598 169.507271 2.66995878 0.74718 466 0 0 0 0.6 30 2.16025

468 3 198.13909 172.383914 2.598526 0.74718 467 0 0 0 1 40.1429 9.68694

469 3 197.869275 175.300881 2.37102748 0.74718 468 0.166667 17 0 0.666667 51.75 15.5302

470 3 198.070895 178.312135 1.88481492 0.74718 469 0.384615 25.4 14.9479 0.538462 32.1429 7.71693

471 3 197.037296 181.097456 1.73228939 0.74716221 470 0.454545 37.6 16.9423 0.681818 32 14.6606

472 3 194.309496 182.610199 1.84341759 0.7467649 471 0.24 34.6667 19.9639 0.68 42.4706 25.6334

473 3 191.46784 183.413714 2.01184738 0.73795885 472 0 0 0 0.777778 37.5714 20.6111

474 3 189.055516 184.985757 2.13796662 0.62265 473 0 0 0 0.714286 24.6 3.87814

475 2 278.162068 19.1483851 2.2385157 1.36915991 1 0.736573 97.3264 86.3415 0.700767 96.0839 82.5629

476 2 276.556224 16.6564212 2.1832481 0.98711373 475 0.791667 100.114 80.908 0.611111 67.5795 53.543

477 2 274.959275 14.1588238 2.13210185 0.75402915 476 0.802632 95.0164 87.5028 0.513158 65.359 40.1251

478 2 273.479147 11.5911931 2.19123581 0.8868315 477 0.75 90.3077 80.0961 0.576923 53.9 33.0317

479 2 272.079667 8.9775456 2.18100063 0.82002412 478 0.640625 93.6098 81.4727 0.484375 55.1936 34.8188

480 2 270.69264 6.3571379 2.15249512 0.760819 479 0.660714 90.6757 79.9111 0.517857 52.069 28.3706

481 2 268.68237 4.20668863 2.31479329 1.09849099 480 0.596491 88.5882 78.1093 0.561404 56.8125 33.3246

482 2 265.949826 3.1549972 2.27796799 0.94575198 481 0.65 72.8846 67.1372 0.625 51.7 29.6414

483 2 263.088601 2.38263842 2.19375606 1.07224481 482 0.759036 70.5873 60.0573 0.674699 44.1786 26.0117

484 2 260.14317 2.09573316 2.21123177 1.10608732 483 0.7875 87.746 74.3573 0.6 44.0208 25.9651

485 2 257.221459 1.60443266 2.18791501 0.94509968 484 0.710526 90.037 74.2585 0.552632 46.2619 23.3228

486 2 254.268912 1.36433289 2.17960115 0.69574911 485 0.615385 76.75 55.2607 0.711538 54.6486 31.1962

487 2 251.363805 0.78836385 2.1736415 0.62265 486 0.48 39.6667 13.7497 0.56 36.6429 10.7145

#Gage detailed SWCX file, 10 time-points, starting from 21 dpi, going upto 60 dpi. Each time-point has 7 columns, total of 70 columns.

1 3 208.783 209.275 19.3818 2 -1 1 3 208.783 209.275 19.3818 2 -1 1 3 208.783 209.275 19.3818 2 -1 1 3 208.783 209.275 19.3818 2 -1 1 3 206.872 208.671 19.5631 2 -1 1 3 207.991 209.674 20.4773 2 -1 1 3 206.824 209.475 20.4485 2 -1 1 3 207.361 209.091 20.5578 2 -1 1 3 207.361 209.091 20.5578 2 -1 1 3 207.361 209.091 20.5578 2 -1

2 3 207.452 209.198 22.7285 1 1 2 3 207.782 209.258 22.2146 1 1 2 3 208.533 209.122 23.0139 1 1 2 3 208.533 209.122 23.0139 1 1 2 3 205.993 210.005 22.7059 1 1 2 3 207.176 209.98 23.4492 1 1 2 3 206.009 209.781 23.4204 1 1 2 3 207.657 208.568 24.1651 1 1 2 3 206.926 209.415 23.9479 1 1 2 3 206.926 209.415 23.9479 1 1

3 3 206.607 210.125 27.5685 1 2 3 3 207.929 209.115 25.4847 1 2 3 3 207.929 209.115 25.4847 1 2 3 3 207.929 209.115 25.4847 1 2 3 3 206.009 209.92 25.3377 1 2 3 3 207.086 211.11 26.3848 1 2 3 3 205.919 210.911 26.356 1 2 3 3 207.193 208.988 25.9163 1 2 3 3 206.462 209.835 25.6991 1 2 3 3 206.462 209.835 25.6991 1 2

4 3 205.67 210.129 29.6973 1 3 4 3 207.298 209.699 27.6651 1 3 4 3 207.825 209.57 27.8111 1 3 4 3 207.825 209.57 27.8111 1 3 4 3 205.722 210.344 28.0575 1 3 4 3 206.441 211.613 28.8337 1 3 4 3 205.274 211.414 28.8049 1 3 4 3 207.105 209.488 27.7311 1 3 4 3 205.791 209.699 26.7814 1 3 4 3 205.855 210.576 27.0773 1 3

5 3 205.783 212.394 32.0635 1 4 5 3 206.257 212.797 30.2063 1 4 5 -2 207.608 214.278 31.8077 1 4 5 3 208.154 212.649 27.6908 1 4 5 -2 205.097 215.588 32.8787 1 4 5 3 206.741 213.396 31.2936 1 4 5 3 206.741 213.396 31.2936 1 4 5 3 208.348 215.483 32.7366 1 4 5 3 204.409 214.878 31.9633 1 4 5 3 205.421 217.266 31.5104 1 4

6 3 205.497 215.272 35.1452 1 5 6 3 205.381 215.945 34.5942 1 5 6 -2 205.847 217.963 38.384 1 5 6 3 207.294 216.548 30.9227 1 5 6 -2 202.558 217.49 38.2579 1 5 6 3 205.691 216.023 34.0652 1 5 6 3 204.42 215.532 35.3361 1 5 6 3 206.52 216.773 39.4893 1 5 6 3 202.515 216.371 38.7316 1 5 6 3 201.105 216.915 40.8632 1 5

7 3 203.157 220.529 36.7633 1 6 7 3 203.414 220.35 35.4447 1 6 7 3 202.238 220.203 40.1006 1 6 7 3 205.332 218.022 36.0832 1 6 7 3 202.002 219.86 40.1921 1 6 7 3 203.149 219.466 36.1187 1 6 7 3 203.36 217.613 37.0126 1 6 7 3 206.092 218.512 40.3996 1 6 7 3 200.13 218.04 42.482 1 6 7 3 198.492 219.292 43.5295 1 6

8 3 200.724 224.651 38.2095 1 7 8 3 201.037 224.759 35.0999 1 7 8 3 199.545 223.921 43.0931 1 7 8 3 203.185 219.742 40.6838 1 7 8 3 200.406 221.064 41.6738 1 7 8 3 201.037 222.4 37.7027 1 7 8 3 201.248 220.547 38.5966 1 7 8 3 204.518 220.512 41.5687 1 7 8 3 197.212 220.169 45.017 1 7 8 3 196.129 221.374 44.8147 1 7

0 0 X X X X X 9 -1 198.599 225.06 36.8416 0.945659 8 9 3 197.922 225.217 46.4514 0.945659 8 9 3 199.684 221.871 44.9957 0.945659 8 9 3 198.227 221.185 44.5847 0.945659 8 9 3 197.642 226.05 42.3976 0.945659 8 9 3 197.628 221.674 44.737 0.945659 8 9 3 202.454 221.334 46.4793 0.945659 8 9 3 195.14 222.034 49.4645 0.945659 8 9 3 195.14 222.034 49.4645 0.945659 8

0 0 X X X X X 10 -1 196.57 225.711 39.6185 0.945659 9 10 3 196.296 226.761 49.8234 0.945659 9 10 3 198.058 223.415 48.3677 0.945659 9 10 3 194.296 221.968 48.4889 0.945659 9 10 3 195.062 229.201 46.447 0.945659 9 10 3 195.256 223.191 49.1517 0.945659 9 10 3 199.11 223.965 51.0315 0.945659 9 10 3 191.796 224.665 54.0167 0.945659 9 10 3 191.796 224.665 54.0167 0.945659 9

0 0 X X X X X 11 -1 194.016 226.39 43.8627 0.998838 10 11 3 194.507 228.216 53.711 0.998838 10 11 3 196.269 224.87 52.2553 0.998838 10 11 3 192.718 224.979 51.8174 0.998838 10 11 3 192.757 231.214 50.3594 0.998838 10 11 3 193.79 226.428 53.092 0.998838 10 11 3 195.5 226.942 54.0497 0.998838 10 11 3 188.719 225.983 58.0885 0.998838 10 11 3 188.719 225.983 58.0885 0.998838 10

0 0 X X X X X 12 -1 191.266 227.534 47.8789 0.951064 11 12 3 192.589 228.945 57.8115 0.951064 11 12 3 196.817 227.607 55.7596 0.951064 11 12 3 191.126 226.609 56.9774 0.951064 11 12 3 190.183 233.662 54.6127 0.951064 11 12 3 191.216 228.876 57.3453 0.951064 11 12 3 192.632 229.761 57.2012 0.951064 11 12 3 187.253 227.16 62.8145 0.951064 11 12 3 186.426 227.14 62.2494 0.951064 11

0 0 X X X X X 13 -1 188.644 228.73 51.965 0.938677 12 13 3 191.591 229.777 61.2071 0.938677 12 13 3 196.183 228.921 59.9632 0.938677 12 13 3 190.805 227.906 61.8359 0.938677 12 13 3 188.38 235.538 58.3923 0.938677 12 13 3 189.733 231.334 61.9107 0.938677 12 13 3 191.406 232.283 61.6098 0.938677 12 13 3 186.027 229.682 67.2231 0.938677 12 13 3 183.545 228.519 66.5123 0.938677 12

0 0 X X X X X 14 -1 185.903 230.57 55.7206 1.18857 13 14 3 189.268 230.449 65.2468 1.18857 13 14 3 192.169 228.778 64.4332 1.18857 13 14 3 188.248 227.967 67.1201 1.18857 13 14 3 186.534 237.931 63.3139 1.18857 13 14 3 187.66 234.365 66.1366 1.18857 13 14 3 189.098 235.367 66.8813 1.18857 13 14 3 182.931 230.618 73.4218 1.18857 13 14 3 180.699 230.507 71.7916 1.18857 13

0 0 X X X X X 15 -1 183.34 233.614 58.7464 0.901229 14 15 3 187.188 232.157 69.7811 0.901229 14 15 3 187.823 232.846 69.5418 0.901229 14 15 3 184.729 228.647 71.1958 0.901229 14 15 3 184.787 240.772 66.1654 0.901229 14 15 3 185.913 237.206 68.9881 0.901229 14 15 3 188.334 237.526 71.7046 0.901229 14 15 3 179.325 232.961 77.1722 0.901229 14 15 3 178.371 232.708 76.1524 0.901229 14

0 0 X X X X X 16 -1 181.035 236.428 62.1779 0.901229 15 16 3 184.511 236.263 73.2948 0.901229 15 16 3 185.637 237.027 73.2213 0.901229 15 16 3 183.502 233.723 74.037 0.901229 15 16 3 183.765 243.417 68.796 0.901229 15 16 3 184.891 239.851 71.6187 0.901229 15 16 3 186.657 238.22 74.0181 0.901229 15 16 3 177.648 233.655 79.4857 0.901229 15 16 3 176.694 233.402 78.4659 0.901229 15

0 0 X X X X X 17 -1 178.731 239.241 65.6094 0.900678 16 17 3 183.164 238.793 75.8028 0.900678 16 17 3 184.29 239.557 75.7293 0.900678 16 17 3 181.24 237.196 75.8057 0.900678 16 17 3 182.372 244.764 71.928 0.900678 16 17 3 184.405 241.858 74.474 0.900678 16 17 3 186.171 240.227 76.8734 0.900678 16 17 3 177.162 235.662 82.341 0.900678 16 17 3 176.208 235.409 81.3212 0.900678 16

0 0 X X X X X 18 -1 176.372 242.478 68.6018 0.924164 17 18 3 181.32 241.572 77.9944 0.924164 17 18 3 182.446 242.336 77.9209 0.924164 17 18 3 179.396 239.975 77.9973 0.924164 17 18 3 180.528 247.543 74.1196 0.924164 17 18 3 182.561 244.637 76.6656 0.924164 17 18 3 185.541 243.471 79.7949 0.924164 17 18 3 174.314 238.726 86.5439 0.924164 17 18 3 173.72 238.308 85.4968 0.924164 17

0 0 X X X X X 19 -1 174.423 245.187 72.3253 0.924164 18 19 3 178.958 243.62 79.8856 0.924164 18 19 3 180.412 244.758 79.8626 0.924164 18 19 3 177.362 242.397 79.939 0.924164 18 19 3 178.238 250.638

78.0047 0.924164 18 19 3 180.271 247.732 80.5507 0.924164 18 19 3 183.352 246.714 83.4442 0.924164 18 19 3 172.168 241.567 90.3941 0.924164 18 19 3 171.574 241.149 89.347 0.924164 18

0 0 X X X X X 20 -1 172.494 248.348 75.6852 0.975076 19 20 3 177.556 246.916 81.65 0.975076 19 20 3 178.474 248.498 83.4634 0.975076 19 20 3 173.798 245.699 85.0129 0.975076 19 20 3 176.135 253.734 82.4291 0.975076 19 20 3 178.005 250.213 84.8417 0.975076 19 20 3 183.093 250.38 87.8267 0.975076 19 20 3 169.346 244.697 95.0893 0.975076 19 20 3 168.752 244.279 94.0422 0.975076 19

0 0 X X X X X 21 -1 170.588 251.35 79.2001 0.908736 20 21 3 175.65 249.918 85.1649 0.908736 20 21 3 176.568 251.5 86.9783 0.908736 20 21 3 173.454 248.089 88.0734 0.908736 20 21 3 173.404 253.843 86.5606 0.908736 20 21 3 177.802 253.002 88.3268 0.908736 20 21 3 181.388 253.592 90.5974 0.908736 20 21 3 167.421 246.628 98.2488 0.908736 20 21 3 167.421 246.628 98.2488 0.908736 20

0 0 X X X X X 22 -1 168.867 253.552 83.3462 0.670042 21 22 3 173.04 251.825 88.0903 0.670042 21 22 3 174.61 254.438 89.3238 0.670042 21 22 3 171.496 251.027 90.4189 0.670042 21 22 3 170.545 253.752 89.9598 0.670042 21 22 3 174.517 253.229 92.2559 0.670042 21 22 3 178.103 253.819 94.5265 0.670042 21 22 3 165.718 249.563 100.732 0.670042 21 22 3 165.718 249.563 100.732 0.670042 21

0 0 X X X X X 23 -1 166.305 253.871 87.6281 0.847681 22 23 3 170.578 254.393 91.2249 0.847681 22 23 3 170.913 253.864 93.1245 0.847681 22 23 3 170.557 253.963 92.493 0.847681 22 23 3 169.083 254.381 92.7976 0.847681 22 23 3 172.041 253.881 95.7532 0.847681 22 23 3 175.956 253.932 98.4557 0.847681 22 23 3 164.891 251.005 102.686 0.847681 22 23 3 164.891 251.005 102.686 0.847681 22

0 0 X X X X X 24 -1 164.186 253.932 92.1564 0.847681 23 24 3 167.638 254.309 94.9034 0.847681 23 24 3 167.973 253.78 96.803 0.847681 23 24 3 167.617 253.879 96.1715 0.847681 23 24 3 167.617 253.879 96.1715 0.847681 23 24 3 170.398 253.676 98.9838 0.847681 23 24 3 174.313 253.727 101.686 0.847681 23 24 3 164.379 253.418 104.139 0.847681 23 24 3 164.379 253.418 104.139 0.847681 23

0 0 X X X X X 25 -1 162.067 253.992 96.6848 0.805555 24 25 3 163.998 254.359 98.7845 0.805555 24 25 3 165.35 253.973 99.9561 0.805555 24 25 3 164.994 254.072 99.3246 0.805555 24 25 3 164.994 254.072 99.3246 0.805555 24 25 3 167.775 253.869 102.137 0.805555 24 25 3 172.054 253.783 105.018 0.805555 24 25 3 162.12 253.474 107.471 0.805555 24 25 3 162.12 253.474 107.471 0.805555 24

0 0 X X X X X 26 -1 159.794 254 101.139 0.805555 25 26 3 162.048 253.912 102.294 0.805555 25 26 3 163.4 253.526 103.466 0.805555 25 26 3 162.546 253.831 102.728 0.805555 25 26 3 162.546 253.831 102.728 0.805555 25 26 3 165.327 253.628 105.54 0.805555 25 26 3 170.909 254.072 109.299 0.805555 25 26 3 158.887 253.572 112.9 0.805555 25 26 3 158.887 253.572 112.9 0.805555 25

0 0 X X X X X 27 -1 157.522 254.007 105.592 0.778316 26 27 3 159.776 253.919 106.747 0.778316 26 27 3 161.128 253.533 107.919 0.778316 26 27 3 160.274 253.838 107.181 0.778316 26 27 3 160.274 253.838 107.181 0.778316 26 27 3 161.789 253.961 109.007 0.778316 26 27 3 167.443 254.469 113.534 0.778316 26 27 3 156.548 254.062 117.303 0.778316 26 27 3 156.548 254.062 117.303 0.778316 26

0 0 X X X X X 28 -1 154.538 254.018 109.605 0.768941 27 28 3 157.425 253.785 111.267 0.768941 27 28 3 158.777 253.399 112.439 0.768941 27 28 3 157.923 253.704 111.701 0.768941 27 28 3 157.923 253.704 111.701 0.768941 27 28 3 159.438 253.827 113.527 0.768941 27 28 3 166.966 253.764 118.655 0.768941 27 28 3 154.817 253.298 122.402 0.768941 27 28 3 154.229 254.04 122.263 0.768941 27

0 0 X X X X X 29 -1 151.686 254.016 113.711 0.768941 28 29 3 154.715 253.613 116.598 0.768941 28 29 3 156.067 253.227 117.77 0.768941 28 29 3 153.411 253.496 117.171 0.768941 28 29 3 154.837 253.169 117.931 0.768941 28 29 3 156.783 253.592 119.359 0.768941 28 29 3 162.871 254.381 123.847 0.768941 28 29 3 152.346 253.366 127.54 0.768941 28 29 3 151.758 254.108 127.401 0.768941 28

0 0 X X X X X 30 -1 148.834 254.014 117.818 0.764894 29 30 3 152.794 253.889 121.01 0.764894 29 30 3 154.296 253.432 122.478 0.764894 29 30 3 151.443 253.902 120.457 0.764894 29 30 3 152.869 253.575 121.217 0.764894 29 30 3 155.686 254.011 122.968 0.764894 29 30 3 160.928 254.025 126.884 0.764894 29 30 3 151.541 253.593 131.222 0.764894 29 30 3 149.981 253.976 131.146 0.764894 29

0 0 X X X X X 31 -1 146.529 254.013 122.255 0.615107 30 31 3 151.382 253.785 125.068 0.615107 30 31 3 151.382 253.785 125.068 0.615107 30 31 3 149.339 253.984 124.67 0.615107 30 31 3 151.354 253.817 125.687 0.615107 30 31 3 154.171 254.253 127.438 0.615107 30 31 3 158.745 254.011 130.848 0.615107 30 31 3 151.439 253.668 134.296 0.615107 30 31 3 148.727 253.576 133.497 0.615107 30

0 0 X X X X X 32 -1 144.296 254.012 126.729 0.657157 31 32 3 149.149 253.784 129.542 0.657157 31 32 3 149.149 253.784 129.542 0.657157 31 32 3 147.662 254.029 128.489 0.657157 31 32 3 150.246 253.611 130.024 0.657157 31 32 3 153.063 254.047 131.775 0.657157 31 32 3 156.454 253.794 135.089 0.657157 31 32 3 149.148 253.451 138.537 0.657157 31 32 3 147.115 253.687 138.454 0.657157 31

0 0 X X X X X 33 -1 142.232 254.01 131.283 0.728286 32 33 3 147.085 253.782 134.096 0.728286 32 33 3 147.085 253.782 134.096 0.728286 32 33 3 145.598 254.027 133.043 0.728286 32 33 3 148.182 253.609 134.578 0.728286 32 33 3 150.933 253.821 136.873 0.728286 32 33 3 154.519 254.411 139.144 0.728286 32 33 3 148.208 254.196 143.366 0.728286 32 33 3 144.975 253.817 142.286 0.728286 32

0 0 X X X X X 34 -1 140.53 254.009 135.984 0.722147 33 34 3 144.649 253.617 137.336 0.722147 33 34 3 144.649 253.617 137.336 0.722147 33 34 3 144.045 253.991 136.711 0.722147 33 34 3 147.371 253.417 138.619 0.722147 33 34 3 149.732 253.534 139.864 0.722147 33 34 3 150.713 254.57 142.415 0.722147 33 34 3 147.11 254.697 148.669 0.722147 33 34 3 141.232 254.374 147.045 0.722147 33

0 0 X X X X X 35 -1 139.442 254.009 140.865 0.716589 34 35 3 143.119 253.812 141.772 0.716589 34 35 3 143.119 253.812 141.772 0.716589 34 35 3 141.946 253.656 141.143 0.716589 34 35 3 146.208 253.624 143.877 0.716589 34 35 3 148.569 253.741 145.122 0.716589 34 35 3 146.695 254.842 145.199 0.716589 34 35 3 144.057 254.174 155.366 0.716589 34 35 3 138.632 254.552 153.702 0.716589 34

0 0 X X X X X 36 -1 137.741 254.008 145.566 0.756119 35 36 3 141.162 253.153 146.169 0.756119 35 36 3 141.162 253.153 146.169 0.756119 35 36 3 139.237 254.054 144.648 0.756119 35 36 3 145.276 253.609 148.651 0.756119 35 36 3 147.637 253.726 149.896 0.756119 35 36 3 143.023 254.334 149.776 0.756119 35 36 3 142.354 254.499 160.634 0.756119 35 36 3 136.602 254.009 158.906 0.756119 35

0 0 X X X X X 37 -1 136.977 254.007 150.507 0.606626 36 37 3 139.009 253.51 150.999 0.606626 36 37 3 139.009 253.51 150.999 0.606626 36 37 3 135.968 253.781 148.657 0.606626 36 37 3 143.186 253.5 154.759 0.606626 36 37 3 145.547 253.617 156.004 0.606626 36 37 3 140.933 254.225 155.884 0.606626 36 37 3 140.044 254.415 166.627 0.606626 36 37 3 134.292 253.925 164.899 0.606626 36

0 0 X X X X X 38 -1 135.912 254.007 155.393 0.606626 37 38 3 137.293 253.63 155.244 0.606626 37 38 3 138.207 253.118 156.368 0.606626 37 38 3 134.866 253.734 155.646 0.606626 37 38 3 141.458 253.704

157.732 0.606626 37 38 3 145.282 254.298 159.037 0.606626 37 38 3 140.145 254.165 158.839 0.606626 37 38 3 139.256 254.355 169.582 0.606626 37 38 3 132.672 254.049 167.371 0.606626 37

0 0 X X X X X 39 -1 134.378 254.006 162.428 0.606626 38 39 3 135.916 253.354 159.419 0.606626 38 39 3 137.8 253.984 163.238 0.606626 38 39 3 133.669 253.814 161.984 0.606626 38 39 3 141.065 253.828 162.486 0.606626 38 39 3 144.691 253.652 163.161 0.606626 38 39 3 139.025 253.944 164.232 0.606626 38 39 3 137.061 253.515 175.201 0.606626 38 39 3 129.251 253.641 172.467 0.606626 38

0 0 X X X X X 0 0 X X X X X 0 0 X X X X X 0 0 X X X X X 40 -1 134 254 165.75 1 39 40 3 139.111 253.29 164.937 1 39 40 3 143.769 253.925 166.742 1 39 40 3 138.967 254.32 168.258 1 39 40 3 134.058 253.34 179.422 1 39 40 3 125.852 254.075 176.661 1 39

0 0 X X X X X 0 0 X X X X X 0 0 X X X X X 0 0 X X X X X 41 -1 133 254 169.625 1 40 41 3 138.352 253.303 168.337 1 40 41 3 143.01 253.938 170.142 1 40 41 3 138.208 254.333 171.658 1 40 41 3 130.902 253.99 175.106 0.5 40 41 3 121.457 254.169 182.581 1 40

0 0 X X X X X 0 0 X X X X X 0 0 X X X X X 0 0 X X X X X 42 -1 133 254 172.5 0.5 41 42 3 136.858 253.51 171.456 0.5 41 42 3 142.206 253.651 174.36 0.5 41 42 3 136.087 254.487 174.551 0.5 41 42 3 130.269 254.237 170.895 0.5 41 42 3 117.638 253.993 186.017 1 41

0 0 X X X X X 0 0 X X X X X 0 0 X X X X X 0 0 X X X X X 0 0 X X X X X 0 0 X X X X X 43 -1 140.201 254.062 177.403 0.5 42 43 3 132.039 254.135 177.656 0.5 42 43 3 129.218 253.754 167.721 0.5 42 43 3 113.725 254.233 190.195 1 42

0 0 X X X X X 0 0 X X X X X 0 0 X X X X X 0 0 X X X X X 0 0 X X X X X 0 0 X X X X X 44 -1 135.451 254.062 181.861 0.5 43 44 3 130.85 254.101 181.702 0.5 43 44 3 126.837 254.24 162.441 0.5 43 44 3 113.089 254.207 195.135 1 43

9 3 198.292 228.94 39.0395 1 8 40 3 198.801 228.948 36.4274 1 8 40 3 197.561 228.456 42.8023 1 8 40 3 201.085 225.926 41.5829 1 8 43 3 198.42 225.359 41.8414 1 8 43 3 199.66 226.749 38.1368 1 8 45 3 199.712 225.342 40.1162 1 8 45 3 202.438 226.919 41.5392 1 8 45 3 195.885 226.44 46.218 1 8 45 3 195.885 226.44 46.218 1 8

10 3 195.898 233.162 40.2411 1 9 41 3 196.346 233.103 37.8273 1 40 41 3 196.373 232.912 43.0127 1 40 41 3 199.099 232.358 43.7467 1 40 44 3 196.787 230.341 42.9023 1 43 44 3 198.027 231.731 39.1977 1 43 46 3 198.079 230.324 41.1771 1 45 46 3 200.805 231.901 42.6001 1 45 46 3 194.252 231.422 47.2789 1 45 46 3 194.252 231.422 47.2789 1 45

11 3 193.504 237.384 41.4427 1 10 42 3 193.843 236.874 39.5849 1 41 42 3 195.143 235.977 43.7416 1 41 42 3 197.421 235.996 44.777 1 41 45 3 193.356 233.959 45.0902 1 44 45 3 195.245 236.753 40.2562 1 44 47 3 195.71 236.88 43.0208 1 46 47 3 198.436 238.457 44.4438 1 46 47 3 193.6 235.891 48.2343 1 46 47 3 192.338 236.976 49.07 1 46

12 3 191.206 241.614 42.7923 1 11 43 3 191.798 239.246 41.8344 1 42 43 3 193.456 240.791 44.2409 1 42 43 3 195.601 240.212 47.2052 1 42 46 3 188.415 238.471 49.5169 1 45 46 3 194.335 242.935 41.572 1 45 48 3 195.328 242.837 48.0707 1 47 48 3 196.056 244.238 47.5371 1 47 48 3 192.574 240.95 50.4256 1 47 48 3 188.455 243.707 56.747 1 47

13 3 188.68 245.499 44.6697 1 12 44 3 190.26 241.913 43.0012 1 43 44 3 191.943 246.384 47.0638 1 43 44 3 194.432 243.438 50.5611 1 43 47 3 188.134 245.674 52.1068 1 46 47 3 192.878 248.381 44.1357 1 46 49 3 193.712 248.44 54.786 1 48 49 3 194.406 249.902 49.8168 1 48 49 3 190.837 246.694 53.6323 1 48 49 3 184.991 250.764 62.9609 1 48

14 3 186.018 249.078 46.9301 1 13 45 3 186.346 245.421 44.7423 1 44 45 3 188.811 248.341 48.9473 1 44 45 3 189.761 248.281 51.7766 1 44 48 3 184.71 247.411 53.0465 1 47 48 3 189.216 252.153 47.3489 1 47 50 3 190.815 251.411 55.2678 1 49 50 3 189.68 252.718 52.8632 1 49 50 3 182.813 252.375 56.8307 1 49 50 3 178.518 254.108 66.4015 1 49

15 3 182.785 252.489 48.6369 1 14 46 3 182.462 249.566 47.0595 1 45 46 3 184.75 251.368 50.3888 1 45 46 3 185.7 251.308 53.2181 1 45 49 3 182.096 249.548 53.5589 1 48 49 3 183.847 253.212 51.4587 1 48 51 3 187.485 253.917 58.4847 1 50 51 3 185.448 254.496 55.0637 1 50 51 3 178.581 254.153 59.0312 1 50 51 3 174.318 254.186 68.467 1 50

16 3 178.897 253.5 51.6143 1 15 47 3 178.298 253.851 50.0073 1 46 47 3 181.566 253.982 51.4686 1 46 47 3 181.649 252.952 55.176 1 46 50 3 178.583 252.812 54.3308 1 49 50 3 179.296 254.036 54.6346 1 49 52 3 182.934 254.741 61.6606 1 51 52 3 180.708 254.055 58.9266 1 51 52 3 173.402 253.712 62.3749 1 51 52 3 169.588 253.806 72.6827 1 51

17 3 174.912 253.878 54.6098 1 16 48 3 173.02 253.345 52.7457 1 47 48 3 176.061 253.998 54.506 1 47 48 3 178.996 252.993 59.1367 1 47 51 3 174.147 254.343 57.0905 1 50 51 3 175.763 253.59 58.2264 1 50 53 3 178.943 254.694 64.0714 1 52 53 3 178.91 254.429 64.0488 1 52 53 3 171.604 254.086 67.4971 1 52 53 3 167.79 254.18 77.8049 1 52

18 3 170.845 253.76 57.5157 1 17 49 3 168.953 253.227 55.6516 1 48 49 3 173.53 253.734 58.8607 1 48 49 3 176.041 252.506 61.9795 1 48 52 3 171.45 253.577 58.8558 1 51 52 3 173.066 252.824 59.9917 1 51 54 3 175.678 254.542 70.6072 1 53 54 3 176.453 254.003 70.969 1 53 54 3 169.147 253.66 74.4173 1 53 54 3 165.049 254.054 84.8555 1 53

19 3 166.884 254.08 60.5506 1 18 50 3 165.338 253.617 58.8205 1 49 50 3 171.4 253.905 63.8325 1 49 50 3 172.871 254.671 65.4123 1 49 53 3 168.563 253.683 62.5638 1 52 53 3 170.179 252.93 63.6997 1 52 55 3 173.481 253.953 75.1621 1 54 55 3 174.852 253.24 76.8111 1 54 55 3 166.057 253.945 80.1822 1 54 55 3 161.7 253.845 90.2279 1 54

20 3 163.424 254.021 64.1599 1 19 51 3 161.291 253.973 63.4603 1 50 51 3 168.937 253.467 68.5943 1 50 51 3 169.96 253.837 69.4317 1 50 54 3 166.972 254.066 67.8994 1 53 54 3 168.422 253.401 68.6239 1 53 56 3 171.724 254.424 80.0863 1 55 56 3 174.129 252.949 82.0402 1 55 56 3 161.845 254.326 84.5011 1 55 56 3 159.566 253.262 95.5005 1 55

21 3 160.75 253.976 68.3846 1 20 52 3 157.264 254.152 67.364 1 51 52 3 168.01 253.852 72.8 1 51 52 3 168.01 253.852 72.8 1 51 55 3 165.036 254.255 72.1358 1 54 55 3 166.486 253.59 72.8603 1 54 57 3 169.788 254.613 84.3227 1 56 57 3 172.89 253.097 86.4949 1 56 57 3 160.606 254.481 88.6932 1 56 57 3 158.788 252.797 98.9357 1 56

22 3 158.629 253.982 72.9125 1 21 53 3 154.298 254.381 71.127 1 52 53 3 165.044 254.081 76.563 1 52 53 3 165.044 254.081 76.563 1 52 56 3 163.058 254.353 75.8406 1 55 56 3 164.508 253.688 76.5651 1

55 58 3 167.81 254.711 88.0275 1 57 58 3 171.908 253.203 91.6355 1 57 58 3 159.624 254.587 93.8338 1 57 58 3 156.079 253.482 102.3 1 57

23 3 156.321 253.989 77.3479 1 22 54 3 151.451 253.643 75.2209 1 53 54 3 162.197 253.343 80.6569 1 53 54 3 163.1 253.899 81.4953 1 53 57 3 161.114 254.171 80.7729 1 56 57 3 162.564 253.506 81.4974 1 56 59 3 165.329 253.486 92.4595 1 58 59 3 169.634 253.827 95.7089 1 58 59 3 159.11 254.611 98.9599 1 58 59 3 154.392 253.435 106.241 1 58

24 3 153.869 253.997 81.7053 1 23 55 3 148.999 253.651 79.5783 1 54 55 3 158.861 253.924 85.4328 1 54 55 3 160.666 255.037 87.1095 1 54 58 3 158.68 255.309 86.3871 1 57 58 3 158.771 253.923 86.636 1 57 60 3 162.298 253.741 98.2585 1 59 60 3 166.603 254.082 101.508 1 59 60 3 157.171 255.237 104.846 1 59 60 3 151.943 254.193 110.923 1 59

25 3 151.034 253.807 86.3075 1 24 56 3 145.193 253.648 83.7703 1 55 56 3 155.453 253.778 90.6735 1 55 56 3 159.331 254.177 90.8143 1 55 59 3 157.263 254.263 89.8213 1 58 59 3 157.263 254.263 89.8213 1 58 61 3 160.79 254.081 101.444 1 60 61 3 164.421 252.886 104.33 1 60 61 3 154.989 254.041 107.668 1 60 61 3 149.172 254.003 114.948 1 60

26 3 147.734 254.172 88.9406 1 25 57 3 142.515 253.867 87.8001 1 56 57 3 152.333 253.339 94.7179 1 56 57 3 157.177 254.101 94.3642 1 56 60 3 155.109 254.187 93.3712 1 59 60 3 155.109 254.187 93.3712 1 59 62 3 158.636 254.005 104.994 1 61 62 3 162.267 252.81 107.88 1 61 62 3 152.835 253.965 111.218 1 61 62 3 148.55 254.034 118.07 1 61

27 3 144.808 253.949 90.6904 1 26 58 3 140.841 254.536 90.1059 1 57 58 3 148.546 253.691 99.2863 1 57 58 3 153.429 254.051 98.7495 1 57 61 3 151.361 254.137 97.7565 1 60 61 3 152.131 254.101 98.3085 1 60 63 3 155.608 253.941 109.73 1 62 63 3 159.239 252.746 112.616 1 62 63 3 151.731 253.712 116.388 1 62 63 3 147.334 254.033 122.037 1 62

0 0 X X X X X 59 -1 138.108 253.655 93.9044 1 58 59 -4 145.662 253.177 102.604 1 58 59 0 X X X X X 62 -5 146.006 253.292 100.993 1 61 62 3 149.7 252.955 101.511 1 61 64 3 153.384 254.464 112.935 1 63 64 3 157.624 252.994 116.106 1 63 64 3 150.721 252.753 119.602 1 63 64 3 146.054 253.774 125.182 1 63

0 0 X X X X X 60 -1 135.75 254 96.6667 1 59 60 -4 143.304 253.522 105.366 1 59 60 0 X X X X X 63 -5 143.648 253.637 103.755 1 62 63 3 147.683 253.236 103.636 1 62 65 3 151.367 254.745 115.06 1 64 65 3 155.607 253.275 118.231 1 64 65 3 150.814 253.386 123.445 1 64 65 3 144.832 253.487 129.528 1 64

0 0 X X X X X 61 -1 134 254 99.5 1 60 61 -4 141.554 253.522 108.199 1 60 61 0 X X X X X 64 -5 141.898 253.637 106.588 1 63 64 3 145.933 253.236 106.469 1 63 66 3 149.617 254.745 117.893 1 65 66 3 153.857 253.275 121.064 1 65 66 3 149.992 253.46 127.426 1 65 66 3 142.654 253.917 132.681 1 65

0 0 X X X X X 62 -1 131.349 254.21 103.537 1 61 62 -4 138.903 253.732 112.236 1 61 62 0 X X X X X 65 -5 139.247 253.847 110.625 1 64 65 -4 143.282 253.446 110.506 1 64 67 -5 148.341 254.652 120.943 1 66 67 3 152.581 253.182 124.114 1 66 67 3 148.716 253.367 130.476 1 66 67 3 141.378 253.824 135.731 1 66

0 0 X X X X X 63 -1 128.809 253.927 107.735 1 62 63 -4 136.363 253.449 116.434 1 62 63 0 X X X X X 66 -5 136.609 253.959 116.575 1 65 66 -4 140.644 253.558 116.456 1 65 68 -5 147.499 254.346 124.227 1 67 68 3 150.938 252.916 127.214 1 67 68 3 147.073 253.101 133.576 1 67 68 3 139.863 253.832 138.662 1 67

0 0 X X X X X 0 0 X X X X X 0 0 X X X X X 0 0 X X X X X 0 0 X X X X X 0 0 X X X X X 0 0 X X X X X 69 -1 148 254 130.583 1 68 69 3 146.362 254.32 136.246 1 68 69 3 138.709 254.083 141.42 1 68

0 0 X X X X X 0 0 X X X X X 0 0 X X X X X 0 0 X X X X X 0 0 X X X X X 0 0 X X X X X 0 0 X X X X X 70 -1 145 254 135.583 1 69 70 3 145.086 254.482 141.259 1 69 70 3 136.295 254.033 145.561 1 69

0 0 X X X X X 0 0 X X X X X 0 0 X X X X X 0 0 X X X X X 0 0 X X X X X 0 0 X X X X X 0 0 X X X X X 71 -1 143.5 254 139.75 1.5 70 71 3 144.46 254.605 146.107 1.5 70 71 3 134.273 254.327 151.897 1 70

0 0 X X X X X 0 0 X X X X X 0 0 X X X X X 0 0 X X X X X 0 0 X X X X X 0 0 X X X X X 0 0 X X X X X 72 -1 141 254 145.5 1 71 72 -4 142.361 253.755 150.134 1 71 72 -5 132.9 253.627 157.662 1 71

0 0 X X X X X 0 0 X X X X X 0 0 X X X X X 0 0 X X X X X 0 0 X X X X X 0 0 X X X X X 0 0 X X X X X 73 -1 137.394 254.042 149.149 1.41667 72 73 -4 138.755 253.797 153.783 1.41667 72 73 0 X X X X X

0 0 X X X X X 0 0 X X X X X 0 0 X X X X X 0 0 X X X X X 0 0 X X X X X 0 0 X X X X X 0 0 X X X X X 74 -1 127.25 254 153.229 1.1 73 74 -4 128.611 253.755 157.863 1.1 73 74 0 X X X X X

0 0 X X X X X 0 0 X X X X X 0 0 X X X X X 0 0 X X X X X 0 0 X X X X X 0 0 X X X X X 0 0 X X X X X 75 -1 119 254 155 1 74 75 -4 120.361 253.755 159.634 1 74 75 0 X X X X X

28 3 191 247 45.5 1 13 64 3 191.2 245.799 43.7618 1 44 64 3 193.024 249.869 48.2836 1 44 64 3 196.116 246.164 51.0756 1 44 67 3 189.69 248.808 52.8696 1 47 67 3 194.462 251.47 45.9919 1 47 69 3 195.496 251.655 56.4457 1 49 76 3 195.364 253.203 52.1961 1 49 76 3 192.123 250.322 55.1495 1 49 76 3 187.626 252.435 64.6647 1 49

29 3 192 250 46.25 1 28 65 3 191.525 248.746 45.6112 1 64 65 3 194.388 252.204 50.4064 1 64 65 3 197.218 249.996 52.5418 1 64 68 3 190.77 252.068 54.0627 1 67 68 3 194.499 253.639 46.991 1 67 70 3 195.119 253.234 57.8874 1 69 77 3 199.415 253.962 55.0642 1 76 77 3 193.188 252.979 56.7519 1 76 77 3 188.355 254.736 66.9338 1 76

30 3 193.134 253.421 47.2027 1 29 66 3 191.905 251.614 47.2215 1 65 66 3 194.653 253.668 52.7552 1 65 66 3 199.441 252.159 53.0778 1 65 69 3 193.437 253.916 55.748 1 68 69 3 197.356 253.518 49.3133 1 68 71 3 197.387 254.031 61.1015 1 70 78 3 202.179 253.913 58.5214 1 77 78 3 196.081 253.681 59.8356 1 77 78 3 191.123 253.882 69.3154 1 77

31 3 196 254 49 1 30 67 3 194.185 253.481 49.7411 1 66 67 3 196.473 253.728 54.5325 1 66 67 3 201.505 254.156 54.1774 1 66 70 3 195.884 253.496 59.3874 1 69 70 3 197.697 253.753 54.3692 1 69 72 3 198.731 253.938 64.823 1 71 79 3 203.886 253.803 63.358 1 78 79 3 198.105 253.894 63.6308 1 78 79 3 193.712 254.159 72.8771 1 78

32 3 197.425 253.811 49.4382 1 31 68 3 195.214 254.097 53.669 1 67 68 3 197.502 254.344 58.4604 1 67 68 3 202.534 254.772 58.1053 1 67 71 3 196.823 253.133 62.1467 1 70 71 3 197.863 254.084 58.0177 1 70 73 3 201.058 254.112 69.2708 1 72 80 3 203.621 254.187 68.6117 1 79 80 3 198.139 254.142 68.6723 1 79 80 3 194.592 254.516 78.2806 1 79

33 3 196.322 254.004 57.3791 1 32 69 3 196.322 254.004 57.3791 1 68 69 3 198.611 254.383 61.0593 1 68 69 3 203.643 254.811 60.7042 1 68 72 3 196.653 252.913 65.0302 1 71 72 3 197.498 253.804 61.7206 1 71 74 3 203.549 253.516 73.3096 1 73 81 3 206.235 253.707 73.4249 1 80 81 3 197.159 253.482 75.5362 1 80 81 3 192.845 254.612 85.2114 1 80

34 3 194.928 253.91 62.1798 1 33 70 3 193.503 253.953 61.2937 1 69 70 3 198.367 254.212 63.9275 1 69 70 3 203.399 254.64 63.5724 1 69 73 3 195.028 253.115 67.8874 1 72 73 3 197.84 254.319 67.1809 1 72 75 3 203.891 254.031 78.7699 1 74 82 3 206.029 254.183 78.9225 1 81 82 3 195.824 253.618 81.6117 1 81 82 3 190.663 254.644 91.2098 1 81

35 3 193.533 253.815 66.9805 1 34 71 3 191.728 253.646 64.8497 1 70 71 3 197.455 254.029 67.1341 1 70 71 3 202.487 254.457 66.779 1 70 74 3 193.634 253.42 71.4587 1 73 74 3 196.897 254 72.2324 1 73 76 3 202.948 253.712 83.8214 1 75 83 3 205.771 253.87 83.2799 1 82 83 3 194.538 253.091 85.8001 1 82 83 3 189.907 254.462 95.2952 1 82

36 3 192.419 254.004 71.8511 1 35 72 3 190.317 253.433 69.8566 1 71 72 3 197.457 253.837 70.5865 1 71 72 3 202.489 254.265 70.2314 1 71 75 3 193.825 253.971 74.892 1 74 75 3 197.088 254.551 75.6657 1 74 77 3 203.139 254.263 87.2547 1 76 84 3 205.076 254.045 87.8408 1 83 84 3 193.266 253.963 89.9906 1 83 84 3 189.15 254.519 99.8528 1 83

37 3 191.304 254.192 76.7216 1 36 73 3 189.057 253.299 76.2582 1 72 73 3 197.133 254.347 75.5255 1 72 73 3 201.506 253.845 75.3689 1 72 76 3 193.989 253.811 80.2883 1 75 76 3 196.961 254.074 82.1013 1 75 78 3 203.012 253.786 93.6903 1 77 85 3 204.06 253.816 92.8251 1 84 85 3 193.212 253.047 95.2377 1 84 85 3 188.016 254.349 104.681 1 84

38 3 190.172 254.278 81.5911 1 37 74 -4 188.326 254.013 82.5499 1 73 74 0 X X X X X 74 -5 200.356 254.076 79.163 1 73 77 3 194.08 253.749 84.054 1 76 77 3 196.91 254.599 85.5609 1 76 79 3 202.961 254.311 97.1499 1 78 86 3 204.406 254.347 96.9412 1 85 86 3 192.201 253.64 100.65 1 85 86 3 187.211 254.192 110.204 1 85

39 3 189.348 253.638 87.09 1 38 75 -4 189.348 253.638 87.09 1 74 75 0 X X X X X 75 -5 199.684 253.65 83.6057 1 74 78 3 193.408 253.323 88.4967 1 77 78 3 196.669 254.467 90.1709 1 77 80 3 202.618 253.992 101.099 1 79 87 3 205.058 254.067 101.409 1 86 87 3 191.679 254.01 104.623 1 86 87 3 186.689 254.562 114.177 1 86

40 3 188.631 254.262 90.2723 1 39 76 -4 188.631 254.262 90.2723 1 75 76 0 X X X X X 76 -5 199.128 254.368 87.4779 1 75 79 3 193.615 253.536 91.8945 1 78 79 3 196.876 254.68 93.5687 1 78 81 3 205.972 254.004 105.544 1 80 88 3 205.972 254.004 105.544 1 87 88 3 190.86 253.514 108.22 1 87 88 3 187.323 254.557 117.087 1 87

0 0 X X X X X 0 0 X X X X X 0 0 X X X X X 77 -1 198.5 254 91.1667 1 76 80 3 193.738 253.248 96.0411 1 79 80 -4 196.817 254.366 97.3828 1 79 82 0 X X X X X 89 -5 206.422 253.229 108.774 1 88 89 3 190.403 253.378 111.17 1 88 89 3 187.687 254.547 120.412 1 88

0 0 X X X X X 0 0 X X X X X 0 0 X X X X X 78 -1 197 254 94 1 77 81 3 192.754 252.818 99.5987 1 80 81 -4 196.932 254.453 100.86 1 80 83 0 X X X X X 90 -5 206.852 254.023 112.016 1 89 90 3 190.648 253.439 114.595 1 89 90 3 187.932 254.608 123.837 1 89

0 0 X X X X X 0 0 X X X X X 0 0 X X X X X 79 -1 197 254 97 1 78 82 3 191.628 253.51 103.218 1 81 82 -4 195.417 254.885 104.869 1 81 84 0 X X X X X 91 -5 206.639 253.51 116.191 1 90 91 3 191.77 253.088 117.231 1 90 91 3 187.917 253.949 126.629 1 90

0 0 X X X X X 0 0 X X X X X 0 0 X X X X X 80 -1 197.691 254.217 99.9161 1 79 83 3 191.675 254.557 106.992 1 82 83 -4 194.754 255.675 108.334 1 82 85 0 X X X X X 92 -5 206.74 253.794 119.981 1 91 92 3 192.978 253.245 120.861 1 91 92 3 187.988 253.797 130.415 1 91

0 0 X X X X X 0 0 X X X X X 0 0 X X X X X 0 0 X X X X X 0 0 X X X X X 0 0 X X X X X 0 0 X X X X X 93 -1 208 254 122 1 92 93 3 193.347 253.92 123.486 1 92 93 3 188.181 253.866 133.028 1 92

0 0 X X X X X 0 0 X X X X X 0 0 X X X X X 0 0 X X X X X 0 0 X X X X X 0 0 X X X X X 0 0 X X X X X 94 -1 209.692 254.374 125.617 1.25 93 94 -4 193.669 253.775 126.896 1.25 93 94 -5 188.627 253.742 136.866 1.25 93

0 0 X X X X X 0 0 X X X X X 0 0 X X X X X 0 0 X X X X X 0 0 X X X X X 0 0 X X X X X 0 0 X X X X X 95 -1 211.311 254.301 129.028 1 94 95 -4 193.866 253.337 130.129 1 94 95 -5 189.345 254.051 141.215 1 94

41 3 205.25 215 37.5 1 6 77 3 205.25 215 37.5 1 6 77 3 204.858 217.111 40.8945 1 6 81 3 205.929 215.129 36.4159 1 6 84 3 203.331 216.161 40.4059 1 6 84 3 204.393 215.961 39.1359 1 6 86 3 203.947 214.856 38.7292 1 6 96 3 205.53 215.481 42.2823 1 6 96 3 200.573 214.058 41.9573 1 6 96 3 199.198 215.242 43.3571 1 6

42 3 203.639 214.978 41.7554 1 41 78 3 203.002 213.347 41.49 1 77 78 3 202.791 216.275 44.7331 1 77 82 3 204.203 214.422 41.3788 1 81 85 3 202.414 215.166 43.162 1 84 85 3 202.746 218.112 43.2892 1 84 87 3 201.815 214.608 43.0902 1 86 97 3 201.872 215.423 45.2121 1 96 97 3 199.316 212.758 45.7731 1 96 97 3 197.408 213.161 45.737 1 96

43 3 202.132 214.953 46.523 1 42 79 3 201.495 213.322 46.2576 1 78 79 3 201.359 216.237 47.9851 1 78 83 3 202.771 214.384 44.6308 1 82 86 3 200.706 213.417 46.5133 1 85 86 3 201.253 217.831 47.2047 1 85 88 3 200.267 214.784 46.9658 1 87 98 3 200.267 214.784 46.9658 1 97 98 3 197.622 211.764 47.8415 1 97 98 3 195.87 212.234 47.9807 1 97

44 3 200.99 214.947 51.3908 1 43 80 3 200.294 213.524 51.4058 1 79 80 3 200.158 216.439 53.1333 1 79 84 3 201.134 213.782 49.8098 1 83 87 3 199.097 213.011 50.1286 1 86 87 3 200.331 217.213 51.1645 1 86 89 3 199.233 215.081 50.8458 1 88 99 3 199.233 215.081 50.8458 1 98 99 3 196.557 211.339 51.3141 1 98 99 3 194.795 211.841 51.1097 1 98

45 3 200.025 214.961 56.2968 1 44 81 3 199.329 213.538 56.3118 1 80 81 3 197.962 216.324 57.1489 1 80 85 3 200.582 212.851 56.0114 1 84 88 3 197.443 213.322 53.9089 1 87 88 3 200.026 216.171 56.1999 1 87 90 3 199.359 214.714 56.2263 1 89 100 3 199.359 214.714 56.2263 1 99 100 3 195.259 210.894 56.7209 1 99 100 3 193.563 212.058 56.1885 1 99

46 3 199.319 214.876 61.2459 1 45 82 3 199.319 214.876 61.2459 1 81 82 3 196.769 217.348 61.7526 1 81 86 3 202.088 214.056 62.1204 1 85 89 3 195.873 213.756 60.244 1 88 89 3 198.287 215.517 63.3752 1 88 91 3 198.97 215.168 63.597 1 90 101 3 198.97 215.168 63.597 1 100 101 3 194.26 209.33 63.4012 1 100 101 3 192.468 209.935 62.5657 1 100

47 3 198.844 214.325 66.2928 1 46 83 3 198.35 214.754 66.4814 1 82 83 3 196.983 217.54 67.3185 1 82 87 3 203.133 215.842 66.7111 1 86 90 3 194.627 215.1 64.7362 1 89 90 3 198.077 215.258 67.6103 1 89 92 3 198.76 214.909 67.8321 1 91 102 3 198.76 214.909 67.8321 1 101 102 3 194.098 208.356 68.5733 1 101 102 3 191.668 209.755 67.1777 1 101

48 3 198.809 214.645 71.2292 1 47 84 3 198.315 215.074 71.4178 1 83 84 3 197.116 217.64 72.8645 1 83 88 3 202.193 215.175 72.2344 1 87 91 3 195.166 214.713 71.2611 1 90 91 3 197.288 215.964 73.5567 1 90 93 3 199.311 214.587 73.7479 1 92 103 3 200.808 213.972 74.0878 1 102 103 3 192.814 207.533 73.5725 1 102 103 3 191.022 208.138 72.737 1 102

49 3 198.55 214.45 76.2186 1 48 85 3 197.392 213.394 76.16 1 84 85 3 197.106 216.97 77.1598 1 84 89 3 200.694 214.973 77.8621 1 88 92 3 195.959 212.811 76.851 1 91 92 3 196.75 215.663 78.5873 1 91 94 3 199.233 213.787 79.3825 1 93 104 3 200.73 213.172 79.7224 1 103 104 3 189.567 207.391 78.3957 1 103 104 3 189.567 207.391 78.3957 1 103

50 3 198.291 214.255 81.2081 1 49 86 3 197.133 213.199 81.1495 1 85 86 3 196.847 216.775 82.1493 1 85 90 3 199.53 215.209 82.9992 1 89 93 3 195.226 211.919 84.3726 1 92 93 3 194.944 215.378 85.0573 1 92 95 3 197.178 212.274 85.4218 1 94 105 3 199.352 211.299 86.2951 1 104 105 3 186.637 206.308 83.567 1 104 105 3 186.637 206.308 83.567 1 104

51 3 198.094 214.093 86.2016 1 50 87 3 196.936 213.037 86.143 1 86 87 3 195.538 216.914 86.8792 1 86 91 3 198.003 213.48 87.5703 1 90 94 3 194.336 212.825 88.5425 1 93 94 3 194.04 215.793 89.9142 1 93 96 3 195.916 213.195 90.8469 1 95 106 3 199.424 210.375 92.0819 1 105 106 3 184.098 206.155 87.8253 1 105 106 3 185.42 204.778 88.4927 1 105

52 3 197.898 213.931 91.1951 1 51 88 3 196.047 213.898 92.3383 1 87 88 3 194.653 215.366 92.5034 1 87 92 3 196.61 213.093 93.1764 1 91 95 3 194.655 212.609 93.0996 1 94 95 3 193.094 216.801 93.4526 1 94 97 3 194.97 214.203 94.3853 1 96 107 3 198.817 211.204 95.887 1 106 107 3 183.669 205.516 93.0655 1 106 107 3 184.423 204.863 93.2308 1 106

53 3 197.918 213.448 97.4862 1 52 89 3 195.018 215.842 97.9806 1 88 89 3 193.152 215.099 97.8084 1 88 93 3 197.263 213.293 99.0467 1 92 96 3 193.224 212.551 98.4139 1 95 96 3 191.935 217.602 98.0799 1 95 98 3 193.196 215.083 98.7827 1 97 108 3 196.475 212.487 101.635 1 107 108 3 182.244 204.996 97.7041 1 107 108 3 182.998 204.343 97.8694 1 107

54 3 195.282 215.362 101.952 1 53 90 3 194.686 216.038 101.803 1 89 90 3 193.141 214.644 101.992 1 89 94 3 194.327 215.367 103.159 1 93 97 3 191.187 211.818 102.573 1 96 97 3 190.06 217.45 102.383 1 96 99 3 190.754 216.235 102.7 1 98 109 3 193.136 214.342 104.774 1 108 109 3 182.464 204.032 101.622 1 108 109 3 182.635 204.739 101.616 1 108

55 3 192.899 216.539 106.186 1 54 91 3 192.899 216.539 106.186 1 90 91 3 192.196 215.594 106.621 1 90 95 3 191.678 216.717 106.533 1 94 98 3 187.588 215.258 104.987 1 97 98 3 189 219.094 105.769 1 97 100 3 186.855 217.139 106.398 1 99 110 3 190.328 215.087 109.202 1 109 110 3 180.172 206.425 106.34 1 109 110 3 180.692 204.647 106.944 1 109

56 3 190.515 217.717 110.421 1 55 92 3 191.078 216.958 110.556 1 91 92 3 188.449 217.695 109.728 1 91 96 3 188.449 217.695 109.728 1 95 99 3 187.357 215.179 110.272 1 98 99 3 186.715 220.1 111.628 1 98 101 3 183.571 218.259 110.313 1 100 111 3 188.904 215.12 114.772 1 110 111 3 177.318 208.457 110.853 1 110 111 3 178.224 204.489 111.422 1 110

57 3 188.131 218.894 114.656 1 56 93 3 188.694 218.135 114.791 1 92 93 3 186.321 219.339 115.007 1 92 97 3 186.176 217.676 115.593 1 96 100 3 184.763 214.686 114.844 1 99 100 3 183.954 221.002

116.589 1 99 102 3 180.922 219.174 115.07 1 101 112 3 186.255 216.035 119.529 1 111 112 3 174.421 208.458 115.201 1 111 112 3 173.781 206.464 116.447 1 111

58 3 186.51 219.694 119.317 1 57 94 3 185.479 217.944 119.798 1 93 94 3 183.988 220.419 119.571 1 93 98 3 184.742 218.459 120.165 1 97 101 3 183.309 215.292 118.981 1 100 101 3 182.194 220.816 120.669 1 100 103 3 180.016 218.857 119.35 1 102 113 3 183.906 216.536 122.203 1 112 113 3 172.398 206.826 119.466 1 112 113 3 169.794 206.993 121.332 1 112

59 3 184.889 220.494 123.979 1 58 95 3 183.948 217.705 123.964 1 94 95 3 181.668 220.061 123.472 1 94 99 3 183.021 217.903 124.071 1 98 102 3 181.588 214.736 122.887 1 101 102 3 180.473 220.26 124.575 1 101 104 3 177.197 218.454 124.871 1 103 114 3 180.916 216.07 125.542 1 113 114 3 170.38 206.49 124.53 1 113 114 3 167.697 207.431 126.17 1 113

60 3 183.513 220.88 128.77 1 59 96 3 182.572 218.091 128.755 1 95 96 3 180.152 220.414 128.08 1 95 100 3 181.704 218.382 129.186 1 99 103 3 180.271 215.215 128.002 1 102 103 3 178.375 221.277 128.789 1 102 105 3 175.654 218.5 129.339 1 104 115 3 180.357 215.436 129.556 1 114 115 3 170.109 206.762 128.924 1 114 115 3 166.192 207.641 129.792 1 114

61 3 182.136 221.266 133.562 1 60 97 3 181.279 217.907 133.236 1 96 97 3 178.019 220.711 132.033 1 96 101 3 180.164 218.472 133.459 1 100 104 3 178.731 215.305 132.275 1 103 104 3 177.888 220.964 132.785 1 103 106 3 175.167 218.187 133.335 1 105 116 3 177.637 216.619 133.972 1 115 116 3 170.01 206.768 132.963 1 115 116 3 164.708 206.38 133.284 1 115

62 3 181.196 221.274 136.868 1 61 98 3 179.967 217.147 136.663 1 97 98 3 177.617 220.711 135.836 1 97 102 3 178.521 216.062 136.443 1 101 105 3 177.403 215.232 136.036 1 104 105 3 178.323 222.003 137.529 1 104 107 3 174.124 217.431 138.25 1 106 117 3 174.325 215.551 136.87 1 116 117 3 169.526 206.653 137.138 1 116 117 3 163.454 205.306 136.958 1 116

63 3 181.061 225.15 142.358 1 62 99 -4 181.061 225.15 142.358 1 98 99 0 X X X X X 103 0 X X X X X 106 0 X X X X X 106 0 X X X X X 108 0 X X X X X 118 0 X X X X X 118 0 X X X X X 118 0 X X X X X

64 3 180.509 227.811 146.555 1 63 100 -4 180.509 227.811 146.555 1 99 100 0 X X X X X 104 0 X X X X X 107 0 X X X X X 107 0 X X X X X 109 0 X X X X X 119 0 X X X X X 119 0 X X X X X 119 0 X X X X X

65 3 180.281 230.636 150.674 1 64 101 -4 180.281 230.636 150.674 1 100 101 0 X X X X X 105 0 X X X X X 108 0 X X X X X 108 0 X X X X X 110 0 X X X X X 120 0 X X X X X 120 0 X X X X X 120 0 X X X X X

66 3 180.053 233.462 154.792 1 65 102 -4 180.053 233.462 154.792 1 101 102 0 X X X X X 106 0 X X X X X 109 0 X X X X X 109 0 X X X X X 111 0 X X X X X 121 0 X X X X X 121 0 X X X X X 121 0 X X X X X

67 3 179.278 236.005 159.027 1 66 103 -4 179.278 236.005 159.027 1 102 103 0 X X X X X 107 0 X X X X X 110 0 X X X X X 110 0 X X X X X 112 0 X X X X X 122 0 X X X X X 122 0 X X X X X 122 0 X X X X X

68 3 178.487 238.208 163.445 1 67 104 -4 178.487 238.208 163.445 1 103 104 0 X X X X X 108 0 X X X X X 111 0 X X X X X 111 0 X X X X X 113 0 X X X X X 123 0 X X X X X 123 0 X X X X X 123 0 X X X X X

69 3 177.537 240.56 169.321 1 68 105 -4 177.537 240.56 169.321 1 104 105 0 X X X X X 109 0 X X X X X 112 0 X X X X X 112 0 X X X X X 114 0 X X X X X 124 0 X X X X X 124 0 X X X X X 124 0 X X X X X

70 3 179.098 218.954 141.627 1 62 106 3 177.88 216.017 141.864 1 98 106 3 175.749 220.776 140.431 1 98 110 3 175.765 215.817 139.435 1 102 113 3 175.269 214.728 139.217 1 105 113 3 177.441 222.232 142.51 1 105 115 3 172.359 214.875 142.131 1 107 125 3 170.992 213.512 140.864 1 117 125 3 168.158 203.057 142.068 1 117 125 3 162.998 203.423 140.773 1 117

71 3 177.452 217.208 146.014 1 70 107 3 176.771 214.738 147.596 1 106 107 3 174.64 219.497 146.163 1 106 111 3 173.447 212.769 142.544 1 110 114 3 173.447 212.769 142.544 1 113 114 3 176.465 220.843 146.701 1 113 116 3 169.949 212.146 145.131 1 115 126 3 168.177 211.185 144.58 1 125 126 3 165.436 200.228 145.432 1 125 126 3 161.251 201.993 145.265 1 125

72 3 175.003 215.448 151.744 1 71 108 3 176.237 214.313 151.489 1 107 108 3 174.106 219.072 150.056 1 107 112 3 170.453 210.777 146.214 1 111 115 3 170.453 210.777 146.214 1 114 115 3 173.471 218.851 150.371 1 114 117 3 166.955 210.154 148.801 1 116 127 3 164.435 208.132 147.351 1 126 127 3 161.694 197.175 148.203 1 126 127 3 160.128 200.661 149.081 1 126

73 3 173.143 213.015 156.271 1 72 109 3 174.955 211.995 156.345 1 108 109 3 171.523 216.698 153.946 1 108 113 3 167.287 209.123 150.637 1 112 116 3 166.498 207.927 149.283 1 115 116 3 170.143 216.003 155.238 1 115 118 3 164.725 207.996 154.505 1 117 128 3 161.411 203.774 151.732 1 127 128 3 158.28 191.76 151.429 1 127 128 3 156.723 197.902 152.204 1 127

74 3 172.811 210.836 158.629 1 73 110 -4 172.811 210.836 158.629 1 109 110 0 X X X X X 114 0 X X X X X 117 0 X X X X X 117 0 X X X X X 119 0 X X X X X 129 0 X X X X X 129 0 X X X X X 129 0 X X X X X

75 3 171.074 207.973 162.342 1 74 111 -4 171.074 207.973 162.342 1 110 111 0 X X X X X 115 0 X X X X X 118 0 X X X X X 118 0 X X X X X 120 0 X X X X X 130 0 X X X X X 130 0 X X X X X 130 0 X X X X X

76 3 169.338 205.11 166.055 1 75 112 -4 169.338 205.11 166.055 1 111 112 0 X X X X X 116 0 X X X X X 119 0 X X X X X 119 0 X X X X X 121 0 X X X X X 131 0 X X X X X 131 0 X X X X X 131 0 X X X X X

77 3 168.073 201.777 169.417 1 76 113 -4 168.073 201.777 169.417 1 112 113 0 X X X X X 117 0 X X X X X 120 0 X X X X X 120 0 X X X X X 122 0 X X X X X 132 0 X X X X X 132 0 X X X X X 132 0 X X X X X

78 3 173.068 212.914 160.971 1 73 114 3 173.097 211.204 161.251 1 109 114 3 168.647 215.377 156.608 1 109 118 3 166.081 207.488 154.412 1 113 121 3 164.523 204.674 153.566 1 116 121 3 167.446 213.602 159.17 1 116 123 3 162.874 207.076 158.851 1 118 133 3 159.164 202.187 155.504 1 128 133 3 156.033 190.173 155.201 1 128 133 3 153.404 192.931 155.721 1 128

79 3 172.39 212.817 165.924 1 78 115 3 171.366 210.565 165.401 1 114 115 3 164.773 214.98 159.417 1 114 119 3 164.481 207.693 158.54 1 118 122 3 162.368 203.791 157.742 1 121 122 3 165.753 212.358 164.099 1 121 124 3 161.181 205.832 163.78 1 123 134 3 156.978 200.71 160.424 1 133 134 3 153.847 188.696 160.121 1 133 134 3 151.121 190.387 158.179 1 133

80 3 171.596 212.953 170.859 1 79 116 3 169.862 208.976 169.674 1 115 116 3 161.588 213.727 163.444 1 115 120 3 164.226 207.99 163.616 1 119 123 3 161.344 202.983 161.798 1 122 123 3 165.382 211.624 168.754 1 122 125 3 159.824 204.914 168.499 1 124 135 3 156.6 200.608 165.204 1 134 135 3 152.995 187.487 163.632 1 134 135 3 148.72 187.193 161.984 1 134

81 3 170.747 211.768 175.823 1 80 117 3 169.013 207.791 174.638 1 116 117 3 160.221 211.736 166.426 1 116 121 3 163.201 205.791 168.153 1 120 124 3 160.783 201.605 166.106 1 123 124 3

164.594 211.142 172.989 1 123 126 3 159.036 204.432 172.734 1 125 136 3 153.86 199.81 169.258 1 135 136 3 150.255 186.689 167.686 1 135 136 3 146.565 184.89 165.801 1 135

82 3 169.891 213.063 178.955 1 81 118 3 168.128 204.423 179.156 1 117 118 3 160.175 211.841 172.089 1 117 122 3 160.707 203.84 171.022 1 121 125 3 159.113 201.125 169.493 1 124 125 3 163.471 209.41 176.186 1 124 127 3 158.14 201.319 177.226 1 126 137 3 151.386 196.741 173.448 1 136 137 3 147.896 182.787 171.167 1 136 137 3 143.959 182.442 169.502 1 136

0 0 X X X X X 119 -1 167.208 202.357 182.143 1.05264 118 119 3 159.493 211.349 176.055 1.05264 118 123 3 158.877 200.184 174.012 1.05264 122 126 3 158.13 199.123 173.348 1.05264 125 126 3 162.488 207.408 180.041 1.05264 125 128 3 156.531 199.711 181.7 1.05264 127 138 3 148.826 192.998 177.063 1.05264 137 138 3 144.886 179.75 173.141 1.05264 137 138 3 141.967 179.068 174.319 1.05264 137

0 0 X X X X X 120 -1 166.261 199.648 186.238 1.05264 119 120 3 158.65 209.574 180.27 1.05264 119 124 3 156.562 197.413 178.453 1.05264 123 127 3 155.815 196.352 177.789 1.05264 126 127 3 160.895 204.06 184.525 1.05264 126 129 3 153.742 197.342 184.041 1.05264 128 139 3 145.856 190.878 178.664 1.05264 138 139 3 141.916 177.63 174.742 1.05264 138 139 3 138.289 174.881 178.911 1.05264 138

0 0 X X X X X 121 -1 164.86 197.269 190.406 1.00651 120 121 3 158.409 208.065 186.15 1.00651 120 125 3 154.351 195.64 181.85 1.00651 124 128 3 153.604 194.579 181.186 1.00651 127 128 3 158.684 202.287 187.922 1.00651 127 130 3 151.531 195.569 187.438 1.00651 129 140 3 142.695 188.452 180.053 1.00651 139 140 3 138.755 175.204 176.131 1.00651 139 140 3 133.185 170.53 182.909 1.00651 139

0 0 X X X X X 122 -1 164.169 195.017 194.817 1.15981 121 122 3 156.791 205.967 189.287 1.15981 121 126 3 152.035 194.185 185.257 1.15981 125 129 3 150.361 189.569 184.505 1.15981 128 129 3 155.716 200.906 193.365 1.15981 128 131 3 147.438 193.255 192.032 1.15981 130 141 3 138.628 186.113 181.395 1.15981 140 141 3 135.735 171.971 180.115 1.15981 140 141 3 128.717 166.967 187.037 1.15981 140

0 0 X X X X X 123 -1 162.975 193.633 199.47 1.15981 122 123 3 154.331 202.619 191.789 1.15981 122 127 3 149.783 192.672 191.272 1.15981 126 130 3 147.643 186.859 190.158 1.15981 129 130 3 153.588 198.525 199.262 1.15981 129 132 3 144.939 190.898 195.199 1.15981 131 142 3 136.129 183.756 184.562 1.15981 141 142 3 129.612 169.811 182.63 1.15981 141 142 3 124.803 165.343 189.427 1.15981 141

0 0 X X X X X 124 -1 161.302 191.694 205.985 1.15981 123 124 3 152.469 201.538 193.681 1.15981 123 128 3 148.952 193.039 196.831 1.15981 127 131 3 146.263 185.179 196.382 1.15981 130 131 3 151.518 197.069 205.097 1.15981 130 133 3 142.321 190.026 197.923 1.15981 132 143 3 135.348 184.545 189.501 1.15981 142 143 3 126.999 168.305 186.303 1.15981 142 143 3 122.09 164.433 191.864 1.15981 142

0 0 X X X X X 0 0 X X X X X 0 0 X X X X X 0 0 X X X X X 132 -1 147.109 183.68 199.572 1 131 132 3 149.575 196.168 209.106 1 131 134 3 142.09 190.049 201.134 1 133 144 3 134.604 186.224 194.125 1 143 144 3 123.687 168.001 190.705 1 143 144 3 119.475 165.076 196.424 1 143

0 0 X X X X X 0 0 X X X X X X 0 0 X X X X X X 0 0 X X X X X X 133 -1 147.157 183.967 202.97 1 132 133 3 147.532 195.387 214.221 1 132 135 3 142.204 190.148 205.058 1 134 145 3 133.687 187.175 198.392 1 144 145 3 122.208 172.875 195.498 1 144 145 3 117.299 169.003 201.059 1 144

0 0 X X X X X 0 0 X X X X X X 0 0 X X X X X X 0 0 X X X X X X 134 -1 147.252 185.54 206.766 1 133 134 3 147.714 194.59 219.767 1 133 136 3 141.098 190.341 212.148 1 135 146 3 131.978 188.033 203.229 1 145 146 3 121.102 177.158 197.618 1 145 146 3 116.193 173.286 203.179 1 145

0 0 X X X X X 0 0 X X X X X X 0 0 X X X X X X 0 0 X X X X X X 0 0 X X X X X X 135 -1 148.008 194.642 224.012 1 134 137 3 140.211 190.669 217.192 1 136 147 3 131.783 189.242 207.531 1 146 147 3 119.706 179.822 201.566 1 146 147 3 114.797 175.95 207.127 1 146

0 0 X X X X X 0 0 X X X X X X 0 0 X X X X X X 0 0 X X X X X X 0 0 X X X X X X 136 -1 148.478 194.19 228.011 1 135 138 3 140.147 192.407 222.203 1 137 148 3 131.572 190.314 212.041 1 147 148 3 119.397 183.084 204.848 1 147 148 3 114.488 179.212 210.409 1 147

0 0 X X X X X 0 0 X X X X X X 0 0 X X X X X X 0 0 X X X X X X 0 0 X X X X X X 137 -1 148.448 194.315 232.605 1.5 136 139 3 141.91 194.234 225.669 1 138 149 3 132.039 188.935 219.195 1 148 149 3 118.471 183.131 210.868 1 148 149 3 111.994 184.764 215.36 1 148

83 3 204.326 209.122 30.5893 1 4 125 3 204.766 208.539 28.9869 1 4 125 3 204.569 209.467 30.1553 1 4 129 3 204.592 208.897 29.3204 1 4 135 3 204.592 208.897 29.3204 1 4 138 3 203.804 209.397 30.7089 1 4 140 3 202.576 209.247 29.5857 1 4 150 3 204.96 207.81 28.7694 1 4 150 3 203.218 208.871 28.7998 1 4 150 3 202.22 208.082 28.5893 1 4

84 3 201.189 207.941 34.3 1 83 126 3 201.771 206.586 31.211 1 125 126 3 200.843 209.356 31.3605 1 125 130 3 200.734 209.03 32.0946 1 129 136 3 201.174 208.013 31.1576 1 135 139 3 200.318 208.221 32.4226 1 138 141 3 198.899 207.975 30.81 1 140 151 3 201.882 206.708 30.4241 1 150 151 3 200.092 207.213 29.6904 1 150 151 3 199.364 207.137 30.5725 1 150

85 3 198.167 207.164 38.2066 1 84 127 3 199.19 205.226 33.5151 1 126 127 3 197.831 209.411 33.8578 1 126 131 3 198.138 208.573 35.3064 1 130 137 3 197.734 207.213 32.7043 1 136 140 3 197.155 208.178 34.1579 1 139 142 3 195.736 207.932 32.5453 1 141 152 3 199.465 205.435 31.8845 1 151 152 3 197.675 205.94 31.1508 1 151 152 3 195.602 205.194 33.0258 1 151

86 3 194.783 205.861 41.6495 1 85 128 3 197.296 204.866 36.3789 1 127 128 3 196.277 208.44 39.2137 1 127 132 3 195.713 206.934 39.0685 1 131 138 3 196.205 206.547 35.3209 1 137 141 3 194.722 206.818 37.7143 1 140 143 3 194.722 206.818 37.7143 1 142 153 3 197.758 205.346 33.1122 1 152 153 3 195.244 204.066 32.5783 1 152 153 3 192.409 203.56 36.5753 1 152

87 3 193.828 205.728 44.0134 1 86 129 3 196.048 204.253 39.9991 1 128 129 3 194.761 207.498 44.6478 1 128 133 3 193.484 206.171 42.278 1 132 139 3 194.3 206.486 38.3244 1 138 142 3 191.673 206.971 41.2791 1 141 144 3 191.345 206.108 42.2348 1 143 154 3 196.223 205.259 34.9927 1 153 154 3 190.964 202.41 35.3674 1 153 154 3 188.168 202.387 40.5983 1 153

88 3 192.386 204.302 45.7509 1 87 130 3 193.258 201.9 45.517 1 129 130 3 190.292 209.777 46.6025 1 129 134 -2 190.605 207.53 43.3955 1 133 140 3 191.125 207.027 41.967 1 139 143 3 189.561 208.833 41.8659 1 142 145 3 190.174 207.408 42.0121 1 144 155 3 193.67 206.797 38.3178 1 154 155 3 187.889 203.612 37.4515 1 154 155 3 185.587 203.488 41.8962 1 154

89 3 189.988 202.743 49.8522 1 88 131 3 190.611 199.67 49.3106 1 130 131 3 184.995 211.439 49.177 1 130 135 -2 188.172 208.977 45.5586 1 134 141 3 186.038 207.31 44.7472 1 140 144 3 185.893 210.161 44.1836 1 143 146 3 187.486 209.762 43.6782 1 145 156 3 190.079 207.447 40.4021 1 155 156 3 184.66 204.338 39.8247 1 155 156 3 183.393 204.897 42.9289 1 155

90 3 187.59 201.184 53.9535 1 89 132 3 188.213 198.111 53.4119 1 131 132 3 180.423 211.821 51.8561 1 131 136 -2 186.311 209.974 47.18 1 135 142 3 183.315 207.815 46.3657 1 141 145 3 182.018 211.044 45.3649 1 144 147 3 184.229 210.419 44.2677 1 146 157 3 186.484 208.445 43.3003 1 156 157 3 180.504 205.261 42.2878 1 156 157 3 180.42 204.881 44.1061 1 156

91 3 185.004 199.2 57.7452 1 90 133 3 185.876 196.798 57.5113 1 132 133 3 176.135 210.432 54.8438 1 132 137 -2 184.418 210.826 48.6731 1 136 143 3 179.887 208.742 49.5522 1 142 146 3 178.602 212.183 48.3729 1 145 148 3 182.928 211.589 44.931 1 147 158 3 179.686 208.593 46.2456 1 157 158 3 178.053 206.454 43.5608 1 157 158 3 177.516 204.939 44.9641 1 157

92 3 183.054 197.704 62.0993 1 91 134 3 183.824 193.57 61.8395 1 133 134 3 173.272 209.232 54.478 1 133 138 3 180.478 209.416 48.7556 1 137 144 3 176.896 208.073 49.9954 1 143 147 3 176.063 211.412 48.9777 1 146 149 3 175.909 209.59 46.517 1 148 159 3 176.758 208.595 46.6876 1 158 159 3 174.386 204.997 43.0121 1 158 159 3 171.609 202.796 45.4513 1 158

93 3 181.598 195.973 66.5582 1 92 135 3 182.368 191.839 66.2984 1 134 135 3 168.652 209.296 54.3915 1 134 139 3 175.451 209.211 49.3907 1 138 145 3 171.96 206.999 50.4727 1 144 148 3 173.273 210.025 49.3336 1 147 150 3 172.658 208.217 46.9672 1 149 160 3 173.519 207.641 46.289 1 159 160 3 170.236 203.762 44.0211 1 159 160 3 167.266 201.437 45.4088 1 159

94 3 180.347 194.087 71.017 1 93 136 3 181.117 189.953 70.7572 1 135 136 3 163.986 209.095 55.1219 1 135 140 3 169.508 207.292 51.122 1 139 146 3 168.764 206.014 51.0691 1 145 149 3 170.659 209.224 49.8082 1 148 151 3 167.992 207.932 47.0403 1 150 161 3 169.674 206.65 45.4285 1 160 161 3 166.865 202.773 44.6418 1 160 161 3 162.104 200.16 46.2497 1 160

95 3 179.097 192.201 75.4759 1 94 137 3 179.867 188.067 75.2161 1 136 137 3 160.817 208.092 56.742 1 136 141 3 164.343 205.253 53.6804 1 140 147 3 163.649 204.278 52.7159 1 146 150 3 167.786 208.584 50.8576 1 149 152 3 161.447 207.095 48.0232 1 151 162 3 166.486 205.432 46.5319 1 161 162 3 161.805 200.515 46.1785 1 161 162 3 157.619 198.653 47.0481 1 161

96 3 178.208 190.757 80.1795 1 95 138 3 179.17 185.59 79.8548 1 137 138 3 157.127 208.075 56.24 1 137 142 3 160.382 206.152 54.6072 1 141 148 3 158.217 204.616 53.6256 1 147 151 3 163.471 209.044 51.2928 1 150 153 3 158.443 208.44 49.7392 1 152 163 3 161.437 205.747 48.4257 1 162 163 3 155.163 202.181 46.5207 1 162 163 3 151.951 201.616 48.9578 1 162

97 3 177.463 188.878 84.7524 1 96 139 3 178.425 183.711 84.4277 1 138 139 3 153.213 208.966 57.3766 1 138 143 3 156.468 207.043 55.7438 1 142 149 3 154.09 205.015 53.424 1 148 152 3 159.344 209.443 51.0912 1 151 154 3 156.243 209.454 51.8375 1 153 164 3 157.837 206.32 49.3337 1 163 164 3 151.563 202.754 47.4287 1 163 164 3 148.118 204.298 50.1066 1 163

98 3 176.558 187.07 89.3253 1 97 140 3 177.328 182.936 89.0655 1 139 140 3 149.259 209.855 57.9253 1 139 144 3 151.893 206.656 55.9943 1 143 150 3 148.987 205.537 53.6936 1 149 153 3 154.241

209.965 51.3608 1 152 155 3 152.215 211.835 53.0605 1 154 165 3 150.196 209.937 51.9325 1 164 165 3 145.567 206.916 49.9425 1 164 165 3 142.425 206.807 52.4246 1 164

99 3 175.653 185.262 93.8982 1 98 141 3 176.688 181.739 93.7015 1 140 141 3 144.93 211.295 58.8804 1 140 145 3 146.252 212.986 57.4163 1 144 151 3 144.007 209.635 57.8112 1 150 154 3 148.522 212.447 54.1128 1 153 156 3 148.133 214.358 55.1775 1 155 166 3 146.478 212.516 53.4811 1 165 166 3 140.539 210.346 52.0617 1 165 166 3 137.327 209.781 54.4988 1 165

100 3 174.748 183.454 98.4711 1 99 142 3 175.637 180.734 98.1311 1 141 142 3 139.814 215.927 60.3451 1 141 146 3 141.136 217.618 58.881 1 145 152 3 140.08 213.114 60.4895 1 151 155 3 143.652 217.428 55.0769 1 154 157 3 143.635 216.617 56.6057 1 156 167 3 142.261 215.575 54.8566 1 166 167 3 136.322 213.405 53.4372 1 166 167 3 132.637 212.303 55.0985 1 166

101 3 173.952 181.811 103.126 1 100 143 3 174.727 180.099 103.006 1 142 143 3 133.034 219.978 61.5938 1 142 147 3 134.94 221.82 60.0891 1 146 153 3 133.729 217.208 61.8349 1 152 156 3 138.079 221.75 56.6983 1 155 158 3 137.532 219.978 60.258 1 157 168 3 135.91 219.2 56.8931 1 167 168 3 130.651 216.351 57.2678 1 167 168 3 125.823 214.204 56.817 1 167

102 3 173.157 180.169 107.781 1 101 144 3 173.932 178.457 107.661 1 143 144 3 129.747 223.832 63.9633 1 143 148 3 129.057 223.507 61.119 1 147 154 3 127.699 219.653 63.2307 1 153 157 3 132.049 224.195 58.0941 1 156 159 3 131.903 223.332 62.8152 1 158 169 3 129.954 222.088 59.4005 1 168 169 3 124.701 219.337 58.7011 1 168 169 3 120.256 216.712 59.3002 1 168

103 3 172.362 178.526 112.436 1 102 145 3 173.137 176.814 112.316 1 144 145 3 125.105 225.035 64.8695 1 144 149 3 124.415 224.71 62.0252 1 148 155 3 123.668 221.492 64.5236 1 154 158 3 127.315 224.432 58.941 1 157 160 3 128.276 225.281 64.1294 1 159 170 3 126.327 224.037 60.7147 1 169 170 3 119.322 220.07 59.5489 1 169 170 3 115.091 218.827 60.5175 1 169

104 3 171.567 176.884 117.092 1 103 146 3 172.342 175.172 116.972 1 145 146 3 120.535 226.348 66.6906 1 145 150 3 119.519 225.677 63.8263 1 149 156 3 118.772 222.459 66.3247 1 155 159 3 122.419 225.399 60.7421 1 158 161 3 123.443 226.973 64.4969 1 160 171 3 122.54 226.03 62.4247 1 170 171 3 114.142 220.909 59.1961 1 170 171 3 111.235 220.029 62.3163 1 170

105 3 171.18 175.233 121.795 1 104 147 3 171.955 173.521 121.675 1 146 147 3 116.216 226.516 67.7044 1 146 151 3 114.835 225.881 64.0385 1 150 157 3 114.878 223.842 67.2401 1 156 160 3 119.061 226.545 59.6672 1 159 162 3 119.288 229.081 64.8791 1 161 172 3 118.888 227.91 62.9757 1 171 172 3 109.171 223.491 60.4492 1 171 172 3 106.264 222.611 63.5694 1 171

106 3 170.984 173.504 126.483 1 105 148 3 171.759 171.792 126.363 1 147 148 3 112.316 228.178 68.8157 1 147 152 3 109.861 227.413 64.4168 1 151 158 3 109.769 225.556 68.8487 1 157 161 3 113.952 228.259 61.2758 1 160 163 3 114.413 231.605 66.0734 1 162 173 3 114.528 228.59 64.4315 1 172 173 3 103.351 225.014 60.5744 1 172 173 3 99.4641 223.991 64.5543 1 172

107 3 171.059 171.926 131.227 1 106 149 3 171.834 170.214 131.107 1 148 149 3 108.039 229.942 69.7182 1 148 153 3 106.26 229.098 65.2428 1 152 159 3 106.055 226.164 69.9099 1 158 162 3 110.485 229.319 62.4576 1 161 164 3 111.025 232.781 67.066 1 163 174 3 111.14 229.766 65.4241 1 173 174 3 97.2002 229.08 63.6656 1 173 174 3 92.5182 225.149 65.1179 1 173

108 3 171.133 170.347 135.97 1 107 150 3 171.908 168.635 135.85 1 149 150 3 104.243 229.996 70.0926 1 149 154 3 103.273 229.105 66.5302 1 153 160 3 102.867 226.172 71.3162 1 159 163 3 106.34 229.939 62.5074 1 162 165 3 108.103 234.762 67.396 1 164 175 3 107.802 231.505 65.8244 1 174 175 3 93.2949 231.688 65.2374 1 174 175 3 88.6129 227.757 66.6897 1 174

109 3 171.068 168.742 140.705 1 108 151 3 171.132 166.425 140.154 1 150 151 3 100.104 231.178 71.4697 1 150 155 3 99.9433 229.477 66.955 1 154 161 3 99.4575 226.513 72.534 1 160 164 3 103.376 231.791 64.1324 1 163 166 3 104.548 235.103 68.5802 1 165 176 3 102.906 232.822 67.6596 1 175 176 3 90.8732 234.296 67.4007 1 175 176 3 86.4072 231.808 68.7548 1 175

110 3 171.011 166.974 145.382 1 109 152 3 171.075 164.657 144.831 1 151 152 3 96.3347 231.942 74.0609 1 151 156 3 97.9065 230.67 67.8001 1 155 162 3 95.3304 227.141 74.5181 1 161 165 3 99.9053 235.233 66.6217 1 164 167 3 99.8417 238.285 69.4817 1 166 177 3 99.3391 236.784 67.8153 1 176 177 3 87.7664 238.008 68.4856 1 176 177 3 83.7251 235.698 70.8598 1 176

111 3 171.006 164.937 150.598 1 110 153 3 171.07 162.62 150.047 1 152 153 3 93.7881 230.887 75.6814 1 152 157 3 94.7533 226.994 69.707 1 156 163 3 92.7595 225.037 75.5078 1 162 166 -4 96.778 232.274 65.2502 1 165 168 0 X X X X X 178 0 X X X X X 178 0 X X X X X 178 0 X X X X X

0 0 X X X X X 154 -1 171 161 153 1 153 154 3 90.6342 229.889 77.6896 1 153 158 3 90.3372 224.129 71.2989 1 157 164 3 89.0403 222.274 76.444 1 163 167 -4 93.0588 229.511 66.1864 1 166 169 0 X X X X X 179 0 X X X X X 179 0 X X X X X 179 0 X X X X X

0 0 X X X X X 155 -1 171.298 159.842 157.648 1 154 155 3 86.9156 228.682 79.6329 1 154 159 3 87.4799 224.095 74.1356 1 158 165 -4 85.932 221.382 77.3296 1 164 168 0 X X X X X 170 0 X X X X X 180 0 X X X X X 180 0 X X X X X 180 0 X X X X X

0 0 X X X X X 156 -1 171.98 158.547 161.462 1.1 155 156 3 81.9338 227.407 80.9978 1 155 160 3 83.291 222.919 77.7327 1 159 166 -4 81.7431 220.206 80.9267 1 165 169 0 X X X X X 171 0 X X X X X 181 0 X X X X X 181 0 X X X X X 181 0 X X X X X

0 0 X X X X X 157 -1 172.001 156.708 165.57 1 156 157 3 76.2363 226.428 81.6225 1 156 161 3 78.2938 221.604 80.3649 1 160 167 -4 76.7459 218.891 83.5589 1 166 170 0 X X X X X 172 0 X X X X X 182 0 X X X X X 182 0 X X X X X 182 0 X X X X X

112 3 191 207 46.5 1 87 158 3 191.396 205.692 45.9519 1 129 158 3 71.3938 225.44 83.8848 1 157 162 3 75.0602 220.824 80.8447 1 161 168 -4 73.5123 218.111 84.0387 1 167 171 0 X X X X X 173 0 X X X X X 183 0 X X X X X 183 0 X X X X X 183 0 X X X X X

113 3 187 207 49 1 112 159 3 187.378 206.375 48.5431 1 158 159 -4 67.6118 224.83 85.6225 1 158 163 0 X X X X X 169 0 X X X X X 172 0 X X X X X 174 0 X X X X X 184 0 X X X X X 184 0 X X X X X 184 0 X X X X X

114 3 185 208 49.6667 1 113 160 3 184.176 207.182 50.4289 1 159 160 -4 62.6118 223.83 86.7059 0.6 159 164 0 X X X X X 170 0 X X X X X 173 0 X X X X X 175 0 X X X X X 185 0 X X X X X 185 0 X X X X X 185 0 X X X X X

115 3 182.357 207.637 52.4937 1 114 161 3 182.357 207.637 52.4937 1 160 161 3 91.6631 235.049 75.0311 1 152 165 3 95.9419 234.849 68.0351 1 156 171 3 91.8793 231.095 73.7783 1 162 174 3 97.555

236.949 67.5579 1 165 176 3 96.781 239.903 70.6497 1 167 186 3 96.363 240.101 69.8548 1 177 186 3 83.567 241.151 72.3465 1 177 186 3 79.2006 238.881 73.7127 1 177

116 3 178.654 206.731 52.1844 1 115 162 3 178.749 205.853 52.9598 1 161 162 3 88.7389 238.128 75.0062 1 161 166 3 93.0177 237.928 68.0102 1 165 172 3 89.2675 234.214 74.7333 1 171 175 3 93.4855 238.87 68.88 1 174 177 3 94.114 243.073 72.0682 1 176 187 3 93.9261 243.605 71.0752 1 186 187 3 78.0445 244.498 74.8879 1 186 187 3 74.4611 243.376 75.7665 1 186

117 3 173.865 205.352 51.7725 1 116 163 3 174.941 203.897 52.8437 1 162 163 3 84.5367 241.692 76.5645 1 162 167 3 89.0811 241.931 70.1401 1 166 173 3 84.5526 237.672 75.3108 1 172 176 3 88.7706 242.328 69.4575 1 175 178 3 90.1308 246.769 73.3233 1 177 188 3 90.3048 247.278 72.8233 1 187 188 3 75.6984 247.549 77.31 1 187 188 3 71.0689 245.956 78.2987 1 187

118 3 169.168 203.84 50.967 1 117 164 3 169.363 202.565 54.1914 1 163 164 3 80.6291 245.283 77.5637 1 163 168 3 85.5062 244.448 74.511 1 167 174 3 81.6335 241.043 77.0483 1 173 177 3 85.8515 245.699 71.195 1 176 179 3 87.2117 250.14 75.0608 1 178 189 3 87.0348 250.498 73.6716 1 188 189 3 72.9558 250.243 78.9144 1 188 189 3 68.3263 248.65 79.9031 1 188

119 3 163.276 202.975 51.2347 1 118 165 3 163.288 201.267 52.7795 1 164 165 3 75.8291 248.317 78.8732 1 164 169 3 81.4105 248.065 76.4422 1 168 175 3 77.5378 244.66 78.9795 1 174 178 3 82.7042 248.781 72.8026 1 177 180 3 84.0644 253.222 76.6684 1 179 190 3 84.0462 253.631 74.557 1 189 190 3 69.5947 252.372 80.1651 1 189 190 3 64.9652 250.779 81.1538 1 189

120 3 160.405 204.258 52.0962 1 119 166 3 156.895 202.399 52.5932 1 165 166 3 68.8557 252.489 80.5226 1 165 170 3 74.4371 252.237 78.0916 1 169 176 3 72.3464 248.367 81.4721 1 175 179 3 77.5128 252.488 75.2952 1 178 181 3 78.3551 253.773 78.7694 1 180 191 3 77.3811 253.766 76.9518 1 190 191 3 65.1926 253.481 82.1157 1 190 191 3 61.8866 252.556 84.5557 1 190

0 0 X X X X X 0 0 X X X X X 0 0 X X X X X 171 -1 72.7105 253.892 79.4094 0.628019 170 177 3 70.0771 250.661 83.5556 0.628019 176 180 3 72.2554 253.411 78.6109 0.628019 179 182 3 72.2377 253.659 79.8584 1.12802 181 192 3 72.1099 253.604 79.5777 1.12802 191 192 3 59.9214 253.319 84.7416 1.12802 191 192 3 57.7443 254.231 87.6575 0.62802 191

0 0 X X X X X 0 0 X X X X X 0 0 X X X X X 172 -1 64.7291 254.02 83.5665 0.628019 171 178 3 65.4051 253.544 85.4874 0.628019 177 181 3 67.1155 253.784 81.556 0.628019 180 183 3 67.1155 253.784 81.556 1.12802 182 193 3 66.9877 253.729 81.2753 1.12802 192 193 3 54.7992 253.444 86.4392 1.12802 192 193 3 53.3659 253.954 91.7925 0.62802 192

0 0 X X X X X 0 0 X X X X X 0 0 X X X X X 173 -1 60.6143 254.004 86.4069 0.605948 172 179 3 61.6635 253.64 88.5627 0.605948 178 182 3 61.9439 253.448 84.9556 0.605948 181 184 3 60.3026 253.625 83.1133 1.10595 183 194 3 60.9527 253.33 84.6209 1.10595 193 194 3 50.1623 253.281 90.5561 1.10595 193 194 3 48.729 253.791 95.9094 0.60595 193

0 0 X X X X X 0 0 X X X X X 0 0 X X X X X 174 -1 57.2763 254.019 90.1294 0.802458 173 180 3 55.6767 254.072 91.3342 0.802458 179 183 3 56.3435 253.431 87.8118 0.802458 182 185 3 55.7467 253.496 87.1419 0.802458 184 195 3 59.0201 254.021 90.4586 0.802458 194 195 3 47.1584 254.801 95.487 0.802458 194 195 3 44.6039 253.915 100.63 0.802458 194

0 0 X X X X X 0 0 X X X X X X 0 0 X X X X X 175 -1 53.0704 254.038 94.8198 0.802458 174 181 3 49.4045 254.547 94.1224 0.802458 180 184 3 52.3551 254.183 92.6728 0.802458 183 186 -4 52.2293 253.817 90.6282 0.802458 185 196 -5 56.3261 253.482 96.8233 0.802458 195 196 3 43.8901 254.315 99.9511 0.802458 195 196 3 42.1849 254.379 105.338 0.802458 195

0 0 X X X X X 0 0 X X X X X X 0 0 X X X X X X 0 0 X X X X X X 0 0 X X X X X 185 -1 51.1186 253.972 95.8447 0.6 184 187 -4 50.9928 253.606 93.8001 0.6 186 197 -5 55.625 253.155 101.074 1.1 196 197 3 41.9903 253.781 105.024 1.1 196 197 3 42.243 254.001 111.423 0.6 196

0 0 X X X X X 0 0 X X X X X X 0 0 X X X X X X 0 0 X X X X X X 0 0 X X X X X 186 -1 51.3558 253.915 100.534 1 185 188 -4 51.23 253.549 98.4894 1 187 198 -5 55.8335 253.112 110.344 1 197 198 3 39.8555 253.574 111.097 1 197 198 3 38.3794 253.642 115.779 1 197

0 0 X X X X X 0 0 X X X X X X 0 0 X X X X X X 0 0 X X X X X X 0 0 X X X X X X 0 0 X X X X X X 0 0 X X X X X X 0 0 X X X X X 199 -1 37.6785 253.969 117.557 1 198 199 3 33.9512 253.438 117.874 1 198

121 3 156.413 206.3 54.3086 1 120 167 -4 160.765 200.305 53.17 1 137 167 -5 158.105 204.749 57.6392 1 137 176 3 159.287 201.883 54.5488 1 141 182 3 159.287 201.883 54.5488 1 147 187 3 161.785 205.247 52.497 1 150 189 3 155.916 203.92 50.7519 1 152 199 3 163.705 202.019 46.3354 1 162 200 3 155.014 195.133 45.914 1 162 200 3 152.529 193.639 49.1455 1 162

122 3 152.422 208.343 56.5209 1 121 168 -4 158.581 198.976 54.878 1 167 168 -5 155.921 203.42 59.3472 1 167 177 3 155.381 199.549 55.8095 1 176 183 3 155.795 198.751 56.2251 1 182 188 3 155.177 201.18 54.5968 1 187 190 3 153.295 201.538 52.3587 1 189 200 3 161.084 199.637 47.9422 1 199 201 3 149.556 189.246 47.0068 1 200 201 3 146.248 189.079 50.782 1 200

123 3 148.587 210.505 58.892 1 122 169 -4 155.257 196.18 57.3537 1 168 169 -5 152.597 200.624 61.8229 1 168 178 3 150.534 196.888 57.9629 1 177 184 3 150.948 196.09 58.3785 1 183 189 3 150.33 198.519 56.7502 1 188 191 3 148.448 198.877 54.5121 1 190 201 3 156.237 196.976 50.0956 1 200 202 3 144.019 186 48.4976 1 201 202 3 141.576 184.734 52.8124 1 201

124 3 144.527 212.668 60.8504 1 123 170 -4 151.914 194.042 60.396 1 169 170 -5 149.254 198.486 64.8652 1 169 179 3 147.052 193.745 60.2511 1 178 185 3 147.052 193.745 60.2511 1 184 190 3 146.434 196.174 58.6228 1 189 192 3 144.552 196.532 56.3847 1 191 202 3 150.262 194.517 53.0484 1 201 203 3 138.252 183.544 50.0955 1 202 203 3 136.363 181.1 55.3963 1 202

125 3 140.719 214.697 63.3771 1 124 171 -4 148.613 191.508 63.1682 1 170 171 -5 145.55 196.272 67.4519 1 170 180 3 143.348 191.531 62.8378 1 179 186 3 143.348 191.531 62.8378 1 185 191 3 142.33 194.575 60.8196 1 190 193 3 140.448 194.933 58.5815 1 192 203 3 143.077 191.901 56.1235 1 202 204 3 130.626 181.938 51.4191 1 203 204 3 131.641 178.814 56.6265 1 203

126 3 136.285 216.35 64.9906 1 125 172 -4 144.805 189.313 65.5521 1 171 172 -5 142.104 193.816 69.1251 1 171 181 3 140.475 190.272 64.0532 1 180 187 3 140.475 190.272 64.0532 1 186 192 3 139.457 193.316 62.035 1 191 194 3 137.575 193.674 59.7969 1 193 204 3 140.204 190.642 57.3389 1 203 205 3 127.753 180.679 52.6345 1 204 205 3 126.928 176.931 57.8752 1 204

127 3 131.679 217.437 66.6041 1 126 173 -4 141.267 187.523 68.5978 1 172 173 -5 137.171 193.537 70.845 1 172 182 3 135.361 190.114 66.1693 1 181 188 3 136.214 188.623 67.4983 1 187 193 3 134.28

192.457 64.965 1 192 195 3 132.398 192.815 62.7269 1 194 205 3 133.581 189.792 58.7962 1 204 206 3 121.858 178.345 55.2744 1 205 206 3 120.336 174.475 58.6207 1 205

128 3 126.793 218.131 67.409 1 127 174 -4 137.309 186.037 71.2668 1 173 174 -5 132.827 192.548 71.58 1 173 183 3 131.303 189.665 67.6426 1 182 189 3 132.522 187.084 68.505 1 188 194 3 130.588 190.918 65.9717 1 193 196 3 129.216 190.865 64.3067 1 195 206 3 130.399 187.842 60.376 1 205 207 3 117.416 176.386 56.6807 1 206 207 3 115.894 172.516 60.027 1 206

129 3 121.907 218.825 68.2138 1 128 175 -4 133.351 184.55 73.9359 1 174 175 -5 128.249 191.364 72.166 1 174 184 3 127.201 189.382 69.459 1 183 190 3 128.519 186.544 70.9473 1 189 195 3 126.561 189.174 69.1472 1 194 197 3 124.679 189.532 66.9091 1 196 207 3 124.419 185.027 63.9856 1 206 208 3 111.77 174.787 58.9004 1 207 208 3 110.248 170.917 62.2467 1 207

130 3 117.116 219.024 69.6292 1 129 176 -4 129.393 183.064 76.6049 1 175 176 -5 123.637 191.14 73.6694 1 175 185 3 123.396 187.64 72.0007 1 184 191 3 124.709 184.901 73.2775 1 190 196 3 122.751 187.531 71.4774 1 195 198 3 120.869 187.889 69.2393 1 197 208 3 120.609 183.384 66.3158 1 207 209 3 107.933 172.805 61.0757 1 208 209 3 105.171 168.511 63.8649 1 208

131 3 112.205 219.728 70.2501 1 130 177 -4 125.212 182.046 79.152 1 176 177 -5 115.882 189.242 75.5195 1 176 186 3 116.922 185.577 75.1348 1 185 192 3 118.235 182.838 76.4116 1 191 197 3 117.617 185.267 74.7833 1 196 199 3 115.735 185.625 72.5452 1 198 209 3 116.2 179.424 69.8366 1 208 210 3 102.995 169.909 63.8134 1 209 210 3 99.9656 165.255 65.9399 1 209

132 3 107.375 220.085 71.4948 1 131 178 -4 121.428 180.347 81.943 1 177 178 -5 112.098 187.543 78.3105 1 177 187 3 112.498 184.674 77.0625 1 186 193 3 113.811 181.935 78.3393 1 192 198 3 113.193 184.364 76.711 1 197 200 3 111.463 184.107 73.824 1 199 210 3 112.229 176.631 72.5125 1 209 211 3 99.0239 167.116 66.4893 1 210 211 3 95.4042 162.003 68.3301 1 210

133 3 102.471 220.62 72.3036 1 132 179 -4 118.096 178.841 85.354 1 178 179 -5 107.936 185.509 80.9368 1 178 188 3 108.192 182.631 79.4629 1 187 194 3 108.86 179.106 80.6437 1 193 199 3 108.433 182.943 79.716 1 198 201 3 106.865 182.325 76.4301 1 200 211 3 107.606 174.296 74.2419 1 210 212 3 94.4009 164.781 68.2187 1 211 212 3 92.0778 159.292 70.659 1 211

134 3 96.0862 221.191 73.7604 1 133 180 -4 114.765 177.336 88.765 1 179 180 -5 104.121 183.863 84.0529 1 179 189 3 104.293 181.074 81.991 1 188 195 3 104.961 177.549 83.1718 1 194 200 3 104.534 181.386 82.2441 1 199 202 3 102.966 180.768 78.9582 1 201 212 3 103.075 172.273 76.075 1 211 213 3 90.8822 162.092 70.7176 1 212 213 3 88.379 156.269 72.1948 1 212

135 3 92.0733 219.815 75.5409 1 134 181 -4 111.433 175.83 92.176 1 180 181 -5 100.452 181.267 85.8172 1 180 190 3 101.311 177.803 84.0967 1 189 196 3 101.33 174.665 84.9977 1 195 201 3 100.673 179.447 85.2867 1 200 203 3 98.83 179.463 81.4611 1 202 213 3 98.7813 170.877 78.6953 1 212 214 3 86.5269 159.333 73.6055 1 213 214 3 83.729 153.577 74.3254 1 213

136 3 88.9194 218.817 77.5491 1 135 182 -4 106.582 174.94 94.2591 1 181 182 -5 95.5683 179.449 88.9762 1 181 191 3 95.9904 176.203 88.0642 1 190 197 3 96.2407 172.016 89.6032 1 196 202 3 94.3301 177.684 87.735 1 201 204 3 92.4478 178.042 85.4969 1 203 214 3 92.3991 169.456 82.7311 1 213 215 3 80.941 156.989 78.6886 1 214 215 3 77.3317 151.003 78.5332 1 214

137 3 84.6331 217.504 79.7635 1 136 183 -4 103.724 175.613 96.5291 1 182 183 -5 91.4488 177.232 91.2465 1 182 192 3 91.5981 174.486 90.3279 1 191 198 3 92.1485 170.459 92.4581 1 197 203 3 90.2379 176.127 90.5899 1 202 205 3 88.3556 176.485 88.3518 1 204 215 3 85.7501 167.66 85.7889 1 214 216 3 75.6056 155.456 82.6649 1 215 216 3 71.6554 148.715 81.4343 1 215

138 3 80.3469 216.191 81.9779 1 137 184 -4 99.1146 174.898 100.308 1 183 184 -5 87.0203 175.517 94.1595 1 183 193 3 86.927 172.856 93.3189 1 192 199 3 87.3029 168.737 95.5624 1 198 204 3 85.1898 175.227 93.63 1 203 206 3 84.1695 176.015 92.3837 1 205 216 3 80.5833 164.599 88.599 1 215 217 3 69.6977 153.339 85.4442 1 216 217 3 65.6857 145.375 84.6129 1 216

139 3 73.4832 213.869 85.5988 1 138 185 -4 95.464 174.847 103.724 1 184 185 -5 82.0724 173.223 97.0968 1 184 194 3 81.2834 171.302 95.3234 1 193 200 3 82.4237 166.727 97.8739 1 199 205 3 78.1478 173.477 98.523 1 204 207 3 77.1275 174.265 97.2767 1 206 217 3 72.9898 162.643 93.2492 1 216 218 3 65.1681 150.719 88.2069 1 217 218 3 61.0568 142.083 83.0643 1 217

0 0 X X X X X 186 -4 92.1525 174.913 107.47 1 185 186 -5 77.2882 171.47 99.6558 1 185 195 3 76.4992 169.549 97.8824 1 194 201 3 77.2124 165.374 100.926 1 200 206 3 71.5998 172.228 101.497 1 205 208 3 70.4282 172.156 100.665 1 207 218 3 69.9411 160.315 88.4638 1 217 219 3 61.9994 149.882 91.3448 1 218 219 3 57.6221 139.788 81.107 1 218

0 0 X X X X X 187 -4 88.0972 175.064 111.033 1 186 187 -5 70.6532 169.921 103.627 1 186 196 3 71.3957 166.18 101.335 1 195 202 3 72.9287 163.168 102.674 1 201 207 3 67.0762 167.946 104.984 1 206 209 3 65.0811 169.186 103.558 1 208 219 3 67.4067 158.703 83.5771 1 218 220 3 59.4075 148.208 83.0875 1 219 220 3 55.134 139.133 75.007 1 219

0 0 X X X X X 0 0 X X X X X 0 0 X X X X X 0 0 X X X X X 203 -1 70 160.333 104 1 202 208 3 65.7907 165.33 101.128 1 207 210 3 63.6173 168.559 106.134 1 209 220 3 64.862 157.802 77.2968 1 219 221 3 55.1712 146.078 74.9152 1 220 221 3 52.7195 137.994 66.5025 1 220

0 0 X X X X X 0 0 X X X X X 0 0 X X X X X 0 0 X X X X X 204 -1 67.5711 158.051 104.384 1 203 209 3 64.8743 164.797 98.428 1 208 211 3 63.2075 170.119 109.061 1 210 221 3 60.6592 158.342 71.7851 1 220 222 3 51.6702 147.447 68.7381 1 221 222 3 49.6408 140.437 58.7691 1 221

0 0 X X X X X 0 0 X X X X X 0 0 X X X X X 0 0 X X X X X 205 -1 64 155 105.5 1 204 210 3 63.7135 164.234 95.2534 1 209 212 3 62.2981 170.188 112.528 1 211 222 3 54.4693 159.385 67.6741 1 221 223 3 48.0417 149.483 62.1977 1 222 223 3 43.8485 142.693 54.6488 1 222

0 0 X X X X X 0 0 X X X X X 0 0 X X X X X 0 0 X X X X X 0 0 X X X X X 211 -1 62.7694 162.31 92.5716 1 210 213 -4 60.5205 169.752 116.069 1 212 223 -5 48.9856 158.997 64.3629 1 222 224 3 42.1908 151.277 57.4845 1 223 224 3 37.7153 145.057 50.055 1 223

0 0 X X X X X 0 0 X X X X X 0 0 X X X X X 0 0 X X X X X 0 0 X X X X X 212 -1 61.287 160.903 87.2091 1 211 214 -4 58.6691 170.068 117.793 1 213 224 -5 41.8932 158.727 61.4753 1 223 225 3 36.4841 153.722 53.7421 1 224 225 3 32.7817 148.225 45.8121 1 224

0 0 X X X X X 188 -4 103 173 95.75 1 182 188 0 X X X X X 197 0 X X X X X 206 0 X X X X X 213 0 X X X X X 215 0 X X X X X 225 0 X X X X X 226 0 X X X X X 226 0 X X X X X

140 3 91.9677 223.555 76.2068 1 134 189 -4 100.799 171.079 100.22 1 188 189 0 X X X X X 198 0 X X X X X 207 0 X X X X X 214 0 X X X X X 216 0 X X X X X 226 0 X X X X X 227 0 X X X X X 227 0 X X X X X

141 3 88.4325 226.168 78.5889 1 140 190 -4 98.6307 169.392 103.604 1 189 190 0 X X X X X 199 0 X X X X X 208 0 X X X X X 215 0 X X X X X 217 0 X X X X X 227 0 X X X X X 228 0 X X X X X 228 0 X X X X X

142 3 84.9907 228.905 80.9689 1 141 191 -4 94.6228 168.093 107.314 1 190 191 0 X X X X X 200 0 X X X X X 209 0 X X X X X 216 0 X X X X X 218 0 X X X X X 228 0 X X X X X 229 0 X X X X X 229 0 X X X X X

143 3 81.5583 231.683 83.3137 1 142 192 -4 91 168 110 1 191 192 0 X X X X X 201 0 X X X X X 210 0 X X X X X 217 0 X X X X X 219 0 X X X X X 229 0 X X X X X 230 0 X X X X X 230 0 X X X X X

144 3 78.0251 234.302 85.6932 1 143 193 -4 111.226 174.009 97.0811 1 181 193 0 X X X X X 202 0 X X X X X 211 0 X X X X X 218 0 X X X X X 220 0 X X X X X 230 0 X X X X X 231 0 X X X X X 231 0 X X X X X

145 3 72.6562 237.314 89.0246 1 144 194 -4 111.75 171.875 101.572 1 193 194 0 X X X X X 203 0 X X X X X 212 0 X X X X X 219 0 X X X X X 221 0 X X X X X 231 0 X X X X X 232 0 X X X X X 232 0 X X X X X

146 3 160.983 200.949 52.5344 1 119 195 -4 111.969 170.313 106.317 1 194 195 0 X X X X X 204 0 X X X X X 213 0 X X X X X 220 0 X X X X X 222 0 X X X X X 232 0 X X X X X 233 0 X X X X X 233 0 X X X X X

147 3 158.581 198.976 54.878 1 146 196 -4 112.398 168.966 111.113 1 195 196 0 X X X X X 205 0 X X X X X 214 0 X X X X X 221 0 X X X X X 223 0 X X X X X 233 0 X X X X X 234 0 X X X X X 234 0 X X X X X

148 3 155.257 196.18 57.3537 1 147 197 -4 112.827 167.62 115.909 1 196 197 0 X X X X X 206 0 X X X X X 215 0 X X X X X 222 0 X X X X X 224 0 X X X X X 234 0 X X X X X 235 0 X X X X X 235 0 X X X X X

149 3 151.914 194.042 60.396 1 148 198 -4 113.051 166.821 120.84 1 197 198 0 X X X X X 207 0 X X X X X 216 0 X X X X X 223 0 X X X X X 225 0 X X X X X 235 0 X X X X X 236 0 X X X X X 236 0 X X X X X

150 3 148.613 191.508 63.1682 1 149 199 -4 113.009 165.412 125.637 1 198 199 0 X X X X X 208 0 X X X X X 217 0 X X X X X 224 0 X X X X X 226 0 X X X X X 236 0 X X X X X 237 0 X X X X X 237 0 X X X X X

151 3 144.805 189.313 65.5521 1 150 200 -4 112.97 163.759 128.424 1 199 200 0 X X X X X 209 0 X X X X X 218 0 X X X X X 225 0 X X X X X 227 0 X X X X X 237 0 X X X X X 238 0 X X X X X 238 0 X X X X X

152 3 141.267 187.523 68.5978 1 151 201 -4 113.098 163.738 131.635 1 200 201 0 X X X X X 210 0 X X X X X 219 0 X X X X X 226 0 X X X X X 228 0 X X X X X 238 0 X X X X X 239 0 X X X X X 239 0 X X X X X

153 3 137.309 186.037 71.2668 1 152 202 -4 113.044 162.822 134.757 1 201 202 0 X X X X X 211 0 X X X X X 220 0 X X X X X 227 0 X X X X X 229 0 X X X X X 239 0 X X X X X 240 0 X X X X X 240 0 X X X X X

154 3 133.351 184.55 73.9359 1 153 203 3 180.966 208.332 54.3266 1 133 203 3 175.537 211.889 59.204 1 133 212 3 181.38 210.388 54.6924 1 137 221 3 177.779 208.648 53.8387 1 143 228 3 176.142 212.833 54.6516 1 146 230 3 180.464 212.33 50.3107 1 148 240 3 177.502 209.973 51.3707 1 158 241 3 175.485 207.103 50.7851 1 158 241 3 172.096 205.352 53.8018 1 158

155 3 129.393 183.064 76.6049 1 154 204 3 177.029 208.562 63.7418 1 203 204 3 174.435 212.052 63.7012 1 203 213 3 179.257 210.958 61.0897 1 212 222 3 175.577 208.33 60.0574 1 221 229 3 173.94 212.515 60.8703 1 228 231 3 177.81 212.361 58.2475 1 230 241 3 174.848 210.004 59.3075 1 240 242 3 172.831 207.134 58.7219 1 241 242 3 169.062 204.717 61.7173 1 241

156 3 125.212 182.046 79.152 1 155 205 3 175.734 208.384 68.75 1 204 205 3 173.066 212.843 68.7506 1 204 214 3 176.72 210.839 65.9171 1 213 223 3 173.727 208.235 64.8439 1 222 230 3 171.835 212.301 65.9111 1 229 232 3 174.516 212.689 67.7644 1 231 242 3 173.875 209.585 66.7196 1 241 243 3 169.442 206.127 67.4922 1 242 243 3 165.673 203.71 70.4876 1 242

157 3 121.428 180.347 81.943 1 156 206 3 174.012 209.11 73.3878 1 205 206 3 172.665 213.346 73.4342 1 205 215 3 175.554 211.138 71.0253 1 214 224 3 171.387 209.814 70.8597 1 223 231 3 170.894 213.566 71.999 1 230 233 3 171.925 212.702 73.3507 1 232 243 3 173.306 209.5 72.9009 1 242 244 3 166.987 206.253 72.5284 1 243 244 3 163.218 203.836 75.5238 1 243

158 3 118.096 178.841 85.354 1 157 207 3 172.681 209.042 77.8231 1 206 207 3 171.619 213.121 78.1176 1 206 216 3 171.177 212.4 78.192 1 215 225 3 169.613 208.788 78.2189 1 224 232 3 169.396 215.653 78.5138 1 231 234 3 169.887 214.236 79.6579 1 233 244 3 171.661 211.539 79.5302 1 243 245 3 162.275 206.543 79.0387 1 244 245 3 159.368 205.663 82.1589 1 244

159 3 114.765 177.336 88.765 1 158 208 3 171.012 209.993 82.4395 1 207 208 3 170.343 213.835 82.9066 1 207 217 3 169.901 213.114 82.981 1 216 226 3 168.081 210.151 83.0184 1 225 233 3 168.872 215.963 84.0441 1 232 235 3 167.701 214.853 83.8793 1 234 245 3 170.517 211.069 85.2433 1 244 246 3 160.895 205.39 85.9814 1 245 246 3 157.988 204.51 89.1016 1 245

160 3 111.433 175.83 92.176 1 159 209 3 170.286 210.154 87.5451 1 208 209 3 169.167 214.247 87.8031 1 208 218 3 168.725 213.526 87.8775 1 217 227 3 166.266 211.012 87.8359 1 226 234 3 167.057 216.824 88.8616 1 233 236 3 165.886 215.714 88.6968 1 235 246 3 170.758 212.207 90.9349 1 245 247 3 158.05 205.44 93.8856 1 246 247 3 155.143 204.56 97.0058 1 246

161 3 106.582 174.94 94.2591 1 160 210 3 168.945 210.683 92.2495 1 209 210 3 167.826 214.776 92.5075 1 209 219 3 167.826 214.776 92.5075 1 218 228 3 165.367 212.262 92.4659 1 227 235 3 166.158 218.074 93.4916 1 234 237 3 164.987 216.964 93.3268 1 236 247 3 170.267 212.475 98.6885 1 246 248 3 156.573 205.8 101.912 1 247 248 3 153.666 204.92 105.032 1 247

162 3 103.724 175.613 96.5291 1 161 211 3 167.895 211.401 97.0849 1 210 211 3 166.341 214.864 96.7365 1 210 220 3 166.341 214.864 96.7365 1 219 229 3 164.98 212.991 96.6601 1 228 236 3 165.771 218.803 97.6858 1 235 238 3 164.6 217.693 97.521 1 237 248 3 169.494 213.488 102.68 1 247 249 3 153.909 205.801 105.293 1 248 249 3 151.42 205.164 109.604 1 248

163 3 99.1146 174.898 100.308 1 162 212 3 167.023 212.247 101.607 1 211 212 3 165.469 215.71 101.259 1 211 221 3 166.528 216.017 101.443 1 220 230 3 164.179 213.136 102.06 1 229 237 3 164.97 218.948 103.086 1 236 239 3 163.434 217.772 103.773 1 238 249 3 168.04 214.127 107.57 1 248 250 3 152.455 206.44 110.183 1 249 250 3 149.882 205.754 114.256 1 249

164 3 95.464 174.847 103.724 1 163 213 3 165.835 213.417 107.682 1 212 213 3 164.022 217.286 107.348 1 212 222 3 165.081 217.593 107.532 1 221 231 3 163.404 213.663 107.947 1 230 238 3 164.195 219.475 108.973 1 237 240 3 162.659 218.299 109.66 1 239 250 3 166.94 214.121 112.793 1 249 251 3 150.42 206.029 114.617 1 250 251 3 148.813 205.961 118.78 1 250

165 3 92.1525 174.913 107.47 1 164 214 -1 165.32 213.158 113.084 0.654809 213 214 3 162.095 217.166 112.923 1.15481 213 223 3 163.792 217.394 113.237 1.15481 222 232 3 163.192 214.012 113.766 1.15481 231 239 3 162.27 220.289 113.853 1.15481 238 241 3 161.191 219.184 113.438

1.15481 240 251 3 167.157 214.312 117.079 1.15481 250 252 3 150.637 206.22 118.903 1.15481 251 252 3 147.73 205.34 122.023 1.15481 251

166 3 88.0972 175.064 111.033 1 165 215 -1 164.116 213.565 117.92 0.67015 214 215 3 160.435 218.598 117.741 1.17015 214 224 3 162.994 216.62 118.471 1.17015 223 233 3 163.958 213.584 119.428 1.17015 232 240 3 161.473 220.255 120.175 1.17015 239 242 3 160.394 219.15 119.76 1.17015 241 252 3 167.003 213.864 123.013 1.17015 251 253 3 150.483 205.772 124.837 1.17015 252 253 3 147.576 204.892 127.957 1.17015 252

167 3 103 173 95.75 1 161 216 -1 164.064 214.082 122.893 1.02689 215 216 3 160.723 218.021 122.374 1.02689 215 225 3 162.677 216.646 121.972 1.02689 224 234 3 162.44 214.261 123.951 1.02689 233 241 3 160.233 220.851 125.104 1.02689 240 243 3 159.154 219.746 124.689 1.02689 242 253 3 163.926 212.48 128.232 1.02689 252 254 3 151.386 203.352 131.536 1.02689 253 254 3 147.36 201.631 133.883 1.02689 253

168 3 100.799 171.079 100.22 1 167 217 -1 164.063 214.04 127.893 0.960455 216 217 3 159.466 217.245 127.31 0.960455 216 226 3 161.42 215.87 126.908 0.960455 225 235 3 161.823 212.416 127.846 0.960455 234 242 3 159.616 219.006 128.999 0.960455 241 244 3 159.616 219.006 128.999 0.960455 243 254 3 162.062 212.205 133.862 0.960455 253 255 3 150.942 201.518 136.168 0.960455 254 255 3 146.204 198.864 138.593 0.960455 254

169 3 98.6307 169.392 103.604 1 168 218 -1 164.058 213.572 132.871 0.889491 217 218 3 159.461 216.777 132.288 0.889491 217 227 3 161.274 215.242 131.712 0.889491 226 236 3 162.192 211.615 132.968 0.889491 235 243 3 159.985 218.205 134.121 0.889491 242 245 3 158.267 216.704 133.437 0.889491 244 255 3 159.977 210.166 137.224 0.889491 254 256 3 150.874 199.053 140.566 0.889491 255 256 3 145.582 195.909 142.715 0.889491 255

170 3 94.6228 168.093 107.314 1 169 219 -1 164.052 213.104 137.849 0.889491 218 219 3 159.455 216.309 137.266 0.889491 218 228 3 161.268 214.774 136.69 0.889491 227 237 3 161.04 210.616 137.2 0.889491 236 244 3 159.881 216.923 138.845 0.889491 243 246 3 158.163 215.422 138.161 0.889491 245 256 3 157.774 209.413 140.42 0.889491 255 257 3 151.525 197.239 146.081 0.889491 256 257 3 144.231 193.121 147.114 0.889491 256

171 3 91 168 110 1 170 220 -1 164.026 212.121 142.751 0.574652 219 220 3 158.671 214.475 142.055 1.07465 219 229 3 161.811 212.694 141.474 1.07465 228 238 3 159.727 209.241 141.922 1.07465 237 245 3 159.479 216.216 143.258 1.07465 244 247 3 158.708 213.99 142.561 1.07465 246 257 3 155.597 204.674 144.691 1.07465 256 258 3 149.327 193.334 150.526 1.07465 257 258 3 142.629 189.678 151.814 1.07465 257

172 3 111.226 174.009 97.0811 1 160 221 -1 163.806 211.339 147.685 0.513807 220 221 3 158.545 213.651 147.527 1.01381 220 230 3 161.696 209.752 146.927 1.01381 229 239 3 158.809 207.117 147.389 1.01381 238 246 3 159.595 214.62 149.217 1.01381 245 248 3 158.438 212.299 148.328 1.01381 247 258 3 151.738 202.013 150.358 1.01381 257 259 3 147.137 187.973 155.443 1.01381 258 259 3 141.193 185.226 156.631 1.01381 258

173 3 111.75 171.875 101.572 1 172 222 -1 163.163 209.869 152.42 1 221 222 3 156.75 211.937 153.643 1 221 231 3 159.301 205.849 152.539 1 230 240 3 157.244 204.414 152.03 1 239 247 3 159.074

213.338 154.35 1 246 249 3 157.009 210.981 153.097 1 248 259 3 149.964 199.747 154.571 1 258 260 3 142.846 183.522 159.673 1 259 260 3 135.927 181.842 161.504 1 259

174 3 111.969 170.313 106.317 1 173 223 -1 163.033 208.015 157.062 0.759509 222 223 3 156.014 210.113 158.43 0.759509 222 232 3 156.595 202.698 157.335 0.759509 231 241 3 155.844 202.297 156.957 0.759509 240 248 3 157.674 211.221 159.277 0.759509 247 250 3 156.01 209.075 159.067 0.759509 249 260 3 148.022 196.822 158.786 0.759509 259 261 3 139.048 180.549 162.06 0.759509 260 261 3 132.952 180.052 164.265 0.759509 260

175 3 112.398 168.966 111.113 1 174 224 -1 163.088 205.999 161.637 0.759509 223 224 3 153.208 208.921 163.54 0.759509 223 233 3 154.139 202.826 159.827 0.759509 232 242 3 153.848 199.639 159.936 0.759509 241 249 3 157.26 209.534 163.116 0.759509 248 251 3 155.596 207.388 162.906 0.759509 250 261 3 146.174 194.582 162.321 0.759509 260 262 3 136.177 179.266 164.494 0.759509 261 262 3 130.277 178.609 168.225 0.759509 261

176 3 112.827 167.62 115.909 1 175 225 -1 163.143 203.984 166.213 0.759509 224 225 3 152.538 207.147 168.531 0.759509 224 234 3 152.524 200.647 163.173 0.759509 233 243 3 151.36 198.241 164.057 0.759509 242 250 3 156.218 206.877 167.546 0.759509 249 252 3 155.061 204.556 166.657 0.759509 251 262 3 143.022 191.967 165.432 0.759509 261 263 3 132.463 176.321 166.928 0.759509 262 263 3 127.364 176.169 170.589 0.759509 262

177 3 113.051 166.821 120.84 1 176 226 -1 163.198 201.968 170.788 0.616847 225 226 3 151.908 205.231 172.396 0.616847 225 235 3 151.564 200.332 167.451 0.616847 234 244 3 149.793 197.041 167.994 0.616847 243 251 3 154.816 205.464 170.887 0.616847 250 253 3 152.937 203.741 170.075 0.616847 252 263 3 138.38 192.031 169.682 0.616847 262 264 3 127.561 176.407 170.079 0.616847 263 264 3 124.654 175.527 173.199 0.616847 263

178 3 113.009 165.412 125.637 1 177 227 -1 162.526 200.54 175.532 0.872774 226 227 3 151.236 203.803 177.14 0.872774 226 236 3 150.592 199.891 171.042 0.872774 235 245 3 149.181 195.392 171.77 0.872774 244 252 3 152.275 203.066 176.82 0.872774 251 254 3 149.656 200.606 175.367 0.872774 253 264 3 134.491 192.32 173.753 0.872774 263 265 3 122.043 175.644 172.421 0.872774 264 265 3 120.524 173.56 177.186 0.872774 264

179 3 112.97 163.759 128.424 1 178 228 -1 162.048 199.654 180.43 0.872774 227 228 3 150.758 202.917 182.038 0.872774 227 237 -4 151.319 196.833 175.427 0.872774 236 246 -5 146.365 193.251 175.105 0.872774 245 253 3 149.173 202.389 179.178 0.872774 252 255 3 146.554 199.929 177.725 0.872774 254 265 3 129.677 192.215 177.259 0.872774 264 266 3 116.201 175.97 174.409 0.872774 265 266 3 115.318 170.848 180.809 0.872774 265

180 3 113.098 163.738 131.635 1 179 229 -1 161.57 198.769 185.328 0.902118 228 229 3 149.287 201.512 186.573 0.902118 228 238 -4 149.848 195.428 179.962 0.902118 237 247 -5 143.379 192.781 177.012 0.902118 246 254 3 147.495 202.487 182.04 0.902118 253 256 3 144.876 200.027 180.587 0.902118 255 266 3 123.415 190.567 179.507 0.902118 265 267 3 110.963 173.351 177.677 0.902118 266 267 3 109.206 171.839 181.606 0.902118 266

181 3 113.044 162.822 134.757 1 180 230 -1 160.551 197.933 190.151 0.902118 229 230 3 147.502 200.057 191.293 0.902118 229 239 -4 148.063 193.973 184.682 0.902118 238 248 -5 139.233 192.651 179.374 0.902118 247 255 3 144.886 202.599 185.084 0.902118 254 257 3 142.356 198.173 184.786

0.902118 256 267 3 118.849 187.846 182.668 0.902118 266 268 3 107.857 168.763 179.02 0.902118 267 268 3 103.653 170.106 184.281 0.902118 267

182 3 180.966 208.332 54.3266 1 115 231 -1 159.532 197.097 194.974 1.1238 230 231 3 146.154 198.792 195.976 1.1238 230 240 -4 146.715 192.708 189.365 1.1238 239 249 -5 135.956 192.884 180.503 1.1238 248 256 3 142.743 202.177 189.116 1.1238 255 258 3 139.521 197.021 187.998 1.1238 257 268 3 116.604 182.776 184.578 0.6238 267 269 3 107.2 163.702 180.994 1.1238 268 269 3 102.429 164.193 185.445 1.1238 268

183 3 177.215 209.093 63.5652 1 182 232 -1 158.048 196.135 199.651 1.00521 231 232 3 146.491 197.305 200.312 1.00521 231 241 -4 147.052 191.221 193.701 1.00521 240 250 0 X X X X X 257 -5 140.06 202.591 192.799 1.00521 256 259 3 136.64 196.801 191.803 1.00521 258 269 3 116.231 180.906 187.044 0.50521 268 270 3 107.946 159.671 182.524 1.00521 269 270 3 100.958 160.326 186.761 1.00521 269

184 3 175.34 209.474 68.1845 1 183 233 -1 155.505 195 203.804 1.04698 232 233 3 145.907 196.065 203.835 1.04698 232 242 -4 146.468 189.981 197.224 1.04698 241 251 0 X X X X X 258 -5 137.736 201.779 195.209 1.04698 257 260 3 133.993 195.788 194.594 1.04698 259 270 3 115.109 178.044 190.298 0.54698 269 271 3 107.732 155.074 183.859 1.04698 270 271 3 100.252 156.786 190.717 1.04698 270

185 3 173.618 210.2 72.8223 1 184 234 -1 151.573 192.215 211.405 1.04698 233 234 3 145.814 194.401 208.201 1.04698 233 243 -4 146.375 188.317 201.59 1.04698 242 252 0 X X X X X 259 -5 134.83 203.028 198.968 1.04698 258 261 3 131.023 194.768 198.743 1.04698 260 271 -4 114.498 175.762 193.434 1.04698 270 272 0 X X X X X 272 -5 99.934 155.574 193.19 1.04698 271

0 0 X X X X X 0 0 X X X X X 0 0 X X X X X 0 0 X X X X X 0 0 X X X X X 0 0 X X X X X 262 -1 128.234 193.715 200.841 1 261 272 -4 112.683 174.068 195.85 1 271 273 0 X X X X X 273 0 X X X X X

0 0 X X X X X 0 0 X X X X X 0 0 X X X X X 0 0 X X X X X 0 0 X X X X X 0 0 X X X X X 263 -1 126.234 193.715 203.341 1.5 262 273 -4 110.683 174.068 198.35 1 272 274 0 X X X X X 274 0 X X X X X

186 3 172.305 210.893 77.5968 1 185 235 3 193.258 201.9 45.517 1 129 235 3 189.9 206.564 47.6614 1 129 244 3 189.908 204.004 45.8391 1 133 253 3 190.918 203.146 44.0513 1 139 260 3 190.113 205.44 44.9 1 142 264 3 190.113 205.44 44.9 1 144 274 3 192.492 202.762 37.9663 1 154 275 3 187.233 199.913 38.341 1 154 275 3 184.049 199.256 43.8848 1 154

187 3 170.636 211.844 82.2132 1 186 236 3 190.611 199.67 49.3106 1 235 236 3 186.868 204.293 51.7318 1 235 245 3 186.405 201.449 50.069 1 244 254 3 186.998 201.56 48.675 1 253 261 3 186.193 203.854 49.5237 1 260 265 3 185.738 202.977 47.0669 1 264 275 3 185.635 199.845 45.0893 1 274 276 3 180.646 194.164 45.5102 1 275 276 3 177.992 193.627 48.94 1 275

188 3 169.11 212.738 86.8898 1 187 237 3 188.213 198.111 53.4119 1 236 237 3 183.585 203.977 54.3047 1 236 246 3 184.094 200.121 52.7312 1 245 255 3 184.094 200.121 52.7312 1 254 262 3 183.289 202.415 53.5799 1 261 266 3 182.245 200.699 50.3475 1 265 276 3 182.051 197.408 50.6383 1 275 277 3 177.521 190.597 51.0249 1 276 277 3 174.196 189.015 53.751 1 276

189 3 168.247 213.25 91.7881 1 188 238 3 185.876 196.798 57.5113 1 237 238 3 180.183 202.454 57.9158 1 237 247 3 181.108 199.676 57.5973 1 246 256 3 180.787 198.3 56.4476 1 255 263 3 180.21

200.553 56.5325 1 262 267 3 179.828 199.212 55.2979 1 266 277 3 179.839 194.835 55.0381 1 276 278 3 174.85 189.154 55.459 1 277 278 3 171.571 186.322 57.8411 1 277

190 3 167.197 213.968 96.6235 1 189 239 3 183.824 193.57 61.8395 1 238 239 3 176.298 200.846 61.168 1 238 248 3 179.708 197.74 61.6537 1 247 257 3 178.515 196.933 59.8984 1 256 264 3 177.938 199.186 59.9833 1 263 268 3 178.012 197.57 60.4936 1 267 278 3 177.301 192.222 59.15 1 277 279 3 172.312 186.541 59.5709 1 278 279 3 169.49 182.261 61.9804 1 278

191 3 165.915 215.042 101.336 1 190 240 3 182.368 191.839 66.2984 1 239 240 3 175.733 199.128 68.1135 1 239 249 3 177.746 195.73 67.908 1 248 258 3 176.553 194.923 66.1527 1 257 265 3 176.198 195.898 66.489 1 264 269 3 176.272 194.282 66.9993 1 268 279 3 175.561 188.934 65.6557 1 278 280 3 169.838 181.483 64.8442 1 279 280 3 167.947 177.077 67.409 1 279

192 3 164.727 216.212 107.411 1 191 241 3 181.117 189.953 70.7572 1 240 241 3 176.274 197.705 73.8679 1 240 250 3 176.171 193.433 72.8719 1 249 259 3 174.72 192.569 72.5031 1 258 266 3 174.365 193.544 72.8394 1 265 270 3 175.217 191.557 71.6601 1 269 280 3 174.506 186.209 70.3165 1 279 281 3 168.654 178.647 69.1061 1 280 281 3 166.897 173.293 71.0229 1 280

0 0 X X X X X 242 3 179.867 188.067 75.2161 1 241 242 3 175.024 195.819 78.3268 1 241 251 3 176 191.432 78.0153 1 250 260 3 173.472 190.16 76.3571 1 259 267 3 173.119 191.705 76.4574 1 266 271 3 174.203 188.754 75.0846 1 270 281 3 173.492 183.406 73.741 1 280 282 3 167.252 175.429 72.3002 1 281 282 3 165.726 170.109 74.8449 1 281

0 0 X X X X X 243 3 179.17 185.59 79.8548 1 242 243 3 174.081 193.361 81.9257 1 242 252 3 176.323 188.054 80.7092 1 251 261 3 173.795 186.782 79.051 1 260 268 3 172.66 190.472 79.8911 1 267 272 3 173.305 186.356 79.3825 1 271 282 3 172.594 181.008 78.0389 1 281 283 3 164.245 173.494 76.829 1 282 283 3 162.931 168.635 79.1824 1 282

0 0 X X X X X 244 3 178.425 183.711 84.4277 1 243 244 3 172.572 191.135 85.9753 1 243 253 3 174.814 185.828 84.7588 1 252 262 3 171.635 184.351 81.3885 1 261 269 3 172.83 188.9 84.0871 1 268 273 3 172.475 185.738 83.8485 1 272 283 3 172.227 179.269 81.8744 1 282 284 3 162.264 171.208 81.0383 1 283 284 3 160.95 166.349 83.3917 1 283

0 0 X X X X X 245 3 177.328 182.936 89.0655 1 244 245 3 171.171 188.07 91.4201 1 244 254 3 172.689 183.565 90.7683 1 253 263 3 170.822 182.632 85.2876 1 262 270 3 171.693 187.811 88.1208 1 269 274 3 170.655 184.392 88.5389 1 273 284 3 170.925 176.387 86.2216 1 283 285 3 160.828 168.984 86.1903 1 284 285 3 159.181 163.168 88.3441 1 284

0 0 X X X X X 246 3 176.688 181.739 93.7015 1 245 246 3 170.531 186.873 96.0561 1 245 255 3 172.049 182.368 95.4043 1 254 264 3 169.445 180.749 91.7305 1 263 271 3 169.424 184.68 95.9145 1 270 275 3 168.826 182.427 95.4685 1 274 285 3 169.096 174.422 93.1512 1 284 286 3 158.483 165.697 91.9596 1 285 286 3 156.836 159.881 94.1134 1 285

0 0 X X X X X 247 3 175.637 180.734 98.1311 1 246 247 3 169.48 185.868 100.486 1 246 256 3 171.722 180.561 99.2695 1 255 265 3 168.634 177.616 97.3227 1 264 272 3 168.101 182.66 101.501 1 271 276 3 167.503 180.407 101.055 1 275 286 3 167.016 171.591 98.2236 1 285 287 3 156.403 162.866 97.032 1 286 287 3 154.411 156.621 98.8235 1 286

0 0 X X X X X 248 3 174.727 180.099 103.006 1 247 248 3 168.57 185.233 105.361 1 247 257 3 170.812 179.926 104.145 1 256 266 3 167.063 174.744 101.609 1 265 273 3 166.041 180.772 106.242 1 272 277 3 164.037 176.233 105.819 1 276 287 3 165.457 169 105.389 1 286 288 3 153.832 158.856 103.254 1 287 288 3 152.385 152.433 105.246 1 287

0 0 X X X X X 249 3 173.932 178.457 107.661 1 248 249 3 165.819 182.566 112.145 1 248 258 3 168.061 177.259 110.929 1 257 267 3 165.928 173.305 109.637 1 266 274 3 165.395 178.349 113.815 1 273 278 3 161.478 173.585 112.115 1 277 288 3 165.075 165.489 110.161 1 287 289 3 151.709 155.833 109.42 1 288 289 3 150.618 148.3 111.501 1 288

0 0 X X X X X 250 3 173.137 176.814 112.316 1 249 250 3 164.495 179.719 115.9 1 249 259 3 166.737 174.412 114.684 1 258 268 3 165.02 171.188 113.467 1 267 275 3 164.487 176.232 117.645 1 274 279 3 159.087 171.476 115.762 1 278 289 3 165.2 162.805 115.393 1 288 290 3 150.976 151.683 114.611 1 289 290 3 149.603 144.876 116.616 1 289

0 0 X X X X X 251 3 172.342 175.172 116.972 1 250 251 3 163.7 178.077 120.556 1 250 260 3 167.804 171.79 118.449 1 259 269 3 166.087 168.566 117.232 1 268 276 3 164.687 175.581 122.283 1 275 280 3 158.704 170.241 121.059 1 279 290 3 163.014 158.719 120.648 1 289 291 3 150.895 147.086 121.425 1 290 291 3 148.41 139.677 122.901 1 290

0 0 X X X X X 252 3 171.955 173.521 121.675 1 251 252 3 163.148 174.712 124.944 1 251 261 3 167.252 168.425 122.837 1 260 270 3 164.13 166.664 121.991 1 269 277 3 162.73 173.679 127.042 1 276 281 3 158.973 168.102 125.623 1 280 291 3 161.01 156.363 125.755 1 290 292 3 151.295 142.599 127.281 1 291 292 3 146.758 135.93 128.531 1 291

0 0 X X X X X 253 3 171.759 171.792 126.363 1 252 253 3 162.018 173.189 129.736 1 252 262 3 166.048 167.279 127.564 1 261 271 3 165.211 163.878 126.35 1 270 278 3 163.811 170.893 131.401 1 277 282 3 158.157 166.106 129.823 1 281 292 3 158.947 154.91 130.01 1 291 293 3 150.912 139.889 130.925 1 292 293 3 146.139 133.056 131.776 1 292

0 0 X X X X X 254 3 171.834 170.214 131.107 1 253 254 3 161.952 170.765 134.154 1 253 263 3 165.982 164.855 131.982 1 262 272 3 166.1 161.605 131.36 1 271 279 3 164.386 169.425 136.38 1 278 283 3 158.732 164.638 134.802 1 282 293 3 156.853 151.467 135.592 1 292 294 3 150.103 137.832 134.11 1 293 294 3 145.33 130.866 135.137 1 293

0 0 X X X X X 255 3 171.908 168.635 135.85 1 254 255 3 161.069 168.525 139.012 1 254 264 3 165.893 163.409 137.347 1 263 273 3 165.22 160.44 136.834 1 272 280 3 163.682 166.316 141.141 1 279 284 3 158.028 161.529 139.563 1 283 294 3 155.751 149.683 139.405 1 293 295 3 148.552 135.79 136.773 1 294 295 3 143.779 128.824 137.8 1 294

0 0 X X X X X 256 3 171.132 166.425 140.154 1 255 256 3 160.442 167.403 143.298 1 255 265 3 164.609 160.206 141.944 1 264 274 3 163.608 158.247 142.19 1 273 281 3 164.467 164.456 146.95 1 280 285 3 157.983 160.075 145.841 1 284 295 3 153.89 147.034 142.038 1 294 296 3 148.374 132.08 140.3 1 295 296 3 143.955 125.559 141.662 1 295

0 0 X X X X X 257 3 171.075 164.657 144.831 1 256 257 3 160.385 165.635 147.975 1 256 266 3 163.287 156.823 146.016 1 265 275 3 162.086 154.542 146.352 1 274 282 3 163.329 161.237 151.657 1 281 286

3 156.845 156.856 150.548 1 285 296 3 152.752 143.815 146.745 1 295 297 3 147.236 128.861 145.007 1 296 297 3 142.914 121.938 146.21 1 296

0 0 X X X X X 258 3 171.07 162.62 150.047 1 257 258 3 160.38 163.598 153.191 1 257 267 3 162.664 154.031 149.799 1 266 276 3 161.706 151.827 149.631 1 275 283 3 162.949 158.522 154.936 1 282 287 3 156.527 155.189 154.139 1 286 297 3 151.792 142.255 150.088 1 296 298 3 146.706 125.901 148.146 1 297 298 3 142.671 118.962 149.338 1 297

0 0 X X X X X 259 -1 171 161 153 1 258 259 3 160.31 161.978 156.144 1 258 268 3 161.563 152.189 152.497 1 267 277 3 160.605 149.985 152.329 1 276 284 3 162.015 157.09 158.522 1 283 288 3 155.865 152.321 157.116 1 287 298 3 150.52 140.521 153.145 1 297 299 3 145.082 122.85 150.863 1 298 299 3 143.042 116.575 152.068 1 298

0 0 X X X X X 260 -1 171.298 159.842 157.648 1 259 260 3 160.061 159.741 160.267 1 259 269 3 161.314 149.952 156.62 1 268 278 3 160.785 146.555 156.51 1 277 285 3 160.826 154.586 163.304 1 284 289 3 154.707 149.63 161.845 1 288 299 3 149.547 137.408 158.033 1 298 300 3 144.498 120.239 154.412 1 299 300 3 142.387 113.792 155.527 1 299

0 0 X X X X X 261 -1 171.98 158.547 161.462 1.1 260 261 3 159.822 158.248 163.354 1.1 260 270 3 161.075 148.459 159.707 1.1 269 279 3 160.546 145.062 159.597 1.1 278 286 3 160.587 153.093 166.391 1.1 285 290 3 154.468 148.137 164.932 1.1 289 300 3 148.746 134.803 162.638 1.1 299 301 3 144.797 117.883 157.399 1.1 300 301 3 140.378 111.362 158.761 1.1 300

0 0 X X X X X 262 -1 172.001 156.708 165.57 1 261 262 3 159.843 156.409 167.462 1 261 271 3 160.544 146.926 163.885 1 270 280 3 160.655 143.106 164.413 1 279 287 3 160.732 150.698 169.706 1 286 291 3 154.498 146.001 168.725 1 290 301 3 147.242 132.44 166.353 1 300 302 3 144.296 114.067 159.738 1 301 302 3 138.86 108.161 163.054 1 301

0 0 X X X X X 0 0 X X X X X 263 -1 159.056 153.052 174.263 0.856931 262 272 3 159.302 142.369 170.364 0.856931 271 281 3 159.7 141.071 170.364 0.856931 280 288 3 159.777 148.663 175.657 0.856931 287 292 3 154.475 143.499 174.798 0.856931 291 302 3 146.301 128.717 170.617 0.856931 301 303 3 143.355 110.344 164.002 0.856931 302 303 3 137.616 105.641 166.927 0.856931 302

0 0 X X X X X 0 0 X X X X X 264 -1 158.959 150.498 178.561 0.774462 263 273 3 160.137 139.092 175.062 0.774462 272 282 3 160.137 139.092 175.062 0.774462 281 289 3 160.214 146.684 180.355 0.774462 288 293 3 154.111 141.413 179.718 0.774462 292 303 3 145.837 124.904 174.538 0.774462 302 304 3 142.891 106.531 167.923 0.774462 303 304 3 135.404 102.074 170.861 0.774462 303

0 0 X X X X X 0 0 X X X X X 265 -1 158.833 148.634 183.198 0.628867 264 274 3 160.897 137.19 178.514 0.628867 273 283 3 160.498 135.622 177.474 0.628867 282 290 3 160.171 145.099 184.077 0.628867 289 294 3 154.127 140.885 183.78 0.628867 293 304 3 145.215 120.48 177.377 0.628867 303 305 3 142.269 102.107 170.762 0.628867 304 305 3 135.354 98.6671 176.278 0.628867 304

0 0 X X X X X 0 0 X X X X X 266 -1 158.904 146.401 187.672 0.61871 265 275 3 160.815 136.373 182.193 0.61871 274 284 3 160.066 132.504 181.394 0.61871 283 291 3 159.94 141.974 189.618 0.61871 290 295 3 153.793 138.994 189.612 0.61871 294 305 3 145.636 118.43 180.431 0.61871 304 306 3 142.292 99.4853 173.78 0.61871 305 306 3 135.283 96.6073 179.804 0.61871 305

0 0 X X X X X 0 0 X X X X X X 267 -1 158.974 144.169 192.145 0.61871 266 276 3 162.266 134.068 186.753 0.61871 275 285 3 161.88 129.783 185.7 0.61871 284 292 3 160.965 140.546 194.55 0.61871 291 296 3 153.198 137.671 194.011 0.61871 295 306 3 145.041 117.107 184.83 0.61871 305 307 3 141.565 97.9721 178.166 0.61871 306 307 3 135.322 95.0685 184.014 0.61871 306

0 0 X X X X X 0 0 X X X X X X 268 -1 159.045 141.936 196.618 0.84236 267 277 3 162.956 131.654 190.599 0.84236 276 286 3 162.87 127.139 191.049 0.84236 285 293 3 162.58 137.913 200.169 0.84236 292 297 3 153.729 133.69 198.611 0.84236 296 307 3 146.346 114.755 190.061 0.84236 306 308 3 142.737 95.4296 183.385 0.84236 307 308 3 134.415 91.5549 188.71 0.84236 307

0 0 X X X X X 0 0 X X X X X X 269 -1 159.574 140.079 201.23 0.84236 268 278 3 163.83 128.502 194.64 0.84236 277 287 3 163.854 124.639 195.114 0.84236 286 294 3 161.21 136.478 203.864 0.84236 293 298 3 153.747 131.793 202.009 0.84236 297 308 3 146.364 112.858 193.459 0.84236 307 309 3 143.418 94.4854 186.844 0.84236 308 309 3 134.24 90.5438 192.174 0.84236 308

0 0 X X X X X 0 0 X X X X X X 270 -1 160.421 137.107 208.609 0.84236 269 279 3 165.094 124.779 200.662 0.84236 278 288 3 165.237 121.955 199.111 0.84236 287 295 3 162.468 134.161 208.066 0.84236 294 299 3 155.005 129.476 206.211 0.84236 298 309 3 145.766 110.91 200.03 0.84236 308 310 3 142.075 91.6409 192.007 0.84236 309 310 3 133.753 87.7662 197.332 0.84236 309

0 0 X X X X X 0 0 X X X X X X 0 0 X X X X X X 0 0 X X X X X X 289 -1 164.781 121.088 202.546 0.92118 288 296 3 162.012 133.294 211.501 0.92118 295 300 3 154.752 127.577 209.541 0.92118 299 310 3 145.496 110.05 204.831 0.92118 309 311 3 141.823 87.513 196.119 0.42118 310 311 3 132.335 85.3846 203.158 0.92118 310

0 0 X X X X X 0 0 X X X X X X 0 0 X X X X X X 0 0 X X X X X X 290 -1 165.866 120.332 205.821 0.5 289 297 3 163.097 132.538 214.776 0.5 296 301 3 155.412 125.822 212.226 0.5 300 311 3 146.677 107.803 209.159 0.5 310 312 3 141.183 84.3786 200.458 0.5 311 312 3 131.872 82.8031 206.78 0.5 311

0 0 X X X X X 0 0 X X X X X X 0 0 X X X X X X 0 0 X X X X X X 0 0 X X X X X X 298 -1 164 132.33 217.283 0.6 297 302 3 154.994 125.606 214.423 0.6 301 312 3 146.679 107.935 212.519 0.6 311 313 3 140.526 82.7381 203.894 0.6 312 313 3 132.523 78.2714 209.566 0.6 312

0 0 X X X X X 0 0 X X X X X X 0 0 X X X X X X 0 0 X X X X X X 0 0 X X X X X X 299 -1 164.5 131 219.833 0.5 298 303 3 155.494 124.276 216.973 0.5 302 313 3 146.853 106.722 215.132 0.5 312 314 3 140.7 81.5251 206.507 0.5 313 314 3 132.567 76.3882 211.778 0.5 313

0 0 X X X X X 0 0 X X X X X X 0 0 X X X X X X 0 0 X X X X X X 0 0 X X X X X X 300 -1 165 131 221.833 0.5 299 304 3 155.228 123.032 218.615 0.5 303 314 3 146.666 105.328 217.457 0.5 313 315 3 139.4 81.2163 208.804 0.5 314 315 3 132.17 74.7872 213.895 0.5 314

0 0 X X X X X 0 0 X X X X X X 0 0 X X X X X X 0 0 X X X X X X 0 0 X X X X X X 301 -1 165 131 224.667 0.5 300 305 3 155.168 120.864 220.462 0.5 304 315 3 147.209 104.854 221.383 0.5 314 316 3 138.749 80.4961 211.497 0.5 315 316 3 132.08 73.9436 216.624 0.5 315

0 0 X X X X X 0 0 X X X X X X 0 0 X X X X X X 0 0 X X X X X X 0 0 X X X X X X 302 -1 166 128.25 226.917 1 301 306 3 155.832 120.321 223.779 1 305 316 3 146.783 103.502 223.903 0.5 315 317 3 138.206 80.1007 213.492 0.5 316 317 3 132.12 73.7208 218.853 0.5 316

0 0 X X X X X 0 0 X X X X X 0 0 X X X X X 0 0 X X X X X 0 0 X X X X X 303 -1 166 130 229 0.5 302 307 3 157.841 117.361 224.452 0.5 306 317 3 146.984 103.223 225.535 0.5 316 318 3 138.126 79.1168 216.763 0.5 317 318 3 132.287 73.1512 221.348 0.5 317

#ESWCX: 10 columns (ID, Type/Event, X, Y ,Z, R, P, F-actin_Ratio, F-actin_Mean, F-actin_SD) each for T0 (0 min), T1(2 min), T2 (4 min), T3(6 min)

1 3 334.460895 318.583913 1.83910055 1.00595927 -1 0.321 51.2 29 1 3 334.513079 318.431512 2.1027187 1.00595927 -1 0.37 60.102 38.4 1 3 334.40456 318.54181 2.66065461 1.00595927 -1 0.501 49.5 24.04 1 3 334.40456 318.54181 2.66065461 1.00595927 -1 0.48 45 18

2 3 334.688014 317.491014 1.81110502 1.1033951 1 0.325581 53.5 29.2153 2 3 334.740198 317.338613 2.07472317 1.1033951 1 0.38 61.1053 38.3322 2 3 334.631679 317.448911 2.63265908 1.1033951 1 0.509091 49.6071 24.0451 2 3 334.631679 317.448911 2.63265908 1.1033951 1 0.482143 45.3704 17.864

3 3 334.992816 316.221401 1.51538185 1.13150923 2 0.326087 52.7333 31.7668 3 3 335.045 316.069 1.779 1.13150923 2 0.4 54.6364 33.8157 3 3 334.936481 316.179298 2.33693591 1.13150923 2 0.491525 47.3448 25.1551 3 3 334.936481 316.179298 2.33693591 1.13150923 2 0.483333 44.3103 16.114

4 3 334.992816 315.035401 1.51538185 1.04608165 3 0.384615 52.4 37.2 4 3 335.045 314.883 1.779 1.04608165 3 0.307692 28.375 7.71261 4 3 334.936481 314.993298 2.33693591 1.04608165 3 0.342105 62.4615 30.6936 4 3 334.936481 314.993298 2.33693591 1.04608165 3 0.378378 47.3571 16.7059

5 3 334.992816 313.849401 1.51538185 1.10285547 4 0.361111 60.4615 37.0833 5 3 335.045 313.697 1.779 1.10285547 4 0.296296 34.25 11.0538 5 3 334.936481 313.807298 2.33693591 1.10285547 4 0.40625 57.4615 30.3356 5 3 334.936481 313.807298 2.33693591 1.10285547 4 0.375 47.0833 17.1729

6 3 334.992816 312.663401 1.51538185 1.03175477 5 0.4 63.5714 35.4919 6 3 335.045 312.511 1.779 1.03175477 5 0.392857 33.2727 10.6608 6 3 334.789417 312.614775 2.35021318 1.03175477 5 0.4 59.4375 32.7681 6 3 334.928772 312.602322 2.35021318 1.03175477 5 0.4 43.5625 18.4593

7 3 334.992816 310.884401 1.51538185 0.99621035 6 0.355556 72.1875 35.7705 7 3 335.045 310.732 1.779 0.99621035 6 0.394737 30.9333 10.0828 7 3 334.646504 310.969793 2.34115807 0.69971035 6 0.457143 50.1875 33.2401 7 3 334.734861 310.969793 2.34115807 0.69971035 6 0.4375 39.5 18.9275

8 3 334.862356 309.704924 1.47379476 1.07791389 7 0.375 75.125 55.6816 8 3 335.045 309.546 1.779 1.07791389 7 0.439024 43 31.3457 8 3 334.564077 309.61123 2.36957463 1.07791389 7 0.44 56.5 44.3117 8 3 334.564077 309.61123 2.36957463 1.07791389 7 0.387755 47.1579 30.8771

9 3 334.329842 307.79072 1.51956843 1.200825 8 0.51145 99.9254 82.3537 9 3 334.382026 307.638319 1.78318658 1.200825 8 0.596273 109 83.516 9 3 334.112211 307.679236 2.32440582 1.200825 8 0.476563 95.541 79.499 9 3 334.112211 307.679236 2.32440582 1.200825 8 0.459016 84.2321 74.7555

10 3 333.806816 305.547401 1.21888185 0.96066 9 0.664063 109.118 86.7667 10 3 333.415436 305.652955 1.36296306 0.96066 9 0.554913 109.958 85.3855 10 3 333.444493 305.695058 1.94286369 0.96066 9 0.460432 97.7031 81.3491 10 3 333.444493 305.695058 1.94286369 0.96066 9 0.477273 83.6984 73.978

11 3 333.213816 303.768401 1.07063185 1.200825 10 0.329545 64.4138 64.5774 11 3 333.025835 303.65158 1.31962662 0.933975 10 0.258621 42.3333 31.3914 11 3 333.054892 303.693683

1.89952725 0.933975 10 0.322581 48.7 54.6005 11 3 333.054892 303.693683 1.89952725 0.933975 10 0.316667 38.3158 30.9636

12 3 333.131389 301.553546 1.19660877 1.0807425 11 0.313253 44 30.5602 12 3 333.183573 301.401145 1.46022692 1.0807425 11 0.333333 45.5833 24.4505 12 3 333.21263 301.443248 2.04012755 1.0807425 11 0.275 42.5909 21.4617 12 3 333.21263 301.443248 2.04012755 1.0807425 11 0.297297 40.6818 16.0181

13 3 333.213816 300.210401 1.21888185 1.14078375 12 0.26 49 28.8897 13 3 333.266 300.058 1.4825 1.14078375 12 0.363636 53 33.0333 13 3 333.295057 300.100103 2.06240063 1.14078375 12 0.290323 54.6667 25.0111 13 3 333.295057 300.100103 2.06240063 1.14078375 12 0.296296 48.375 17.2006

14 3 333.806816 298.431401 1.51538185 1.17080734 13 0.402174 66.0811 58.9793 14 3 333.859 298.279 1.779 1.17080734 13 0.37931 61.1364 58.9054 14 3 333.888057 298.321103 2.35890063 1.17080734 13 0.321429 69.1852 61.9863 14 3 333.888057 298.321103 2.35890063 1.17080734 13 0.305556 64.9091 54.4693

15 3 334.399816 296.652401 1.51538185 1.18581617 14 0.391304 85.7778 73.0985 15 3 334.708769 296.508895 1.78583729 1.18581617 14 0.46875 89.5333 68.5824 15 3 334.737826 296.550998 2.36573792 1.18581617 14 0.337349 75.9286 63.9899 15 3 334.737826 296.550998 2.36573792 1.18581617 14 0.302632 69.6522 55.0104

16 3 334.992816 295.466401 1.51538185 1.19332355 15 0.375 87.4444 69.9954 16 3 335.401393 294.955828 2.18248313 1.19332355 15 0.473118 81.9773 62.8275 16 3 335.624954 295.056638 2.78607411 1.19332355 15 0.39759 65.6061 37.8113 16 3 335.624954 295.056638 2.78607411 1.19332355 15 0.37037 62.7333 35.5986

17 3 336.178816 293.687401 1.81188185 0.81621706 16 0.402439 62.3636 50.4278 17 3 336.231 293.535 2.0755 0.81621706 16 0.517857 66.1724 49.4341 17 3 336.524535 293.537965 2.59934434 0.81621706 16 0.428571 54.5714 28.6183 17 3 336.524535 293.537965 2.59934434 0.81621706 16 0.463415 53.3158 34.3144

18 3 333.213816 307.919401 1.51538185 1.11971446 9 0.6375 111.471 90.7637 18 3 333.266 307.767 1.779 1.11971446 9 0.59596 136.966 88.2189 18 3 332.996185 307.807917 2.32021924 1.11971446 9 0.462366 117.791 84.6716 18 3 332.996185 307.807917 2.32021924 1.11971446 9 0.505747 97.0909 78.8623

19 3 331.933529 308.020211 1.50279246 1.19924762 18 0.517241 94.7556 81.2798 19 3 331.985713 307.86781 1.76641061 1.46609762 18 0.450704 119.828 83.0976 19 3 331.83094 308.074174 2.53503942 1.46609762 18 0.522523 88.569 59.8426 19 3 331.83094 308.074174 2.53503942 1.46609762 18 0.533333 79.2321 50.4837

20 3 330.209085 308.164903 1.49651852 1.20003631 19 0.402985 83.2222 72.6949 20 3 330.638417 308.01606 1.67769188 1.46688631 19 0.413333 104.145 78.0851 20 3 330.657986 308.17024 2.59475452 1.17038631 19 0.527132 86.9853 59.2431 20 3 330.657986 308.17024 2.59475452 1.17038631 19 0.512397 79.3871 51.5362

0 0 X X X X X X X X 21 -1 329.115 307.767 2.0755 1.26714019 20 0.336 97.5952 72.2848 21 3 329.115593 307.869589 2.67861065 1.26714019 20 0.612613 94.7353 65.7255 21 3 329.115593 307.869589 2.67861065 1.26714019 20 0.575472 93.1148 68.4134

0 0 X X X X X X X X 22 -1 327.929 307.174 2.372 1.43412306 21 0.372093 56.4688 51.7439 22 3 328.102156 307.2333 2.64706305 1.43412306 21 0.513333 86.6494 65.2726 22 3 327.958057 307.216103 2.95190063 1.43412306 21 0.510526 72.2784 63.3808

0 0 X X X X X X X X 0 0 X X X X X X X X 23 -1 326.866937 307.181709 2.87639987 1.43412306 22 0.517483 74.1757 62.1018 23 3 326.866937 307.181709 2.87639987 1.43412306 22 0.513514 76.0658 64.9044

0 0 X X X X X X X X 0 0 X X X X X X X X 24 -1 325.924067 307.229149 2.7466574 1.13762306 23 0.447619 67.4894 55.3034 24 3 325.924067 307.229149 2.7466574 1.13762306 23 0.491379 67.1053 58.6353

0 0 X X X X X X X X 0 0 X X X X X X X X 25 -1 325.07074 307.525649 2.90482829 1.16181153 24 0.380952 67.625 46.2032 25 3 325.07074 307.525649 2.90482829 1.16181153 24 0.392857 72.9091 57.6675

0 0 X X X X X X X X 0 0 X X X X X X X X 0 0 X X X X X X X X 26 -1 324.371 307.767 2.965 1.1739028 25 0.357143 67.8 56.4189

(*Syntax of basic SWC reconstructions in EBNF; terminal symbols are represented with double quotation marks*)

```
newline      = "\n" ;
letter       = "A" | "B" | "C" | "D" | "E" | "F" | "G"
               | "H" | "I" | "J" | "K" | "L" | "M" | "N"
               | "O" | "P" | "Q" | "R" | "S" | "T" | "U"
               | "V" | "W" | "X" | "Y" | "Z" | "a" | "b"
               | "c" | "d" | "e" | "f" | "g" | "h" | "i"
               | "j" | "k" | "l" | "m" | "n" | "o" | "p"
               | "q" | "r" | "s" | "t" | "u" | "v" | "w"
               | "x" | "y" | "z" ;
digit        = "0" | "1" | "2" | "3" | "4" | "5" | "6" | "7" | "8" | "9" ;
symbol       = "[" | "]" | "{" | "}" | "(" | ")" | "<" | ">"
                 | """ | "'" | "=" | "|" | "." | "," | ";" ;
integer      = [+|-] digit{digit} ;
double       = integer ["."] {integer} ;
character    = letter | double | symbol | "_" | " ";
header       = "#" character {character} newline ["#" character {character} newline] ;
Index        = integer ;
Type         = integer ;
X            = double ;
Y            = double ;
Z            = double ;
Radius       = double ;
Parent       = integer ;
node         = Index " " Type " " X " " Y " " Z " " Radius " " Parent ;
neuron_tree = node newline {node newline} ;
SWC          = [header] neuron_tree ;
```

(*Syntax of multi-signal ESWC reconstructions in EBNF; terminal symbols are represented with double quotation marks*)

```
newline       = "\n" ;
letter        = "A" | "B" | "C" | "D" | "E" | "F" | "G"
                | "H" | "I" | "J" | "K" | "L" | "M" | "N"
                | "O" | "P" | "Q" | "R" | "S" | "T" | "U"
                | "V" | "W" | "X" | "Y" | "Z" | "a" | "b"
                | "c" | "d" | "e" | "f" | "g" | "h" | "i"
                | "j" | "k" | "l" | "m" | "n" | "o" | "p"
                | "q" | "r" | "s" | "t" | "u" | "v" | "w"
                | "x" | "y" | "z" ;
digit         = "0" | "1" | "2" | "3" | "4" | "5" | "6" | "7" | "8" | "9" ;
symbol        = "[" | "]" | "{" | "}" | "(" | ")" | "<" | ">"
                | """ | "'" | "=" | "|" | "." | "," | ";" ;
integer       = [+|-] digit{digit} ;
double        = integer ["."] {integer} ;
character     = letter | double | symbol | "_" | " ";
header        = "#" character {character} newline ["#" character {character} newline] ;
Index         = integer ;
Type          = integer ;
X             = double ;
Y             = double ;
Z             = double ;
Radius        = double ;
Parent        = integer ;
Node          = Index " " Type " " X " " Y " " Z " " Radius " " Parent ;
Ratio         = double ;
Mean          = double ;
```

```
StdDev                  = double ;
Signal                  = [Ratio] Mean [StdDev];
Multisignal_Node        = Node " "  Signal {" "  Signal} ;
Multisignal_Neuron_Tree = Multisignal_Node newline {Multisignal_Node newline} ;
ESWC                    = [header] Multisignal_Neuron_Tree ;
```

(*Syntax of time-varying SWCX reconstructions in EBNF; terminal symbols are represented with double quotation marks*)

```
newline         = "\n" ;
letter          = "A" | "B" | "C" | "D" | "E" | "F" | "G"
                  | "H" | "I" | "J" | "K" | "L" | "M" | "N"
                  | "O" | "P" | "Q" | "R" | "S" | "T" | "U"
                  | "V" | "W" | "X" | "Y" | "Z" | "a" | "b"
                  | "c" | "d" | "e" | "f" | "g" | "h" | "i"
                  | "j" | "k" | "l" | "m" | "n" | "o" | "p"
                  | "q" | "r" | "s" | "t" | "u" | "v" | "w"
                  | "x" | "y" | "z" ;
digit           = "0" | "1" | "2" | "3" | "4" | "5" | "6" | "7" | "8" | "9" ;
symbol          = "[" | "]" | "{" | "}" | "(" | ")" | "<" | ">"
                  | """ | "'" | "=" | "|" | "." | "," | ";" ;
integer         = [+|-] digit{digit} ;
double          = integer ["."] {integer} ;
character       = letter | double | symbol | "_" | " ";
header          = "#" character {character} newline ["#" character {character} newline] ;
Index           = integer ;
Type            = integer ;
X               = double | "X" ;
Y               = double | "X" ;
Z               = double | "X" ;
Radius          = double | "X" ;
Parent          = integer | "X" ;
Event           = integer ;
T0_Node         = Index " " Type " " X " " Y " " Z " " Radius " " Parent ;
Tn_Node         = Index " " Event " " X " " Y " " Z " " Radius " " Parent ;
```

```
timevarying_node            = T0_Node {" " Tn_Node} ;
timevarying_neuron_tree = timevarying_node newline {timevarying_node newline} ;
SWCX                        = [header] timevarying_neuron_tree ;
```
