# Supplementary material for: Design and implementation of multi-signal and time-varying neural reconstructions
Source: Sci Data. 2018 Jan 23;5:170207. doi: 10.1038/sdata.2017.207 (PMC5779069; doi:10.1038/sdata.2017.207)
Supplement: Supplementary Figures [file sdata2017207-s2.pdf]

- **Supplementary Figure 1:** Schema of time-varying reconstruction. Digitized morphologies are represented as connected nodes (instead of connected frustums) for clarity of illustration. T0 is the first time point with all nodes represented in white. In T1, four events occur: terminal extension (left green nodes), interstitial extension (right green nodes), retraction (red nodes), and rotation (yellow node). In the SWCX file, the terminal extension from node 9 pushes nodes 10, 11, 12 in T0 to node 13, 14, 15 in T1). Interstitially extended nodes 16, 17, and 18 are inserted after node 15 at the end of the subtree, instead of right after the point of extension at node 14. Node 5 and 6 are tagged as retracted, but not deleted from the reconstruction. Node 15 rotates. In T2, no additional branches are inserted. Instead, node 5 and 6 re-emerge (in blue). Local scaling (stretching) occurs in node 3 and 4 (pink).

- **Supplementary Figure 2:** (a) Growing axon from the neonatal mouse cortex (Data Citation 2); same as Figure 3a, except the static (gray) P12 and P14 are not shown (b) Time-varying SWCX file (abridged for ease of illustration: darker row borders indicate discontinuities) with full details resulting in 21 columns in correspondence of the three time points from panel (a): the first seven columns represent P10, followed by seven columns for P12, and the last seven columns for P14. Second column in P10 represents neurite type (as in standard SWC files). However, the second column of P12 (9th overall) and P14 (16th overall) is changed to describe the type of dynamic event. Remaining columns (3-7, 10-14, 17-21) represent X, Y, Z, radius (R) and parent id (P) values from each time point. Morphological dynamics through the three time points are color-coded: green, elongation; red, retraction; blue, re-emergence; yellow, rotation; and pink, local scaling. Dynamic events on the SWCX rows have the same color-coding as the axonal arbors in panel (a). First time point (P10) has 110 nodes, second time point (P12) has 149 nodes, and the final time point (P14) has 194 nodes. The node indices of the root (in blue), branch points (in green), and terminations points (in yellow) are displayed for all three time points.

Suppl. Fig.1

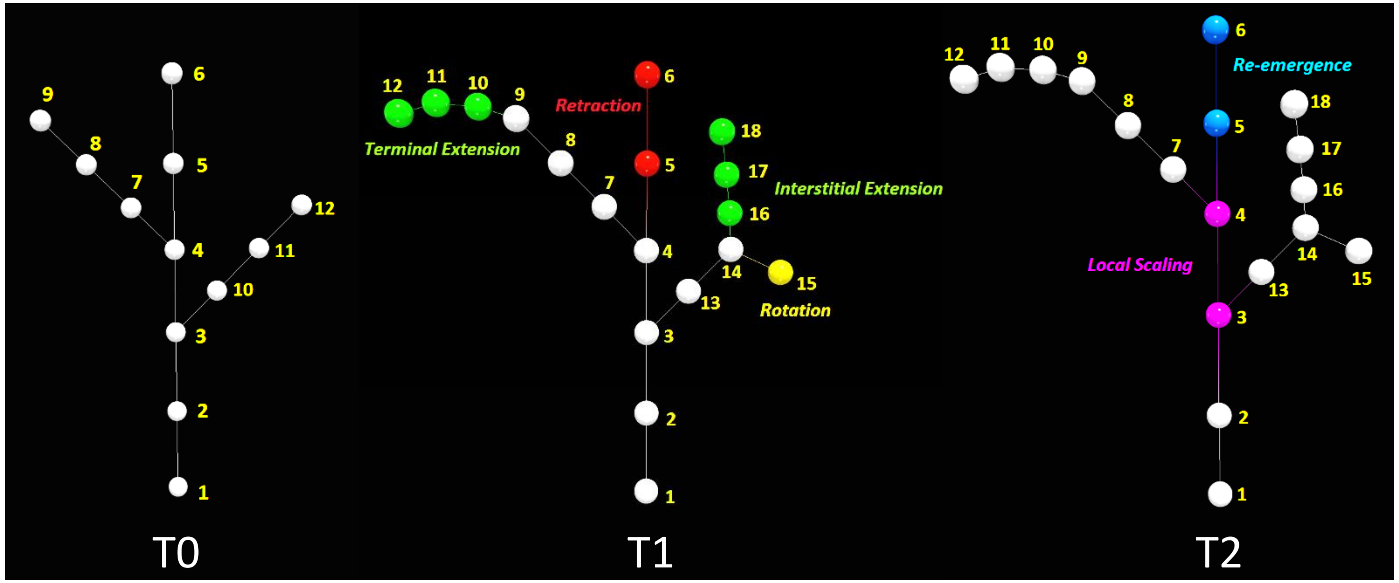

T0							T1							T2						
I	Type	X	Y	Z	R	P	I	Event	X	Y	Z	R	P	I	Event	X	Y	Z	R	P
1	2	3.00	1.00	0.00	0.15	-1	1	2	3.00	1.00	0.00	0.2	-1	1	2	3.00	1.00	0.00	0.2	-1
2	2	3.00	2.00	0.00	0.15	1	2	2	3.00	2.00	0.00	0.2	1	2	2	3.00	2.00	0.00	0.2	1
3	2	3.00	3.00	0.00	0.15	2	3	2	3.00	3.00	0.00	0.2	2	3	-2	3.01	3.21	-0.04	0.2	2
4	2	3.00	4.00	0.00	0.15	3	4	2	3.00	3.99	0.00	0.2	3	4	-2	3.01	4.39	-0.07	0.2	3
5	2	3.00	5.00	0.00	0.15	4	5	-4	3.00	5.00	0.00	0.2	4	5	-5	3.01	5.40	-0.07	0.2	4
6	2	3.00	6.00	0.00	0.15	5	6	-4	3.00	6.00	0.00	0.2	5	6	-5	3.01	6.40	-0.07	0.2	5
7	2	2.50	4.50	0.00	0.15	4	7	2	2.50	4.50	0.00	0.2	4	7	2	2.51	4.90	-0.07	0.2	4
8	2	2.00	5.00	0.00	0.15	7	8	2	2.00	5.00	0.00	0.2	7	8	2	2.01	5.40	-0.07	0.2	7
9	2	1.50	5.50	0.00	0.15	8	9	2	1.50	5.50	0.00	0.2	8	9	2	1.51	5.90	-0.07	0.2	8
0	0	X	X	X	X	X	10	-1	1.10	5.61	0.15	0.2	9	10	2	1.11	6.01	0.08	0.2	9
0	0	X	X	X	X	X	11	-1	0.68	5.63	0.32	0.2	10	11	2	0.69	6.03	0.24	0.2	10
0	0	X	X	X	X	X	12	-1	0.31	5.50	0.45	0.2	11	12	2	0.33	5.90	0.37	0.2	11
10	2	3.50	3.50	0.00	0.15	3	13	2	3.50	3.50	0.00	0.2	3	13	2	3.51	3.71	-0.04	0.2	3
11	2	4.00	4.00	0.00	0.15	10	14	2	4.00	4.00	0.00	0.2	13	14	2	4.01	4.21	-0.04	0.2	13
12	2	4.50	4.50	0.00	0.15	11	15	-3	4.60	3.73	0.03	0.2	14	15	2	4.60	3.94	-0.01	0.2	14
0	0	X	X	X	X	X	16	-1	3.97	4.43	0.03	0.2	14	16	2	3.98	4.64	-0.01	0.2	14
0	0	X	X	X	X	X	17	-1	3.92	4.87	0.08	0.2	16	17	2	3.93	5.09	0.04	0.2	16
0	0	X	X	X	X	X	18	-1	3.85	5.35	0.16	0.2	17	18	2	3.86	5.57	0.12	0.2	17

Suppl. Fig.2

Portera-Cailliau

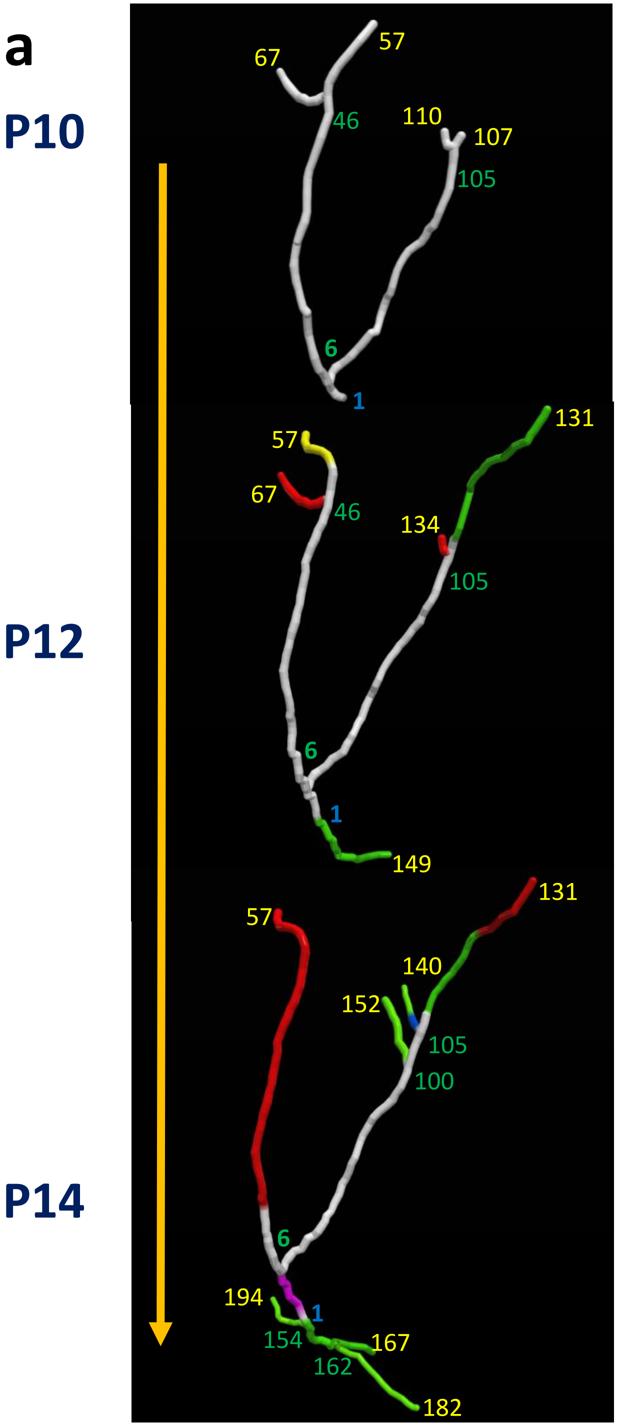

b

P10_I	Type	X	Y	X	R	P	P12_I	Event	X	Y	Z	R	P	P14_I	Event	X	Y	Z	R	P
1	2	197.1	169.2	93.4	2.3	-1	1	1	201.7	180.4	80.6	2.3	-1	1	2	195.5	182.2	80.9	2.3	-1
2	2	199.3	175.1	94.6	2.3	1	2	2	203.7	183.2	87.2	2.3	1	2	-2	206.5	185.9	96.2	2.3	1
6	2	221.1	197.5	109.5	2.3	5	6	2	222.2	200.0	109.7	2.3	5	6	-2	238.6	212.8	131.2	2.3	5
7	2	224.8	202.7	113.0	2.6	6	7	2	225.5	204.5	114.3	2.6	6	7	2	240.2	217.6	135.4	2.6	6
17	2	269.7	247.8	184.2	2.4	16	17	2	262.7	239.9	187.2	2.4	16	17	2	280.2	254.5	206.4	2.4	16
18	2	272.1	253.3	192.3	2.4	17	18	2	265.6	243.6	194.7	2.4	17	18	-4	283.1	258.2	213.9	2.4	17
19	2	273.0	262.0	194.6	2.5	18	19	2	268.6	248.3	199.2	2.5	18	19	-4	286.1	262.9	218.4	2.5	18
49	2	313.8	319.0	467.4	2.5	48	49	2	292.1	304.9	486.6	2.5	48	49	-4	309.6	319.5	505.7	2.5	48
50	2	311.2	321.1	476.8	2.3	49	50	2	294.1	306.7	492.6	2.3	49	50	-4	311.6	321.3	511.7	2.3	49
51	2	307.1	323.0	485.7	2.3	50	51	-3	299.8	303.6	502.5	2.3	50	51	-4	317.3	318.2	521.6	2.3	50
57	2	283.9	317.0	539.4	2.3	56	57	-3	337.1	275.3	535.2	2.3	56	57	-4	354.6	289.9	554.4	2.3	56
58	2	318.6	306.4	444.3	2.2	46	58	-4	294.6	293.8	458.6	2.2	46	58	0	X	X	X	X	X
59	2	325.6	303.7	437.7	2.5	58	59	-4	301.5	291.1	452.1	2.5	58	59	0	X	X	X	X	X
66	2	372.5	267.4	462.4	2.1	65	66	-4	348.5	254.7	476.8	2.1	65	66	0	X	X	X	X	X
67	2	379.4	267.4	469.7	2.1	66	67	-4	355.3	254.7	484.0	2.1	66	67	0	X	X	X	X	X
68	2	220.7	196.6	116.5	2.3	6	68	2	220.7	197.5	117.2	2.3	6	68	2	236.7	210.7	138.3	2.3	6
106	2	151.5	294.0	435.1	2.4	105	106	2	128.7	271.9	448.0	2.4	105	106	2	148.3	275.1	473.5	2.4	105
107	2	148.6	296.7	444.2	2.4	106	107	2	129.8	278.1	456.8	2.4	106	107	2	148.7	277.2	485.4	2.4	106
0	0	X	X	X	X	X	108	-1	129.0	286.0	461.0	1.9	107	108	2	147.8	277.7	490.8	1.9	107
0	0	X	X	X	X	X	109	-1	129.0	292.0	469.0	1.9	108	109	2	147.7	280.5	500.6	1.9	108
0	0	X	X	X	X	X	110	-1	129.0	295.0	481.0	1.9	109	110	2	146.2	283.4	511.7	1.9	109
0	0	X	X	X	X	X	120	-1	126.0	355.0	533.0	1.9	119	120	2	122.6	330.1	578.1	1.9	119
0	0	X	X	X	X	X	121	-1	121.0	367.0	534.0	1.9	120	121	-4	117.6	342.1	579.1	1.9	120
0	0	X	X	X	X	X	130	-1	99.0	434.0	563.0	1.9	129	130	-4	95.6	409.1	608.1	1.9	129
0	0	X	X	X	X	X	131	-1	98.0	437.0	569.0	1.9	130	131	-4	94.6	412.1	614.1	1.9	130
108	2	161.5	294.7	429.3	2.4	105	132	-4	136.1	270.8	441.6	2.4	105	132	-5	153.2	266.8	469.5	2.4	105
110	2	168.8	294.8	443.9	2.3	109	134	-4	143.4	271.0	456.3	2.3	133	134	-5	161.8	258.8	480.7	2.3	133
0	0	X	X	X	X	X	0	0	X	X	X	X	X	135	-1	165.0	251.0	491.0	1.8	134
0	0	X	X	X	X	X	0	0	X	X	X	X	X	136	-1	168.0	242.0	502.0	1.8	135
0	0	X	X	X	X	X	0	0	X	X	X	X	X	151	-1	195.0	216.0	507.0	2.4	150
0	0	X	X	X	X	X	0	0	X	X	X	X	X	152	-1	196.0	213.0	509.0	2.4	151
0	0	X	X	X	X	X	135	-1	198.0	176.0	75.0	2.3	1	153	2	190.6	180.3	75.0	2.3	1
0	0	X	X	X	X	X	136	-1	195.0	173.0	70.0	2.3	135	154	2	184.8	175.3	69.8	2.3	153
0	0	X	X	X	X	X	148	-1	83.0	34.0	20.0	2.3	147	166	2	79.7	37.9	34.8	2.3	165
0	0	X	X	X	X	X	149	-1	63.0	13.0	20.0	2.3	148	167	2	61.5	13.4	25.9	2.3	166
0	0	X	X	X	X	X	0	0	X	X	X	X	X	168	-1	133.0	125.0	44.0	1.8	162
0	0	X	X	X	X	X	0	0	X	X	X	X	X	182	-1	29.0	211.0	10.0	1.8	181
0	0	X	X	X	X	X	0	0	X	X	X	X	X	183	-1	188.0	173.0	74.0	2.3	154
0	0	X	X	X	X	X	0	0	X	X	X	X	X	194	-1	235.0	91.0	79.0	1.8	193
